# High-resolution regional climate projections and tourism impacts in the Macaronesian archipelagos

**DOI:** 10.1038/s41598-026-43092-9

**Published:** 2026-03-09

**Authors:** Jordi Rodríguez-Rull, Francisco J. Expósito, Juan P. Díaz, Judit Carrillo, Juan C. Pérez, Diamantino Henriques

**Affiliations:** 1 Grupo de Observación de la Tierra y la Atmósfera, Universidad de La Laguna, Canary Islands, Spain; 2 Departamento de Meteorologia e Geofísica, Instituto Português do Mar e da Atmosfera, Azores, Portugal

**Keywords:** High-resolution regional climate model, Tourism climate indices, Pseudo-global warming, Macaronesian archipelagos, Climate change impacts, Small islands, Climate sciences, Environmental sciences

## Abstract

Understanding the impacts of climate change in the North Atlantic Macaronesian archipelagos of the Azores, Madeira, the Canary Islands, and Cabo Verde is critical due to their economic reliance on tourism. In this study, a high-resolution Regional Climate Model (RCM) approach was applied, using the Weather Research and Forecasting (WRF) model driven by CMIP6 data through the pseudo-global warming (PGW) method. Focusing on the Holiday Climate Index for Beach tourism (HCIB), changes are projected for the mid-century (2030–2059) and end-of-century (2070–2099) against a recent past baseline (1990–2019) under both low-emissions (SSP1-2.6) and high-emissions (SSP5-8.5) scenarios. Projections demonstrate pronounced latitudinal and seasonal gradients: the northern archipelagos (the Azores and Madeira) reveal increased summer suitability, while southern archipelagos (the Canary Islands and Cabo Verde) indicate enhanced winter conditions, suggesting a seasonal shift in peak tourism conditions. Thermal comfort changes predominantly drive these trends, except in Cabo Verde, where aesthetic factors (cloud cover) are more influential. These findings provide valuable insights for developing location-specific climate adaptation strategies to support sustainable tourism in these vulnerable island regions.

## Introduction

The 2015 Paris Agreement established the global goal of limiting the increase in average temperature to well below 2${^{\circ }\hbox {C}}$ above pre-industrial levels, though recent data highlights the urgency of the challenge, with global temperatures exceeding the 1.5${^{\circ }\hbox {C}}$ threshold for prolonged periods^1–3^. This anthropogenic climate change significantly impacts vulnerable territories, particularly archipelagos characterized by small, fragmented oceanic islands with heterogeneous, complex terrain, and sensitive ecosystems. Understanding future climate change relies on Global Climate Models (GCMs), which simulate large-scale atmospheric processes. However, the relatively coarse spatial resolution of GCMs often limits their ability to accurately represent regional-to-local climate phenomena in areas with complex topography. To overcome these scale limitations, dynamical downscaling with Regional Climate Models (RCMs) enables a more realistic representation of mesoscale and local climate processes^4^.

The need for high-resolution downscaling is particularly acute in island systems like the Macaronesian archipelagos, where the intricate terrain and sharp altitudinal gradients generate pronounced microclimates. Processes such as orographic cloud formation, local wind channeling, and sea-breeze dynamics—which critically influence local temperature, sunshine duration, and wind speed—are key determinants of tourism suitability but are inadequately resolved by GCMs. Therefore, high-resolution RCM projections are essential to provide the actionable local detail required for robust impact assessments.

The North Atlantic Macaronesian archipelagos represent an ideal natural laboratory for assessing climate change impacts on small island systems. Tourism is the main economic driver across these islands, making the sector highly vulnerable to climatic shifts. Given the substantial contribution of beach, urban, and nature-based tourism to the Gross Domestic Product (GDP) of all four archipelagos^5–8^, the need for tailored, region-specific climate adaptation strategies to ensure sustainable tourism development highlights the need for high-resolution projections^9^. The high economic dependence on this sector makes these islands exceptionally sensitive to climate shifts, a vulnerability shared with many small island developing states in the Atlantic region^10^.

Tourism climate indices are used to quantitatively assess and evaluate the suitability of climatic conditions for various tourism activities^11^. This approach integrates multiple meteorological variables (e.g., temperature, precipitation, sunshine, and wind) into a single score, providing a standardized framework for assessing climatic attractiveness. While previous regional assessments have provided valuable insights into general climate trends, they often rely on coarse spatial resolution models. Building on the foundational Tourism Climate Index (TCI)^12^, this study employs three prominent indices: TCI, for general purpose tourism; the specialized Holiday Climate Index (HCI)^13^, for Urban (HCIU) and Beach tourism (HCIB); and the recently developed Camping Climate Index (CCI),^14,15^ for nature-based tourism. For detailed analysis, this study focuses primarily on the HCIB80 sub-index, where the suffix quantifies the average number of days per month the primary index score meets or exceeds a defined threshold (i.e., HCIB $\ge$ 80). The full set of indices (TCI, HCI, and CCI) is discussed in the Supplementary Information. Table 1 provides an overview of the scoring ranges and corresponding climatic conditions associated with these indices.

It is important to emphasize that the HCIB indicator and all other climate indices used in this study are based solely on climatic variables (temperature, humidity, precipitation, cloud cover, sunshine, and wind). While the impact of climate change on coastal dynamics (e.g., sea-level rise and resulting loss of dry beach area available for recreational use) is a critical component of assessing overall tourism risk, this factor is independent of the climatic conditions measured by the indices. Given the scope and methodological constraints of our regional climate model analysis, we focus strictly on the climatic suitability, and therefore coastal impacts such as beach loss are not included in the assessment of the projected changes to the tourism climate indices.

**Table 1:** Classification of tourism conditions based on TCI, HCI, and CCI scores.

TCI/HCI scores	Conditions for tourism	CCI scores	Conditions for tourism
80 to 100	Excellent (80 to 90) or ideal (90 to 100)	7 to 10	Optimal
60 to 80	Good (60 to 70) or very good (70 to 80)	5 to 7	Good
40 to 60	Marginal (40 to 50) or acceptable (50 to 60)	3 to 5	Acceptable
$<{40}$	Unfavorable	$<{3}$	Unfavorable

The table provides the interpretation for each index, categorizing suitability from unfavorable to ideal (TCI, HCI) or optimal (CCI).

The present work has two primary objectives, aiming to fill the methodological and geographical gaps identified above. First, to provide high-resolution climate projections for the Macaronesian archipelagos of the Azores, Madeira, the Canary Islands, and Cabo Verde. These projections leverage the Shared Socioeconomic Pathways (SSPs)^16,17^, covering two future periods (2030–2059 and 2070–2099) under two contrasting scenarios: a lower-emissions pathway (SSP1-2.6) and a higher-emissions pathway (SSP5-8.5). This establishes a solid scientific foundation for long-term planning that acknowledges the diverse climatic sensitivities within each archipelago. Second, to assess the main impacts of climate change on tourism across these archipelagos. This involves quantifying potential improvements or deteriorations in climatic conditions for tourism, thereby providing practical, stakeholder-relevant information for local governments and tourism operators.

For accomplishing these objectives, the dynamical downscaling approach was utilized with the Weather Research and Forecasting (WRF) model^18^, specifically its Advanced Research core (WRF-ARW). The pseudo-global warming (PGW) method simulated future climate scenarios^19^, forcing the WRF model with large-scale outputs from GCMs provided by the Coupled Model Intercomparison Project Phase 6 (CMIP6)^20^. This integrated RCM approach is crucial for translating global GCM signals into actionable, high-resolution regional impacts, a methodology that builds upon and extends previous regional work^21^. Furthermore, this study is closely aligned with the World Climate Research Programme—Coordinated Regional Climate Downscaling Experiment—Flagship Pilot Studies (WCRP CORDEX-FPS) initiative (FPS-I-Mac), which aims to deliver robust, policy-relevant information to adaptation communities and foster co-production of knowledge in these climatically sensitive archipelagos.

## Methods

### Study area

**Figure 1:**
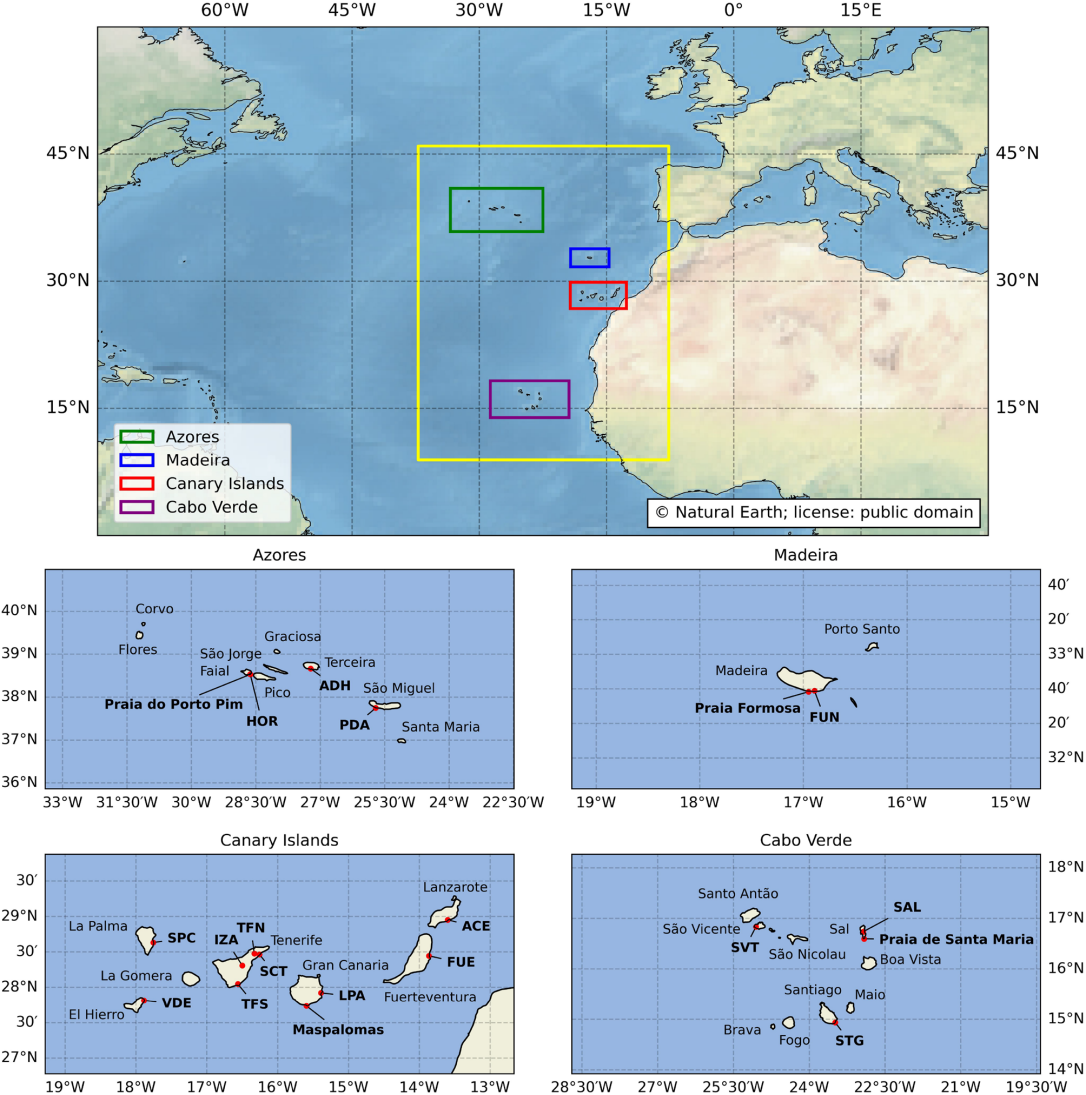
The Macaronesian region (outlined in yellow) is located off West Africa and Southern Europe, encompassing the archipelagos of the Azores (Portugal), Madeira (Portugal), the Canary Islands (Spain), and Cabo Verde. Insets zoom in on each archipelago, identifying major islands and highlighting ground-based meteorological stations used for model validation (“Validation of the regional climate model” section), as well as the specific beach locations (Praia do Porto Pim, Praia Formosa, Maspalomas, and Praia de Santa Maria) used for the sensitivity analysis (“Sensitivity analysis of the future changes in climatic variables” section). All locations are indicated by red dots and bold labels in the legend. Map created by the authors using Python 3.12.0 with Matplotlib 3.8.2 and Cartopy 0.22.0 libraries. The built-in base map imagery used by Cartopy was provided by Natural Earth raster and vector map data, which is in the public domain (Natural Earth Terms of Use).

Macaronesia is a group of four volcanic archipelagos located in the North Atlantic Ocean, off the coasts of West Africa and Southern Europe (Fig. 1). The region includes the Azores (Portugal), Madeira (Portugal), the Canary Islands (Spain), and the Republic of Cabo Verde. Politically, the islands belonging to Portugal and Spain are part of the European Union (EU). According to the European Environment Agency (EEA), the three European archipelagos form a single bioregion, known as Macaronesia or the Macaronesian Biogeographic Region. For the purpose of presenting and discussing the results, the Macaronesian archipelagos were aggregated into two groups based on observed latitudinal and climatic gradients: the northern archipelagos (the Azores and Madeira) and the southern archipelagos (the Canary Islands and Cabo Verde).

The Macaronesian archipelagos are home to a diverse and growing population. The Azores recorded a total population of 241,718 in 2024^6^, while Madeira had an estimated total population of 259,440^6^. The Canary Islands had the largest concentration, with an estimated total population of 2,238,574 in 2024, largely concentrated on the two central islands^22^. Cabo Verde had an estimated total population of 483,628 in its last census of 2021^23^.

Tourism emerges as the main economic driver in the archipelagos of Macaronesia, particularly beach, urban, and nature-based tourism, with the latest official estimates for 2023 and 2024 based on data from local governments and tourism offices confirming its substantial contribution to GDP. In the Azores, tourism has solidified its position as a vital economic pillar, now complementing the primary sectors of agriculture and fishing, which historically have had the highest contributions to GDP. The tourism sector demonstrated exceptional growth in 2024, marking its best year on record with a direct contribution estimated at around 17% of the regional GDP. This performance was driven by over 4.2 million overnight stays and more than 187 million euros in hotel industry revenue^5^. In Madeira, tourism plays an essential role in the economy. Preliminary data for 2024 indicate that tourist accommodation establishments registered 2.2 million guests and 11.7 million overnight stays^6^. In the Canary Islands, tourism is the cornerstone of the economy. In 2024, tourism accounted for 36.8% of the regional GDP, having reached 21.42 billion euros^7^. This represents an increase of 11% over 2023 levels in nominal terms. The economy of Cabo Verde is deeply reliant on its thriving tourism sector. In 2024, tourism’s direct contribution to the national GDP reached 25%^8^. Furthermore, tourism activity was a key catalyst for the year’s 7.3% overall economic growth, generating over 500 million euros in revenue and constituting more than 70% of the nation’s exports.

The four archipelagos exhibit diverse climatic patterns mainly influenced by their latitude, oceanic currents, prevailing winds, and orography^24,25^. Though generally categorized as subtropical, significant climatic differences exist across and within each archipelago. A major general influence common to all four territories is the Azores High, a semi-permanent high-pressure system that significantly regulates the entire region’s climate. Its strength and position dictate the path of North Atlantic depressions and the prevalence of trade winds^26^. The vast Atlantic Ocean moderates temperatures, preventing extremes of heat and cold. Specifically, the cold Canary Current particularly influences the western parts of the islands^27^, while the Gulf Stream contributes to the mild temperatures experienced, especially in Madeira and the Azores^28^. For the two northern archipelagos (north of ${30}{^{\circ }}$ N), the Azores High is, on average, centered to the southwest, which means predominant winds in the Azores are westerly for a large part of the year. In contrast, northeast trade winds are a dominant feature in the Canary Islands, especially during summer, bringing moisture to the windward (north and northeast) sides and creating drier conditions on the leeward (south and southwest) sides. Finally, the islands’ volcanic, mountainous terrain creates distinct microclimates, as higher altitudes experience cooler temperatures and increased precipitation due to orographic lift, leading to cloud formation and often a *sea of clouds* phenomenon^29^. Another important factor in the climate of the Macaronesian islands is their size. These are generally classified as *small islands*, where water (or moisture) is the most significant element, remaining constantly present whenever there is any wind.

In addition, there are some specific climatic characteristics per archipelago. The Azores experience a humid subtropical climate, predominantly temperate and moist, without a distinct dry season. Precipitation occurs year-round, with increased frequency and intensity during winter due to the North Atlantic storm track. The Azores also lie in the path of Atlantic tropical cyclones, especially between the months of September and October. Summers are sunnier but still humid. Temperatures are mild, typically ranging from 12 to 26${^{\circ }\hbox {C}}$, with extremes below 3${^{\circ }\hbox {C}}$ or above 30${^{\circ }\hbox {C}}$ being rare in populated areas^30,31^. Madeira also exhibits a mild to temperate climate with dry and warm summers^31^. The influence of the Gulf Stream and trade winds keeps temperatures mild year-round, averaging around 20${^{\circ }\hbox {C}}$ (with winter averages around 17${^{\circ }\hbox {C}}$ and summer averages around 24${^{\circ }\hbox {C}}$)^30^. However, easterly winds, due to the proximity of the African continent, generally bring drier air. Microclimates are pronounced due to the island’s mountainous topography. The northern and central mountainous regions are significantly wetter (2,500 to 3,000 mm annual rainfall) compared to the drier south and east (600 to 1,000 mm). The Canary Islands have a subtropical to arid climate. The eastern islands (Lanzarote and Fuerteventura) are particularly dry, exhibiting semi-desert or desert conditions due to their proximity to the Sahara and more influence from the Canary Current. The western islands, with higher altitudes, receive more rainfall. Overall, temperatures are warm and stable throughout the year, ranging from 18 to 24 ${^{\circ }\hbox {C}}$^32,33^. While average annual precipitation is low (rarely exceeding 250mm in most areas), the windward northeastern sides of the mountainous islands can receive up to 750mm, leading to lush vegetation zones, including *laurisilva* forests at higher elevations. Easterly winds can bring hot, dry air and dust from the Sahara, known as *calima*. Cabo Verde experiences a hot desert climate with a distinct dry season (December to July) and a warm, wet season (August to November) influenced by the West African Monsoon^34^. Despite its tropical latitude, the climate is not as hot or dry as continental regions at the same latitude due to oceanic moderation. Average annual temperatures for coastal areas are around 25${^{\circ }\hbox {C}}$, with minimums of 20 to 21 ${^{\circ }\hbox {C}}$ (January to April) and maximums of 26 to 28 ${^{\circ }\hbox {C}}$ (August to September)^35^. Rainfall is sporadic and varies significantly between the mountainous islands (up to 1,200mm annually in some places) and the flatter, older islands, which can experience prolonged droughts. Cabo Verde is also at the origin of Atlantic tropical cyclones^36^.

### Atmospheric model configuration and downscaling

WRF-ARW version 3.9 was used for recent past simulations and future climate projections over each study territory. The model was configured with three nested domains per archipelago at spatial resolutions of 27km, 9km, and 3km to perform the necessary dynamical downscaling from the larger scale to the regional domain. Vertically, 60 unevenly distributed levels were used, with the majority concentrated within the lowest troposphere. Initial and boundary conditions for this downscaling process were derived from an ensemble of 17 GCMs included in CMIP6 (Table 2), employing the PGW strategy^19^.

The PGW approach emphasizes reproducibility, as it allows for the isolation of the large-scale climate signal while maintaining consistency with validated present-day boundary conditions. The core of this method involves computing future boundary conditions by adding a mean climate perturbation signal (the global warming increment) to the same high-quality present-day reanalysis data used for the historical simulation. This approach, which is computationally efficient and consistent with the RCM configuration validated in previous high-resolution regionalization work for the Macaronesian region^37^, significantly diminishes errors in simulating the observed climate that often arise from biases in GCM boundary conditions. However, a limitation of the PGW method is that it does not account for internal climate variability or future changes in atmospheric circulation patterns beyond the mean warming signal^19^.

The global warming increment is estimated as the ensemble mean change computed from the CMIP6 GCMs. This use of a multi-GCM mean reduces projection uncertainty and is computationally efficient for RCMs, projecting climate changes by connecting them to large-scale GCM shifts without requiring direct nesting and full time-evolving GCM output for both historical and future periods. Initial and boundary conditions for the recent past period (1990–2019) used for RCM input were obtained from the ERA5 reanalysis datasets produced by the Copernicus Climate Change Service (C3S) at the European Center for Medium-Range Weather Forecasts (ECMWF). The details of the simulations as well as the parameterizations used to represent processes not explicitly resolved by the model can be found in Expósito et al^37^.

Future projections were conducted for two 30-year periods: a medium-term period (2030–2059) and a long-term period (2070–2099). To account for future greenhouse gas (GHG) emissions, the analysis focused on two contrasting SSP scenarios: SSP1-2.6 (sustainable development/low-emissions, with radiative forcing of 2.6W $\hbox {m}^{-2}$) and SSP5-8.5 (fossil fuel-based/high-emissions, with radiative forcing of 8.5W $\hbox {m}^{-2}$). This strategic selection, which follows CORDEX recommendations for regional climate impact studies, aims to define the maximum plausible range of projected change and associated risks. This ensures that the results effectively bracket the full scope of climate uncertainty driven by global mitigation efforts, providing the greatest relevance for regional policy and adaptation planning while managing the significant computational resources required for high-resolution RCM simulations.

**Table 2:** Overview of the CMIP6 ensemble of GCMs used as initial and boundary conditions for the downscaling process.

GCM	Institution(s) (country)	Horizontal resolution	References
ACCESS-ESM1-5	CSIRO and BOM (Australia)	$1.875^{\circ }\times 1.25^{\circ }$	Mackallah et al.^38^
AWI-CM-1-1-MR	AWI (Germany)	$\approx 0.9^{\circ }\times 0.9^{\circ }$	Semmler et al.^39^
BCC-ESM2-MR	BCC (China)	$1.1^{\circ }\times 1.1^{\circ }$	Wu et al.^40^
CanESM5	CCCma (Canada)	$\approx 2.8^{\circ }\times 2.8^{\circ }$	Swart et al.^41^
CESM2	NCAR (USA)	$1.25^{\circ }\times 0.9^{\circ }$	Danabasoglu et al.^42^
CMCC-ESM2	CMCC (Italy)	$2.5^{\circ }\times 1.25^{\circ }$	Lovato et al.^43^
EC-Earth3	EC-Earth Consortium (Europe)	$\approx 0.7^{\circ }\times 0.7^{\circ }$	Döscher et al.^44^
FGOALS-g3	CAS (China)	$2^{\circ }\times 2.3^{\circ }$	Li et al.^45^
GFDL-ESM4	GFDL (USA)	$\approx 1^{\circ }\times 1^{\circ }$	Krasting et al.^46^
GISS-E2-1-GN	GISS (USA)	$2.5^{\circ }\times 2^{\circ }$	Kelley et al.^47^
INM-CM5-0	INM (Russia)	$2^{\circ }\times 1.5^{\circ }$	Volodin et al.^48^
IPSL-CM6A-LR	IPSL (France)	$2.5^{\circ }\times 1.25^{\circ }$	Boucher et al.^49^
MIROC6	JAMSTEC, AORI, NIES, R-CCS (Japan)	$1.4^{\circ }\times 1.4^{\circ }$	Tatebe et al.^50^
MPI-ESM1-2-HR	MPI-M (Germany)	$\approx 0.9^{\circ }\times 0.9^{\circ }$	Jungclaus et al.^51^
MRI-ESM2-0	MRI (Japan)	$1.1^{\circ }\times 1.1^{\circ }$	Yukimoto et al.^52^
NorESM2-MM	NCC (Norway)	$1.25^{\circ }\times 0.9^{\circ }$	Selend et al.^53^
TaiESM1	AS-RCEC (Taiwan)	$1.25^{\circ }\times 0.9^{\circ }$	Lee et al.^54^

### Observational data and pre-processing for model validation

To ensure the reliability and accuracy of the regional climate model outputs, the downscaled meteorological variables were validated against observational data from a network of weather stations. A representative ensemble of 16 ground-based meteorological stations distributed across the four archipelagos was selected for this purpose, aiming to capture the diverse climatic conditions and topographic changes of the region. To ensure optimal comparisons, model coordinates closest to each station with the shortest horizontal offset were chosen. The observational data from these stations included key meteorological variables covering the recent past period (1990–2019) for which the model was simulated. These variables are directly relevant to the computation of the tourism climate indices. Table 3 provides a detailed overview of the selected testing stations, including their description, geographical coordinates, and altitude, together with their initial data availability and pre-processing rates.

Daily observational data for nine climate variables were required to calculate the tourism indices. These included mean, maximum, and minimum temperature (TG[${^{\circ }\hbox {C}}$], TX[${^{\circ }\hbox {C}}$], and TN[${^{\circ }\hbox {C}}$]); mean and minimum relative humidity (HUG[%], and HUN[%]); total precipitation (RR[mm]); sunshine (SS[h]); cloud cover (CC[%]); and mean wind speed (FG[${\hbox {m s}^{-1}}$]). Observational data were obtained from different sources depending on the study archipelago, mainly including the Instituto Português do Mar e da Atmosfera (IPMA) for the Azores and Madeira, and the European Climate Assessment & Dataset project (ECA&D) for the Canary Islands. These datasets are subject to rigorous, standardized quality control (QC) and homogenization procedures, as described in ECA&D Algorithm Theoretical Basis Document (ATBD)^55^. Data from IPMA adhere strictly to the instrumentation, calibration, and maintenance guidelines set by the World Meteorological Organization (WMO). Data obtained via ECA&D (a product of the European Meteorological Network (EUMETNET) member countries) are extensively vetted for inhomogeneities and systematic errors, ensuring the reliability and long-term representativeness of the observational time series used for model validation. In addition, where data availability was limited or insufficiently reliable, particularly for Cabo Verde, supplementary data were obtained from the European Organization for the Exploitation of Meteorological Satellites (EUMETSAT) Climate Monitoring Satellite Application Facility (CM SAF), which provided observational datasets from their satellite network, as well as from ECMWF C3S, which provided ERA5 reanalysis datasets. EUMETSAT provided data with a resolution of $0.05^\circ \times 0.05^\circ$ for the variables SS and CC, and ERA5 provided data with a resolution of $0.25^\circ \times 0.25^\circ$ for the variables TG, TX, TN, HUG, and RR, in addition to the mean wind speed components east-west UU[${\hbox {m s}^{-1}}$] and north-south VV[${\hbox {m s}^{-1}}$], from which FG was calculated. Except for IPMA, no major source of climate observation data generally provided HUN for the entire 30-year recent past validation period (1990–2019). Consequently, HUN was estimated from the difference between simulated HUG and HUN for each territory and meteorological station. This process yielded average correction factors of 8.7 for the Azores, 12.6 for Madeira, 14.0 for the Canary Islands and 16.3 for Cabo Verde. Where EUMETSAT and ERA5 data were unavailable, missing data were filled with estimates of monthly means from existing data, although these represented marginal average fill rates in the range of 0.00% to 0.08%.

The highest average data availability in the ground-based meteorological stations of the four study areas was found in the Canary Islands (98.07%), requiring very little additional data from satellites (0.41%), reanalysis (1.52%), and estimates (0.00%). Madeira had lower availability (53.36%), and consequently required more extensive filling with data from satellites (14.75%), reanalysis (31.89%), and estimates (0.08%). With slightly lower availability (47.44%), the Azores required more data filling from satellites (15.66%), reanalysis (36.90%), and estimates (0.08%). At the lowest end of data availability, reliable and adequate local data were not found for Cabo Verde, necessitating complete filling using data from satellites (22.22%), reanalysis (77.78%), and estimates (0.08%). Consequently, the entire missing data filling process achieved 100% useful data for the task of validating the model.

**Table 3:** Location of ground-based meteorological stations in the Macaronesian archipelagos of the Azores, Madeira, the Canary Islands, and Cabo Verde, used for model validation.

Meteorological station			Coordinates			Data availability (%)			
ID	Archipelago	Name	Lat.[°N]	Lon.[$^{\circ }$ W]	Hgt.[m]	Avail.	Sat.	Rea.	Est.
ADH	Azores	Angra do Heroismo Obs.	38.660	27.224	90	48.26	15.56	36.18	0.08
HOR	Azores	Horta Obs.	38.530	28.629	60	49.06	15.38	35.57	0.08
PDA	Azores	Ponta Delgada Airp.	37.744	25.708	77	45.01	16.03	38.96	0.08
FUN	Madeira	Funchal Obs.	32.649	16.893	25	53.36	14.75	31.89	0.08
FUE	Canary Islands	Fuerteventura Airp.	28.444	13.863	25	99.30	0.19	0.51	0.00
LPA	Canary Islands	Gran Canaria Airp.	27.922	15.389	24	98.29	0.90	0.81	0.00
VDE	Canary Islands	El Hierro Airp.	27.819	17.889	32	99.19	0.15	0.66	0.00
ACE	Canary Islands	Lanzarote Airp.	28.952	13.600	14	99.62	0.11	0.27	0.00
SPC	Canary Islands	La Palma Airp.	28.633	17.755	33	98.48	0.52	1.01	0.00
TFN	Canary Islands	Tenerife North Airp.	28.477	16.329	632	99.36	0.16	0.48	0.00
TFS	Canary Islands	Tenerife South Airp.	28.047	16.561	64	98.81	0.29	0.90	0.00
IZO	Canary Islands	Izaña Obs.	28.309	16.499	2371	91.37	1.30	7.33	0.01
SCT	Canary Islands	Santa Cruz Obs.	28.463	16.255	35	98.19	0.07	1.73	0.01
STG	Cabo Verde	Nelson Mandela Airp.	14.935	23.485	95	0.00	22.22	77.78	0.08
SAL	Cabo Verde	Amílcar Cabral Airp.	16.732	22.935	55	0.00	22.22	77.78	0.08
SVT	Cabo Verde	Cesária Évora Airp.	16.833	25.055	20	0.00	22.22	77.78	0.08

Each station’s data include its description, coordinates in decimal degrees (*Lat.* and *Lon.*), height in m a.s.l. (*Hgt.*), and the percentage of data availability (*Avail.*), comprising initial data and successive filling from satellite (*Sat.*), reanalysis (*Rea.*), and monthly average estimates (*Est.*).

### Tourism climate indices calculation and implementation

To assess the impacts of climate model projections on tourism in the four Macaronesian archipelagos, tourism climate indices were computed using simulated climatic data for a historical period (1990–2019) and two future periods (2030–2059 and 2070–2099) under two SSP scenarios (SSP1-2.6 and SSP5-8.5). Daily index values were derived for each 30-year period and scenario, from which monthly means were subsequently calculated. To capture seasonal variability and ensure a consistent sample size of 90 for the statistical process, monthly indices were grouped into boreal seasonal quarters: DJF (winter), MAM (spring), JJA (summer), and SON (autumn).

The meteorological variables required for computing the tourism indices were classified into three categories of climate data: thermal, physical, and aesthetic. Thermal comfort (TC) was selected as the primary metric for the thermal category, and it was calculated from the heat index equations (*feels like temperature*) as described by the National Oceanic and Atmospheric Administration (NOAA)^56^. For TCI, TC was defined as $TC = 4 \cdot CID + CIA$, where CID (Daytime Comfort Index) was derived from TX and HUN, and CIA (Daily Comfort Index) from TG and HUG. For HCIU and HCIB, TC was simplified to $TC = 4 \cdot CID$ and $TC = 2 \cdot CID$, respectively, where CID was obtained from TX and HUG. For CCI, TC was directly calculated from the heat index equations using TG and HUG. The physical category included the variables RR and FG, while the aesthetic category consisted of SS and CC. When SS was not available in hours, it was converted from the variable total daily solar radiation (accumulated down-welling shortwave flux at bottom available in ${\hbox {Jm}^{-2}}$ or ${\hbox {Wm}^{-2}}$), following the WMO definition of sunshine duration as the period during which direct solar irradiance exceeds a threshold value of 120W $\hbox {m}^{-2}$^57^. For the calculation of CCI, extreme values for the variables TX, TN, RR, and FG were considered, with the following cut-off values defined: TN=${8}{^{\circ }\hbox {C}}$, TX=${34}{^{\circ }\hbox {C}}$, RR=10mm, and FG=23km $\hbox {h}^{-1}$. In the absence of such extremes, i.e., if TN ${\ge 8}{^{\circ }\hbox {C}}$ and TX${\le 34}{^{\circ }\hbox {C}}$ and RR${\le 10}$mm and FG${\le 23}{\hbox {km h}^{-1}}$, CCI was obtained exclusively from TC and SS. Conversely, if an extreme value was found, i.e., if TN${< 8}{^{\circ }\hbox {C}}$ or TX${> 34}{^{\circ }\hbox {C}}$ or RR${> 10}$mm or FG${> 23}{km \hbox {h}^{-1}}$, then CCI was set to a limited maximum unfavorable threshold score of 3. Table 4 provides the list of the meteorological variables and formulas required to compute each tourism climate index.

While Table 4 provides the weighting formula for HCIB, the specific meteorological thresholds defining the suitability of conditions for beach tourism must also be clarified. Our analysis relies primarily on the HCIB80 sub-index, which denotes days where the overall score meets the “excellent” to “ideal” range (HCIB $\ge {80}$). Since HCIB is a weighted composite, a score of 80 is achieved when all four component variables consistently meet high rating thresholds. Table 5 details the meteorological criteria for each component that achieves a Rating $\ge$8 (on the 0 to 10 scale), which represents the minimum effective physical boundary required for “excellent” conditions. These ranges represent the component values that return a score of 8, 9, or 10 in the model. Values outside these ranges (e.g., $\text {Heat Index} < {26}{^{\circ }\hbox {C}}$ or $\text {Cloud Cover} \ge {46}{\%}$) receive lower scores, reducing the likelihood of the daily HCIB index reaching the 80 threshold.

**Table 4:** Formulas and meteorological variables used for calculating tourism climate indices.

Index	Formula	Meteorological variables
TCI	$0.5 \cdot \text {TC} + 0.2 \cdot \text {RR} + 0.2 \cdot \text {SS} + 0.1 \cdot \text {FG}$	TC, RR, SS, and FG (TC depends on TG, TX, HUG, and HUN)
HCIB	$0.2 \cdot \text {TC} + 0.3 \cdot \text {RR} + 0.4 \cdot \text {CC} + 0.1 \cdot \text {FG}$	TC, RR, CC, and FG (TC depends on TX and HUG)
HCIU	$0.4 \cdot \text {TC} + 0.3 \cdot \text {RR} + 0.2 \cdot \text {CC} + 0.1 \cdot \text {FG}$	TC, RR, CC, and FG (TC depends on TX and HUG)
CCI	$(0.5 \cdot \text {TC} + 0.5 \cdot \text {SS})$ or 3	TC, TX, TN, RR, SS, and FG (TC depends on TG and HUG)

Note that for CCI, the formula $(0.5 \cdot \text {TC} + 0.5 \cdot \text {SS})$ is used only when certain meteorological variables are within predefined favorable thresholds ($TN \ge {8}{^{\circ }\hbox {C}}$ and $TX \le {34}{^{\circ }\hbox {C}}$ and $RR \le {10}$  mm and $FG \le {23}\,{\textrm{km} \hbox {h}^{-1}}$). Otherwise, if any of these conditions are not met, CCI is assigned a fixed value of 3.

**Table 5:** Meteorological thresholds defining “excellent” beach tourism conditions (Component Rating $\ge 8.0$), based on the HCIB scoring system implemented in this study.

HCIB component	Meteorological variable	Component rating $\ge 8.0$	General conditions
Thermal	Heat index ($\text {HI}$)	${26}^{\circ }\hbox {C} \le \text {HI} < {34}^{\circ }\hbox {C}$	Warm to hot (but not sweltering)
Aesthetic	Cloud cover ($\text {CC}$)	$\text {CC} < 46\%$	Sunny to partly cloudy
Physical	Precipitation ($\text {RR}$)	$\text {RR} < {6}$ mm	No rain to very light rain
Physical	Wind speed ($\text {FG}$)	$\text {FG} < {30}{\textrm{km}\, \hbox {h}^{-1}}$	Calm to moderate breeze

These values represent the physical conditions required for each component to contribute maximally to the HCIB80 index threshold, based on the methodology established by Scott et al.^13^.

### Statistical methods

To assess the impacts of future climate change on tourism, hypothesis tests were conducted comparing tourism climate indices between historical (1990–2019) and future periods (2030–2059, 2070–2099 under SSP1-2.6 and SSP5-8.5 scenarios). Given the non-normal nature of climate data, statistical significance was determined using a bootstrapping method with percentiles^58^. This non-parametric approach is particularly suitable for climate data, which often exhibit complex or non-Gaussian distributions, allowing for significant inference without assumptions about the underlying data distribution. The bootstrap procedure was applied independently at each grid point. Modeled monthly values from the recent past and future periods were resampled with replacement to generate 1,000 bootstrap replicates for each period. For every bootstrap pair, we computed the difference between the future and historical means, thereby constructing an empirical distribution of the mean change. A mean change is estimated from this empirical distribution, along with its standard deviation ($\sigma$). The null hypothesis of no significant change was rejected (indicating a statistically significant climate change signal) if zero fell outside the $\pm 2\sigma$ interval of the bootstrap-derived distribution of mean differences, which approximates a two-sided 95% confidence level. This method directly assesses the significance of the projected changes, with areas of non-significant change visually indicated by hatching in the results.

## Results and discussion

### Validation of the regional climate model

Before using the model projections for future analyses, a comprehensive validation was conducted for each study territory to establish the model’s accurate representation of the recent past and provide a credible basis for future projections. The primary objective was to quantitatively compare the model’s simulated outputs for the recent past (1990–2019) with available observational datasets. Observed and simulated tourism climate indices for this period were compared using monthly mean plots and quantile-quantile (Q-Q) plots across all ground-based meteorological stations. A more comprehensive set of validation plots is available in the Supplementary Information.

**Figure 2:**
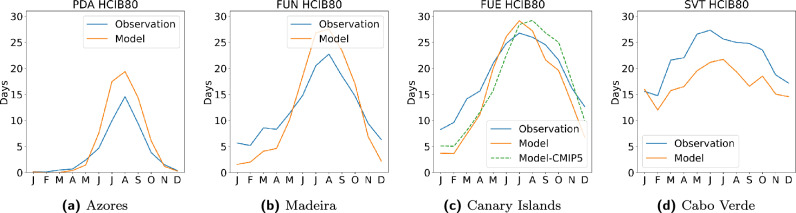
Validation of simulated indices. Monthly average plots compare simulated distributions and observed data for the 1990–2019 period from ground-based meteorological stations in the Azores, Madeira, the Canary Islands, and Cabo Verde. Figures display the HCIB80 sub-index (number of days per month with HCIB $\ge {80}$) for a representative station from each archipelago, selected for its relevance to beach-based tourism. In the Canary Islands, an additional line Model-CMIP5 represents a comparative average of three runs driven by three different GCMs included in the former CMIP5 framework used in a previous work^21^ based on a different reference period (1980–2009).

For a representative illustration, Fig. 2 shows the monthly comparison of the HCIB80 sub-index (average days per month with an HCIB value of 80 or higher) at a key beach-tourism station from each archipelago. For the Canary Islands, Fig. 2c additionally includes HCIB80 data from a previous work for methodological comparison^21^. This earlier work also utilized the same WRF-ARW RCM, but it differed in the reference period (1980–2009 instead of the present 1990–2019) and in its execution strategy, which involved multiple runs using three GCMs (GFDL-ESM2M, IPSL-CM5A-MR, and MIROC-ESM) from the former CMIP5 framework as driving boundary conditions. The additional Model-CMIP5 data shown in Fig. 2c represent the average of these three simulations. For station PDA in the Azores (Fig. 2a), the model exhibits small deviations, demonstrating strong agreement with observations. During the winter, the model’s number of excellent days is nearly identical to the observation. In spring, there is a negligible tendency towards overestimation. This positive bias becomes consistent during summer and autumn, where the model consistently predicts a slightly higher number of excellent days than what was observed, accurately reproducing the seasonal cycle but slightly overstating the peak magnitude. The model shows a pronounced seasonal dependence in its bias for station FUN in Madeira (Fig. 2b). During winter, the model significantly underestimates the observed count of excellent days. Spring marks a rapid transition where the model shifts from underestimation to a clear overestimation by the end of the season. The summer peak is marked by strong overestimation, with the model simulating substantially more excellent days than observed. Finally, the model shows a distinct drop-off in autumn, resulting in a systematic underestimation of conditions compared to the observations. For station FUE in the Canary Islands (Fig. 2c), the model fit is generally strong, with minor biases. Winter sees a slight but consistent underestimation of excellent days. Spring represents a transition period where the model tracks below the observation but converges toward the line. In summer, the model shows a small overestimation, slightly exceeding the magnitude of the observed peak. Autumn is largely neutral, with the model generally tracking slightly below the observation with minimal sustained bias. The model for station SVT in Cabo Verde (Fig. 2d) displays a strong and pervasive negative bias across all seasons. Throughout all seasons, the model is systematically and noticeably positioned below the observation line. This consistent pattern indicates a systematic underestimation of the number of excellent beach tourism days in every season, confirming a major systematic cold/wet bias in the simulation for this region.

**Figure 3:**
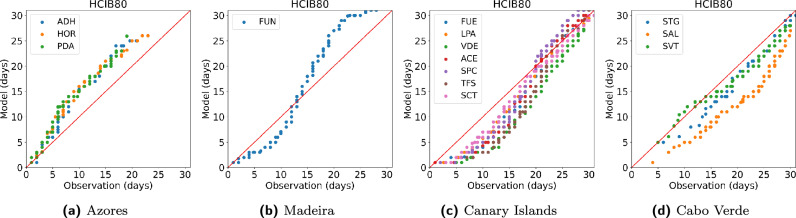
Validation of simulated indices (Q–Q plots). Q–Q plots compare the distributions of the HCIB80 sub-index (number of days per month with HCIB $\ge {80}$) from model simulations against observed data, using data from ground-based meteorological stations across the Macaronesian archipelagos (the Azores, Madeira, the Canary Islands, and Cabo Verde) during the 1990–2019 period. Note that non-coastal and elevated stations (IZO and TFN) in the Canary Islands have been excluded. The X- and Y-axes represent observed and simulated values (days), respectively, with the red line indicating ideal 1:1 agreement.

The model performance was further evaluated through a combination of Q–Q plots (Fig. 3), which provide a global comparison of the HCIB80 sub-index across all stations within each archipelago, and quantitative statistical metrics (Table 6), including bias, Root Mean Square Error (RMSE), and Pearson Correlation (*R*) for indices TCI80, HCIU80, HCIB80, and CCI07.

The model validation for the Azores (Fig. 3a) shows high skill in reproducing the observed distribution, which is supported by the lowest mean RMSE values (Table 6) among the archipelagos. The data points closely track the ideal 1:1 line throughout the low-to-mid range of monthly excellent days, indicating accurate simulation for the majority of the year. However, in the highest range (20 to 31 days), representing peak summer months, the points cluster slightly above the 1:1 line. This suggests the model has a minor tendency to overestimate the frequency of the absolute best conditions. The validation plot for Madeira (Fig. 3b) reveals a clear systematic bias. The data points adhere reasonably well to the 1:1 line only in the lowest frequency range. For the mid-to-high range (15 to 31 days), the curve bends sharply and consistently above the ideal line. This strong deviation indicates that the model systematically overestimates the frequency of excellent beach tourism days, simulating a distribution that is significantly biased toward higher monthly counts compared to the observed record. The model demonstrates good overall performance for the Canary Islands (Fig. 3c), particularly for the extreme ends of the distribution. However, a noticeable clustering of points occurs below the 1:1 line in the mid-range (15 to 25 days). This indicates that the model tends to underestimate the frequency of excellent days during shoulder seasons. Despite this minor bias, the model captures the lowest and absolute highest frequency counts accurately, as the points converge back toward the line at both ends of the plot. The validation for Cabo Verde (Fig. 3d) shows the weakest validation among the four archipelagos. The data points from all stations are consistently and noticeably positioned below the ideal 1:1 line across the entire distribution, from the lowest to the highest monthly counts. This pattern signifies a major, systematic underestimation (a cold/wet bias) of the frequency of excellent beach tourism days, meaning the model simulates fewer excellent days than were actually observed.

Statistical performance metrics are detailed in Table 6, which summarizes the average bias, RMSE, and *R* values for indices TCI80, HCIU80, HCIB80, and CCI07 across the ground-based meteorological stations for the 1990–2019 period. The model simulations demonstrate a reasonable ability to reproduce observations, with varying performance across the different sub-index configurations. HCIB80 exhibits the most robust performance overall, showing the lowest mean RMSE (6.1), a low mean bias of -2.3, and consistently high correlation values across the primary tourism regions. Notably, in the Canary Islands coastal stations (e.g., FUE, LPA, and ACE), where TCI80 and HCIU80 show weaker temporal correlations ($R < {0.55}$), HCIB80 maintains strong agreement with observations (R ranging from 0.75 to 0.85). This indicates that the model is particularly skillful at capturing the variability of beach tourism conditions. Given this superior predictive performance, HCIB was selected as the primary index for presenting the figures and main conclusions in this manuscript; results for the remaining indices are included in the Supplementary Information.

HCIU80 and TCI80 also indicate comparable and good overall performance in terms of error magnitude, with mean RMSE values of 6.6 and 6.7, respectively, and mean biases of − 1.8 and − 1.5. However, their capacity to reproduce temporal variability is spatially dependent; they achieve excellent correlations in the Azores ($R > {0.90}$) and high-altitude stations but perform poorly in capturing the variability at coastal Canary Islands stations. In contrast, CCI07 shows a higher mean RMSE (8.1) and a larger positive mean bias (4.0), indicating a tendency to overestimate observations. Furthermore, CCI07 displays the most erratic correlation structure, ranging from high skill at station IZO ($R = {0.89}$) to a negative correlation at station STC ($R = {-0.62}$), suggesting considerable uncertainty for this index.

**Table 6:** Quantitative assessment of model performance: bias, RMSE, and Correlation (*R*) for TCI80, HCIU80, HCIB80, and CCI07 sub-indices across ground-based meteorological stations of the Macaronesian archipelagos of the Azores, Madeira, the Canary Islands, and Cabo Verde in the 1990–2019 period.

Observations		Model simulations											
Station		TCI80			HCIU80			HCIB80			CCI07		
ID	Hgt. (m)	Bias	RMSE	R	Bias	RMSE	R	Bias	RMSE	R	Bias	RMSE	R
ADH	90	− 0.4	3.2	0.94	0.9	3.9	0.92	1.9	3.4	0.87	4.0	5.2	0.81
HOR	60	0.3	3.5	0.94	0.6	4.1	0.91	1.0	3.0	0.90	3.8	4.9	0.84
PDA	77	− 1.1	3.2	0.95	0.2	3.6	0.93	1.7	3.3	0.85	3.6	4.8	0.81
FUN	25	− 4.4	9.4	0.68	− 1.7	8.4	0.58	− 0.1	7.3	0.73	4.3	7.3	0.75
FUE	25	− 2.2	7.5	0.53	− 1.1	7.5	0.05	− 2.6	5.5	0.85	9.2	10.2	0.59
LPA	24	0.2	7.0	0.43	− 0.1	6.5	0.22	− 2.4	5.6	0.80	5.1	6.4	0.55
VDE	32	− 1.5	6.9	0.66	− 3.8	7.1	0.45	− 5.7	7.5	0.78	4.0	6.8	0.15
ACE	14	− 0.3	8.3	0.27	1.7	8.2	0.02	− 2.3	6.2	0.75	3.6	5.8	0.56
SPC	33	− 1.0	6.6	0.70	− 2.0	5.6	0.57	− 2.2	6.0	0.83	18.0	18.6	0.61
TFN	632	− 1.2	5.4	0.87	− 1.2	4.7	0.87	− 0.7	4.5	0.84	5.7	7.8	0.50
TFS	64	0.9	6.0	0.32	0.5	6.2	0.08	− 3.5	6.5	0.69	5.9	7.6	0.35
IZO	2371	− 1.7	2.7	0.95	− 2.2	3.6	0.94	− 1.8	2.6	0.93	1.0	3.6	0.89
SCT	35	− 0.4	5.5	0.54	− 0.5	4.4	0.57	− 1.9	5.3	0.82	− 2.1	12.9	− 0.62
STG	95	− 3.7	9.9	0.55	− 4.8	9.3	0.72	− 4.8	9.8	0.35	− 9.3	12.2	− 0.01
SAL	55	− 3.8	10.7	0.28	− 6.9	11.8	0.40	− 8.8	12.1	0.01	7.3	10.7	0.30
SVT	20	− 3.9	10.8	0.18	− 6.0	11.4	0.34	− 4.7	9.3	0.38	0.6	4.1	0.59

The *Hgt.* column under *Station* refers to the height of the meteorological station (m a.s.l.).

While most indices demonstrate reasonable performance, particularly in the Azores, several major deviations indicate specific areas where model accuracy is low. The most notable deviation is observed with CCI07 at station SPC, which exhibits an exceptionally high positive bias (18.0) and the largest RMSE (18.6). Station SAL consistently presents major negative deviations, with HCIB80 exhibiting a large negative bias (− 8.8) and the largest RMSE for the index (12.1). This is accompanied by a negligible correlation ($R = {0.01}$), indicating that the model fails to capture the inter-annual variability of beach conditions at this location.

Overall, the ensemble-mean RMSE across all stations and sub-indices is 6.35, with an ensemble-mean bias of -0.36, indicating good model performance for the tourism-relevant climate metrics. Performance presents a clear latitudinal gradient. The Azores show the highest model skill, characterized by the lowest errors (mean $RMSE = {3.84}$) and excellent temporal correlations (mean $R > {0.90}$). Intermediate skill is demonstrated by the Canary Islands (mean $RMSE = {6.64}$), where correlations are index-dependent but strong for beach tourism. Conversely, the lowest skill is found in Cabo Verde (mean $RMSE = {10.17}$), which is compounded by generally weak correlations (mean $R < {0.40}$) and large underestimations. However, it is worth noting that Cabo Verde is the archipelago with the fewest available observations (Table 3).

### Sensitivity analysis of the future changes in climatic variables

**Figure 4:**
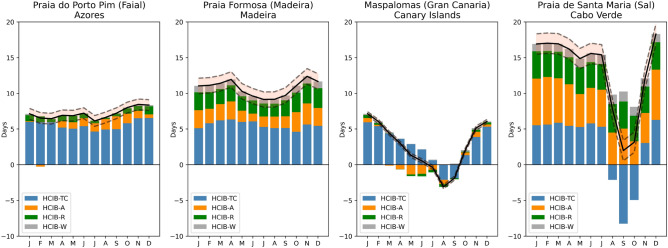
Projected changes in HCIB (black line) for 2070–2099 under the SSP5-8.5 emissions scenario vs. 1990–2019. The shaded area (in coral red) surrounding the projected change represents the model uncertainty, quantified as the standard deviation derived from the baseline HCIB simulation validation (“Validation of the regional climate model” section). The figure also displays the monthly contributions of HCIB-TC (thermal comfort), HCIB-A (cloud cover or aesthetic), HCIB-R (precipitation), and HCIB-W (wind). Data are shown for representative beach locations (from left to right) of Praia do Porto Pim (${38.525}{^{\circ } \hbox {N}}$, ${28.626}{^{\circ } \hbox {W}}$) on the island of Faial (the Azores); Praia Formosa (${32.638}{^{\circ } \hbox {N}}$, ${16.950}^{\circ } \hbox {W}$) on the island of Madeira; Maspalomas (${27.736}^{\circ } \hbox {N}$, ${15.594}^{\circ } \hbox {W}$) on the island of Gran Canaria (the Canary Islands); and Praia de Santa Maria (${16.595}^{\circ } \hbox {N}$, ${22.913}^{\circ } \hbox {W}$) on the island of Sal (Cabo Verde).

Figure 4 illustrates the monthly contributions of individual components to the projected HCIB changes, alongside the model’s baseline uncertainty to enable a comprehensive assessment of change magnitude. Analysis of the mean annual contributions across the four representative locations reveals the dominant influence of thermal comfort. This thermal component’s dominance, particularly in the northern archipelagos, is primarily driven by rising temperatures, as current summer conditions there generally remain below the heat-stress threshold, allowing warming to increase suitability. The dominance of thermal comfort remains consistent across all sites, with the sole exception of Praia de Santa Maria (Cabo Verde).

In Praia do Porto Pim (the Azores), the thermal comfort component is overwhelmingly the primary driver, accounting for an average change of 77.99% of the total projected HCIB change. Other components, such as aesthetic (11.66%), precipitation (8.95%), and wind (1.40%), project much smaller average changes, confirming the relevant role of rising heat stress in the Azores. Praia Formosa (Madeira) similarly shows thermal comfort as the largest factor, with an average contribution of 51.85%. However, the contributions of aesthetic (19.37%) and precipitation (21.34%) are also notable here, alongside wind (7.44%). The data confirm that while thermal comfort is dominant, these other factors, particularly precipitation, introduce significant seasonal variability. For Maspalomas (the Canary Islands), the average thermal comfort change of 103.76% is significantly higher in magnitude than the average changes in aesthetic (− 8.29%), precipitation (− 0.47%), or wind (5.00%). This pronounced annual average is largely driven by substantial positive contributions during the off-peak season, which are partially offset by a notable decrease in thermal comfort during the peak months of August and September. This clearly indicates that thermal comfort is the primary driver of projected HCIB changes, both positive and negative, in this location. Lastly, Praia de Santa Maria (Cabo Verde) presents a unique pattern, with a much lower average thermal comfort contribution of 20.93% compared to the other locations. Here, the aesthetic component (cloud cover) is the primary driver with an average contribution of 41.14%, often alongside substantial positive precipitation contributions (28.71%). This suggests a more balanced influence of factors in Cabo Verde, where the projected reduction in cloudiness is the dominant effect on beach tourism suitability. These results underscore that rising heat stress (HCIB-TC) is the predominant global factor driving projected alterations in beach tourism climatic suitability across Macaronesia, with its high average percentage changes reinforcing it as a critical challenge. Cabo Verde is the single exception, where the aesthetic component is most influential.

### Climate change signals for seasonal tourism

**Figure 5:**
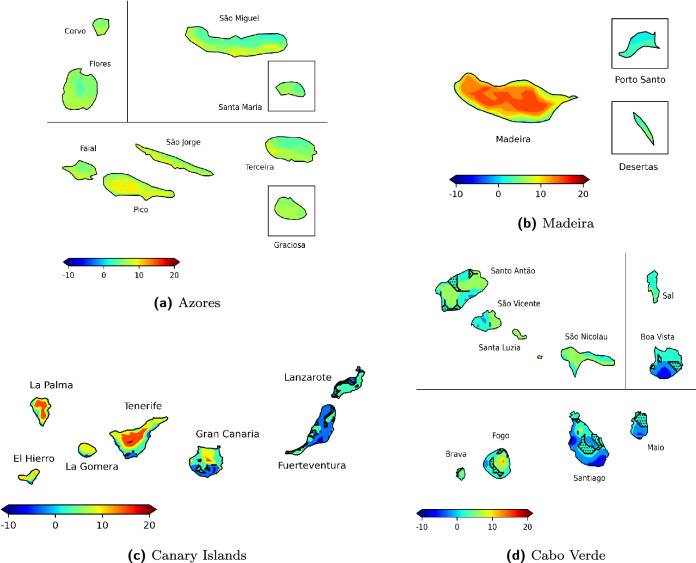
Projected changes in the HCIB80 sub-index (number of days per month with HCIB $\ge {80}$) for summer (JJA) across the Azores, Madeira, the Canary Islands, and Cabo Verde. Projections are for 2070–2099 under the SSP5-8.5 scenario, shown as the average monthly change in days relative to the 1990–2019 baseline. Areas with black dots indicate statistically non-significant changes, while non-hatched areas indicate statistically significant changes. These non-significant results reflect variability in the ensemble response rather than absence of change. Map created by the authors using Python 3.12.0 with Matplotlib 3.8.2 and Cartopy 0.22.0 libraries. The built-in base map imagery used by Cartopy was provided by Natural Earth raster and vector map data, which is in the public domain (Natural Earth Terms of Use).

**Figure 6:**
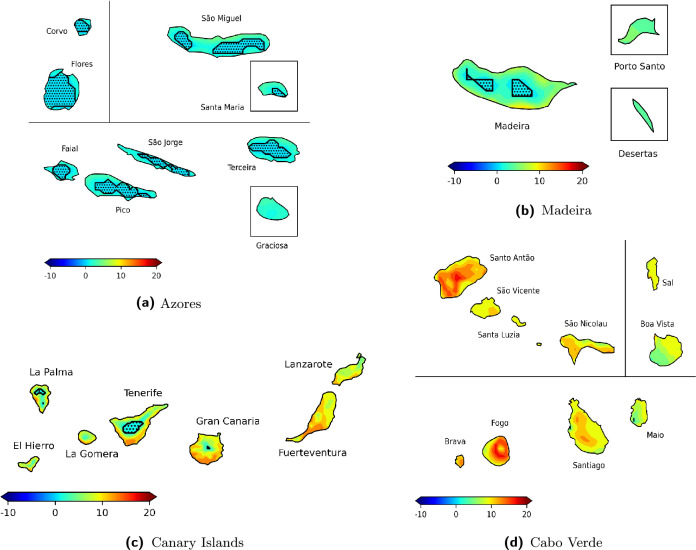
Projected changes in the HCIB80 sub-index (number of days per month with HCIB $\ge {80}$) for winter (DJF) across the Azores, Madeira, the Canary Islands, and Cabo Verde. Projections are for 2070–2099 under the SSP5-8.5 scenario, shown as the average monthly change in days relative to the 1990–2019 baseline. Areas with black dots indicate statistically non-significant changes, while non-hatched areas indicate statistically significant changes. These non-significant results reflect variability in the ensemble response rather than absence of change. Map created by the authors using Python 3.12.0 with Matplotlib 3.8.2 and Cartopy 0.22.0 libraries. The built-in base map imagery used by Cartopy was provided by Natural Earth raster and vector map data, which is in the public domain (Natural Earth Terms of Use).

To illustrate the signals of climate change and their potential impacts on tourism, the most unfavorable situation was selected: the end of the century (2070–2099) under the worst-case change scenario (SSP5-8.5). The projected changes in the sub-index HCIB80 for this scenario are shown in Figs. 5 and 6. HCIB80, a key indicator of thermal comfort for beach-based tourism, is highly relevant for assessing the future viability of this economic driver in the Macaronesian archipelagos, and it represents the average change in days per month with excellent beach tourism conditions during the summer (JJA) and winter (DJF) seasons. These maps present the calculated changes at the native 3km resolution of the dynamical downscaling model, with no post-processing spatial interpolation applied.

Analysis of future projections for JJA (Fig. 5) reveals statistically significant climate change signals across all four archipelagos, though with varying spatial extent. All projected changes in excellent beach tourism days during summer in the Azores are statistically significant. All islands exhibit a general increase in excellent conditions, with some localized areas showing more substantial improvements. Madeira exhibits statistically significant changes across its entire area, with no non-significant regions. The main island depicts an overall increase in excellent beach tourism days, though some coastal areas, particularly in the south and west, project less pronounced improvements. The island of Porto Santo also projects increases. In the Canary Islands, the summer season generally shows a widespread increase in excellent beach tourism days. Gran Canaria mostly increases across its northern and eastern parts, while its southern region projects unique decreases. Tenerife displays uniform increases, although decreases are observed in dispersed areas of the south and east. Fuerteventura’s eastern coast generally sees increases, whereas its western parts exhibit decreases. Lanzarote similarly projects increases, predominantly in its southern and eastern regions, but its northern areas present decreases. The smaller islands of La Gomera, La Palma, and El Hierro generally project increases. Across all the Canary Islands, scattered non-significant regions are present. Cabo Verde projects statistically significant signals across its area, with many coastal regions across various islands showing increases in excellent beach tourism days. Conversely, decreases are projected to affect key beach-tourism areas, particularly in the southern parts of the islands of Santiago, Maio, and Boa Vista. Scattered non-significant areas are also present across the archipelago.

The analysis for DJF (Fig. 6) reveals statistically significant climate change signals across all four archipelagos, though with varying degrees of spatial significance. The Azores display changes in excellent beach tourism days predominantly as increases. However, a significant characteristic across nearly all islands is the extensive presence of non-significant regions, indicating that these projected changes are largely not statistically significant. While the changes are generally positive, their prevalence of non-significance suggests a less certain climate change signal for excellent beach tourism conditions during winter in the Azores. Madeira projects statistically significant increases across most areas, though with notable non-significant regions on the main island. The main island of Madeira predominantly shows increases, with a considerable portion, especially in the central-eastern part, exhibiting stronger increases. Distinct non-significant regions are present in the central-western areas of the main island. The islands of Porto Santo and Desertas both display uniform increases, with no non-significant areas. The Canary Islands generally reveal statistically significant increases across broad areas. Gran Canaria mostly shows increases, with its southern coastal areas exhibiting particularly strong increases. Tenerife displays extensive increases. Fuerteventura and Lanzarote both project extensive increases. The smaller islands of La Gomera, El Hierro, and La Palma generally experience increases. Non-significant changes are present to some degree across all islands. Cabo Verde projects statistically significant increases across all islands. The most substantial gains are projected for Fogo and Santo Antão, which stand out with very strong increases across most of their areas. The remaining islands also exhibit predominant increases in excellent conditions.

A more comprehensive set of projection maps, including sub-indices TCI60, TCI80, HCIU60, HCIU80, HCIB60, HCIB80, CCI05, and CCI07, is available in the Supplementary Information.

### Latitudinal patterns

**Figure 7:**
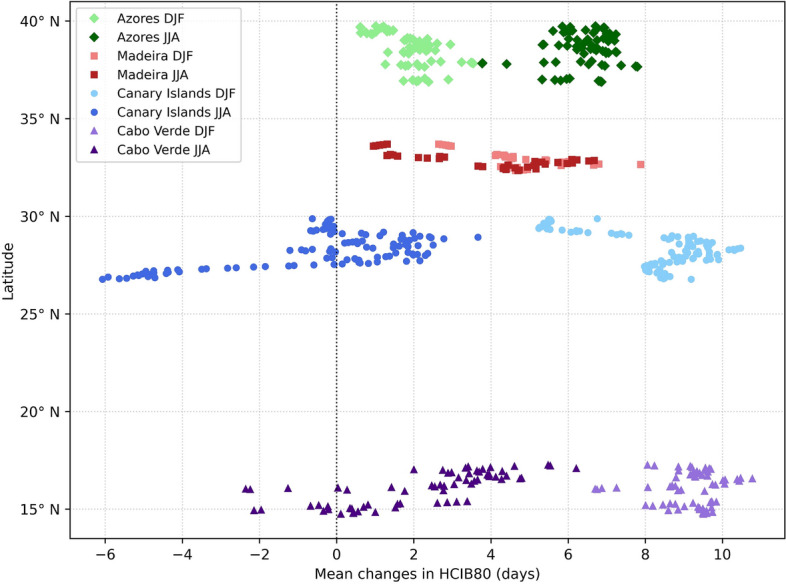
Latitudinal projected changes in the HCIB80 sub-index (number of days per month with HCIB $\ge {80}$) for 2070–2099 (SSP5-8.5) relative to the recent past (1990–2019). Data is filtered to show results only for low-elevation coastal land ($0 < \text {elevation} \le 250 \text { m}$), explicitly excluding ocean-only grid points ($\text {elevation} = 0 \text { m}$), to represent the most relevant areas for beach tourism. The plot uses individual markers instead of lines to enhance clarity.

The analysis of projected changes in the HCIB80 sub-index reveals distinct latitudinal patterns across the Macaronesian archipelagos, with changes between the summer (JJA) and winter (DJF) seasons. The data presented were filtered to include only areas of coastal land, which are considered the most relevant for this beach oriented sub-index. The latitudinal mean change is presented in Fig. 7. A general increase in beach tourism suitability (positive change in days) is observed across all archipelagos, but clear seasonal and latitudinal differences exist. The Azores is the only archipelago that projects consistently higher improvements during the summer compared to winter. Conversely, in the Canary Islands and Cabo Verde, the winter season shows a higher increase in suitability than the summer. Madeira exhibits the smallest variation between its summer and winter projections. The southern archipelagos (the Canary Islands and Cabo Verde) indicate the largest overall magnitude of positive change, with the best projections found during the winter season. Both archipelagos demonstrate the largest dispersion between their summer and winter suitability projections. It is noteworthy that despite the strong overall positive trends, the southern archipelagos demonstrate more nuanced patterns, with localized segments exhibiting a decrease in suitability (negative change in days), specifically during the summer season.

### Discussion

The results of this study, derived from climate projections through a high-resolution regional climate model (WRF-ARW) using established tourism climate indices, confirm significant climate change signals in the Macaronesian archipelagos, with direct implications for tourism. Comprehensive model validation confirms the reliability of these projections, offering a credible foundation for understanding future climate change signals in this tourism-dependent region.

Results unequivocally project statistically significant climate change signals impacting tourism in the most unfavorable situation: the end of the century (2070–2099) under the worst-case scenario of change (SSP5-8.5). Predominantly, these manifest as improvements in climate suitability for excellent beach tourism (HCIB80), though with heterogeneous spatial and seasonal distributions, including some localized deteriorations. Notably, the greatest contribution to this signal is the increase in thermal comfort resulting from the rise in temperature and humidity. Contrary to previous assumptions that warming would generally degrade beach tourism conditions, our projections indicate regionally enhanced suitability, largely driven by temperature increases that shift the thermal comfort optimum northward. This finding notably departs from previous general assumptions of widespread negative impacts on beach tourism due to global warming, particularly regarding thermal discomfort^59^.

Analysis of projected HCIB80 changes reveals a general enhancement of beach tourism suitability, driven by increases in excellent days, with distinct latitudinal patterns across the archipelagos that vary between summer (JJA) and winter (DJF). This pattern is further visualized in Fig. 7, which restricts the elevation data to the $0 < \text {elevation} \le 250 \text { m}$ range, representing the low-elevation coastal land most relevant for beach tourism activities.

During the summer, all four archipelagos generally project improvements. The northern archipelagos (the Azores and Madeira) consistently display widespread and statistically significant increases in excellent conditions. The Azores project strong gains, all significant, while Madeira also exhibits widespread significant increases with no projected decreases. In contrast, the southern archipelagos (the Canary Islands and Cabo Verde) predominantly indicate widespread statistically significant increases but present more complex and mixed signals, including localized areas of significant decrease. The Canary Islands project overall significant increases, yet specific significant decreases appear in southern Gran Canaria, central/western Fuerteventura, northern Lanzarote, and dispersed areas of southern/eastern Tenerife, where most of the current tourism infrastructure is located. Cabo Verde projects strong, significant increases across many coastal areas, but also includes some very localized significant decreases and scattered non-significant areas. This summer pattern indicates that all archipelagos become more favorable for beach tourism, with the northernmost regions showing more uniform significant increases, while southern ones exhibit a broader range of responses, including specific localized declines.

For the winter season, increased beach tourism suitability also persists, but with a more pronounced latitudinal gradient in magnitude and significance. The southern archipelagos (Cabo Verde and the Canary Islands) consistently display widespread and highly statistically significant increases. Cabo Verde stands out with particularly strong and pervasive significant increases, with virtually no non-significant regions. The Canary Islands also project widespread significant increases, though with scattered non-significant areas and varying magnitudes across islands (e.g., strong gains in southern Gran Canaria vs. more moderate increases in Tenerife and La Palma central areas). Moving northward, Madeira projects widespread significant increases, but its main island contains distinct, sizable areas where changes are not statistically significant. The northernmost archipelago (the Azores) represents a different extreme in winter; projected changes are predominantly increases but are largely characterized by widespread non-significance. This pervasive lack of statistical significance suggests higher uncertainty in the winter climate change signal of the Azores compared to more confident significant projections further south. In summary, winter projections indicate a strong latitudinal gradient, with the most significant and confident improvements concentrated in the tropical/subtropical south, progressively giving way to more moderate and less statistically significant changes towards the temperate north.

Regarding the acceptability of model uncertainty, a comparison of the mean RMSE of HCIB80 for the Azores with the natural inter-annual variability (standard deviation of HCIB80 monthly means over the historical period) confirms that the model error is comfortably within the range of natural climate fluctuations, suggesting the model is an acceptable tool for climate projection.

A final, critical point derived from the validation is the clear latitudinal gradient in model performance (“Validation of the regional climate model” section). The lowest errors were found in the Azores (north), while the highest errors, associated with a systematic cold/wet bias, occurred in Cabo Verde (south). This pattern is likely a function of the dominant meteorological regimes and known challenges in RCMs. The northern archipelagos (the Azores and Madeira) are largely controlled by mid-latitude synoptic systems (like the Azores High), which are generally well-resolved by RCMs. Conversely, the southernmost archipelago (Cabo Verde) is dominated by tropical dynamics where highly localized and intense convective processes prevail. Accurately simulating these explicit convection-permitting regimes at 3km resolution is notoriously challenging for RCMs, often resulting in systematic biases (e.g., overestimation of cloudiness and humidity), which manifest as the systematic underestimation (cold/wet bias) of “excellent” tourism days observed in Cabo Verde.

The projected shifts in climate suitability carry significant implications for Macaronesian tourism planning and adaptation. Projected widespread increases suggest opportunities for extended tourism seasons and potentially increased visitor numbers, especially during traditional off-peak periods. This may necessitate managing consequences like increased resource demand and potential overcrowding. For regions experiencing localized decreases, adaptive measures remain crucial, potentially involving tourism diversification or specific beach management strategies. The high confidence in significant increases for Cabo Verde, particularly in winter, positions it as a potential beneficiary. Conversely, the high uncertainty in the Azores’ winter projections highlights the need for continued monitoring and localized studies. The analysis underscores the importance of granular, location-specific climate impact assessments for resilient tourism. Ultimately, adaptation strategies should consider not only mitigating adverse impacts but also leveraging potential shifts in seasonal demand.

Although our study focuses strictly on the climatic suitability for tourism, it is necessary to acknowledge that the projected rise in sea level will have direct consequences on tourism infrastructure and coastal assets in the Macaronesian archipelagos. Previous studies conducted for mainland Portugal^60^ have excluded the Portuguese archipelagos of the Azores and Madeira. In the Azores, an index that combines exposure to coastal risks from the sea, inherent biophysical characteristics (geomorphology and landforms), acquired characteristics (coastal defenses), and socioeconomic characteristics^61^ has been developed. However, to the authors’ knowledge, no specific studies have been published in Madeira or Cabo Verde that quantify coastal vulnerability, shoreline retreat, or projected impacts of sea level rise. The Government of the Canary Islands^62^ estimates that by 2050, 147 tourist beaches will experience severe degradation, including a projected 10.6% loss of dry beach area due to sea level rise. The estimated direct losses could reach 11% of current GDP in the worst-case scenario by the year 2100. In addition, this rise will affect cultural assets—archaeological, historical, and ethnographic—that are most vulnerable to flooding, coastal erosion, and related risks^63^. The impact of sea level rise on tourism is beyond the scope of this study; however, it must be included in adaptation strategies, and specific studies should be undertaken given its importance, not only for this sector but also for infrastructure and cultural heritage.

## Supplementary Information

Supplementary Information.

## Data Availability

The datasets generated and/or analyzed in this study are openly available (Rodriguez-Rull, Jordi; Expósito González, Francisco Javier; Diaz, Juan P; Carrillo, Judit; Pérez Darias, Juan Carlos; Henriques, Diamantino (2026), “Tourism Index Data for Macaronesia”, Mendeley Data, V1, doi: 10.17632/5xrst4z9gk.1) under a CC BY 4.0 license.
