# Supplementary material for: High-resolution regional climate projections and tourism impacts in the Macaronesian archipelagos
Source: Sci Rep. 2026 Mar 9;16:8696. doi: 10.1038/s41598-026-43092-9 (PMC12979854; doi:10.1038/s41598-026-43092-9)
Supplement: Supplementary file 1 — Supplementary Information. [file 41598_2026_43092_MOESM1_ESM.pdf]

High-Resolution Regional Climate Projections and Tourism Impacts in the Macaronesian Archipelagos

(Supplementary Information)

Jordi Rodríguez-Rull[1,*], Francisco Javier Expósito[1], Juan Pedro Díaz[1], Judit Carrillo[1], Juan Carlos Pérez[1], and Diamantino Henriques[2]

[1]Grupo de Observación de la Tierra y la Atmósfera, Universidad de La Laguna, Canary Islands, Spain.
[2]Departamento de Meteorologia e Geofísica, Instituto Português do Mar e da Atmosfera, Azores, Portugal.
[*]Corresponding author: alu0101616742@ull.edu.es

1 Introduction

This supplementary information is a companion to the manuscript "**High-Resolution Regional Climate Projections and Tourism Impacts in the Macaronesian Archipelagos**". This document provides additional results that support the findings and conclusions of the main paper. The data underpinning this material are openly available [1] under a CC BY 4.0 license.

The primary purpose of this document is to extend the assessment of climate change impacts on tourism by presenting comprehensive figures and tables. The analysis relies on high-resolution regional climate model projections, which are first validated against historical data and then used to project future changes in tourism climate indices. Specifically, this supplementary information is structured as follows:

2. Regional climate model validation: This section details the validation of the regional climate model, demonstrating its skill in accurately simulating historical climate conditions for 1990–2019 using both monthly average plots and quantile-quantile (Q-Q) plots.

3. Projections of tourism climate indices: This section presents projections of tourism climate indices, which analyze the anticipated changes in tourism suitability for the archipelagos in the near and long-term future under different climate scenarios. These maps project simulated values from the 1990–2019 baseline and future changes for 2030–2059 and 2070–2099 under both the SSP1-2.6 and SSP5-8.5 scenarios, with visual indications of statistically significant and non-significant geographical areas. Maps were created by the authors using Python 3.12.0 with Matplotlib 3.8.2 and Cartopy 0.22.0 libraries. The built-in base map imagery used by Cartopy was provided by Natural Earth raster and vector map data, which is in the public domain (Natural Earth Terms of Use).

4. Summary of climate projections for tourism in the Macaronesian archipelagos: This section provides a synthesis of the projected changes in tourism suitability, specifically analyzing the trends for multiple climate indices (TCI, HCIU, HCIB, and CCI) across the four archipelagos (the Azores, Madeira, the Canary Islands, and Cabo Verde) and highlighting the divergent impacts of the SSP1-2.6 and SSP5-8.5 scenarios on seasonal tourism conditions.

2 Regional climate model validation

The validation of the regional climate model against observed data from ground-based meteorological stations during the 1990–2019 period is presented through two complementary approaches. The locations of these stations in the Macaronesian archipelagos, along with the coordinates and height of the corresponding model grid points used for validation, are detailed in Table S1. The first method uses monthly average plots to evaluate the model's performance for the TCI60, HCIU60, HCIB60, and CCI05 sub-indices, which correspond to good tourism conditions (Figure S1), as well as the TCI80, HCIU80, HCIB80, and CCI07 sub-indices, which represent excellent or optimal conditions (Figure S2). The second approach, employing Q-Q plots, provides a detailed comparison of the full distributions of the simulated values against the observed data (Figure S3 and Figure S4).

Overall, the model demonstrates a high degree of skill in simulating past observations. In the monthly average plots (Figure S1 and Figure S2), the model's output consistently and closely tracks the observed data. This strong correspondence signifies that the model successfully captures both the seasonal patterns and the magnitude of the observed data for all four indices across the Azores, Madeira, the Canary Islands, and Cabo Verde. The model accurately simulates the seasonal cycle of the indices, from the characteristic low values in winter to the increases in spring and autumn, and the peak values observed during the summer months. A particularly noteworthy finding from this analysis is the significant improvement in the Canary Islands, where

Table S1. Validation stations and model nearest locations in the Macaronesian archipelagos of the Azores, Madeira, the Canary Islands, and Cabo Verde. The table compares the real weather station's coordinates and height with the nearest grid-point location used for model validation, indicating the horizontal displacement (D_x) in kilometers and the vertical height difference (D_y) in meters between the two points.

Station					Model (nearest)				
**Archipelago**	**ID**	**Lat[°N]**	**Lon[°W]**	**Hgt**[m]	**Hgt**[m]	**Lat[°N]**	**Lon[°W]**	**D$_x$**[km]	**D$_y$**[m]
Azores	ADH	38.660	27.224	90	102	38.664	27.233	0.90	−12
Azores	HOR	38.530	28.629	60	16	38.522	28.630	0.89	44
Azores	PDA	37.744	25.708	77	122	37.734	25.716	1.31	−45
Madeira	FUN	32.649	16.893	25	249	32.627	16.913	3.08	−224
Canary Islands	FUE	28.444	13.863	25	58	28.445	13.878	1.67	−33
Canary Islands	LPA	27.922	15.389	24	46	27.910	15.396	1.56	−22
Canary Islands	VDE	27.819	17.889	32	89	27.830	17.885	1.28	−57
Canary Islands	ACE	28.952	13.600	14	43	28.951	13.605	0.52	−29
Canary Islands	SPC	28.633	17.755	33	154	28.632	17.763	0.93	−121
Canary Islands	TFN	28.477	16.329	632	683	28.472	16.337	1.04	−51
Canary Islands	TFS	28.047	16.561	64	33	28.044	16.549	1.32	31
Canary Islands	IZO	28.309	16.499	2,371	1,966	28.312	16.488	1.23	405
Canary Islands	SCT	28.463	16.255	35	66	28.472	16.246	1.46	−31
Cabo Verde	STG	14.935	23.485	95	88	14.949	23.500	2.24	7
Cabo Verde	SAL	16.732	22.935	55	34	16.731	22.923	1.28	21
Cabo Verde	SVT	16.833	25.055	20	99	16.847	25.048	1.72	−79

the present model's performance for the 1990–2019 period is demonstrably superior to that of a previous CMIP5 model, which was validated using a 1980–2009 baseline [2]. This confirms that the new model provides a more accurate representation of the historical climate for this specific region, which is a key advantage for future projections and increases confidence in the results for this archipelago.

The Q-Q plots (Figure S3 and Figure S4) offer further granular insight into the model's capabilities, revealing its excellent performance in accurately reproducing the full range and frequency distribution of observed conditions in the Azores and Madeira. In these archipelagos, the data points are tightly clustered around the ideal 1:1 line, signifying a strong agreement between simulated and observed values. However, minor systematic biases are discernible in these archipelagos; for instance, a consistent overestimation of the number of good tourism days for the HCIB60 and CCI05 indices is evident in Madeira. In the Canary Islands, the model's performance is found to be highly variable and dependent on the specific meteorological station. This is visually represented by a large spread of data points on the plots, indicating less consistent accuracy and significant biases for both sets of indices. Similarly, in Cabo Verde, the performance is less consistent, with the model struggling to accurately capture the full distribution for the TCI80, HCIU80, TCI60, and HCIU60 indices, while exhibiting strong positive and negative biases for the CCI07/CCI05 and HCIB80/HCIB60 indices, the nature of which varies depending on the station.

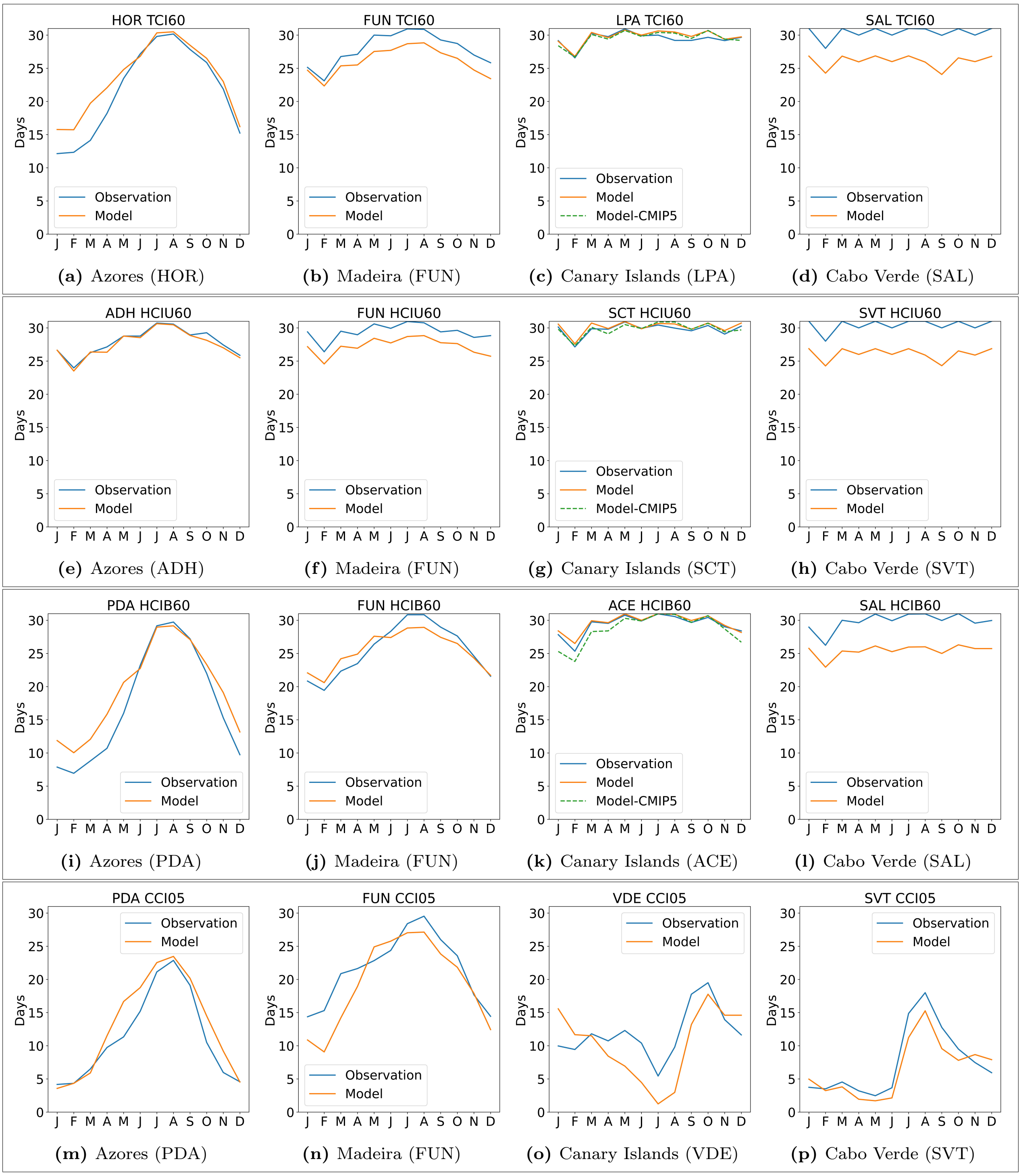

(a) Azores (HOR) (b) Madeira (FUN) (c) Canary Islands (LPA) (d) Cabo Verde (SAL)

(e) Azores (ADH) (f) Madeira (FUN) (g) Canary Islands (SCT) (h) Cabo Verde (SVT)

(i) Azores (PDA) (j) Madeira (FUN) (k) Canary Islands (ACE) (l) Cabo Verde (SAL)

(m) Azores (PDA) (n) Madeira (FUN) (o) Canary Islands (VDE) (p) Cabo Verde (SVT)

Figure S1. Validation of simulated indices. These plots compare the monthly average distributions of model simulations against observed data from ground-based meteorological stations for the 1990–2019 period. Stations were selected to represent the most relevant tourism types (general-purpose, urban, beach, and nature-based) across the Azores, Madeira, the Canary Islands, and Cabo Verde. Figures display the **TCI60**, **HCIU60**, **HCIB60**, and **CCI05** sub-indices, which denote the average number of days per month with an index value of at least 60 (TCI, HCIU, HCIB) or 5 (CCI) for good tourism conditions. For the TCI60, HCIU60, and HCIB60 plots in the Canary Islands, an additional line labeled **Model-CMIP5** is included; this line represents a comparative average from a former CMIP5 study based on a different reference period (1980–2009) [2].

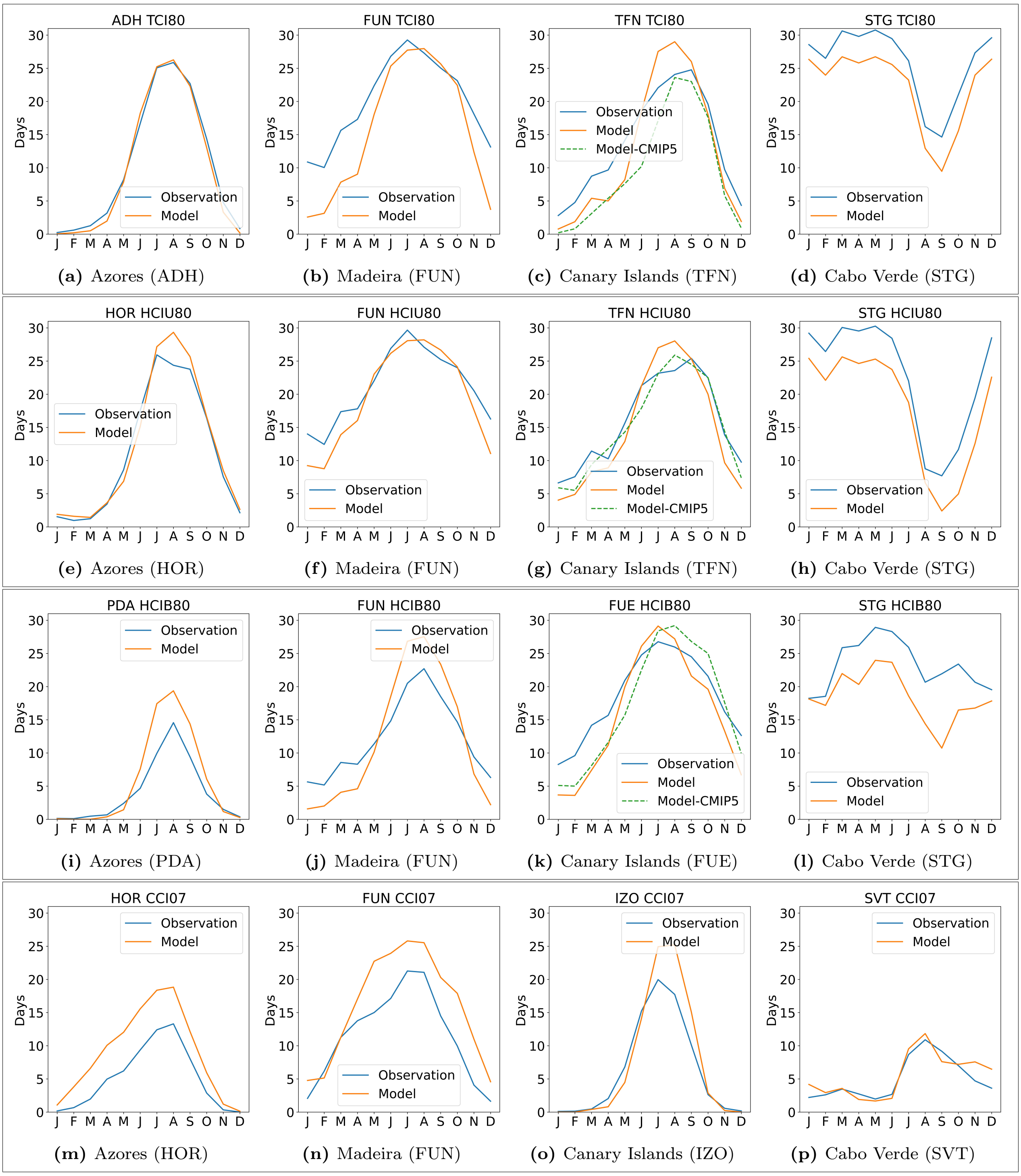

(a) Azores (ADH) **(b)** Madeira (FUN) **(c)** Canary Islands (TFN) **(d)** Cabo Verde (STG)

(e) Azores (HOR) **(f)** Madeira (FUN) **(g)** Canary Islands (TFN) **(h)** Cabo Verde (STG)

(i) Azores (PDA) **(j)** Madeira (FUN) **(k)** Canary Islands (FUE) **(l)** Cabo Verde (STG)

(m) Azores (HOR) **(n)** Madeira (FUN) **(o)** Canary Islands (IZO) **(p)** Cabo Verde (SVT)

Figure S2. Validation of simulated indices. These plots compare the monthly average distributions of model simulations against observed data from ground-based meteorological stations for the 1990–2019 period. Stations were selected to represent the most relevant tourism types (general-purpose, urban, beach, and nature-based) across the Azores, Madeira, the Canary Islands, and Cabo Verde. Figures display the **TCI80**, **HCIU80**, **HCIB80**, and **CCI07** sub-indices, which denote the average number of days per month with an index value of at least 80 (TCI, HCIU, HCIB) or 7 (CCI) for good tourism conditions. For the TCI60, HCIU60, and HCIB60 plots in the Canary Islands, an additional line labeled **Model-CMIP5** is included; this line represents a comparative average from a former CMIP5 study based on a different reference period (1980–2009) [2].

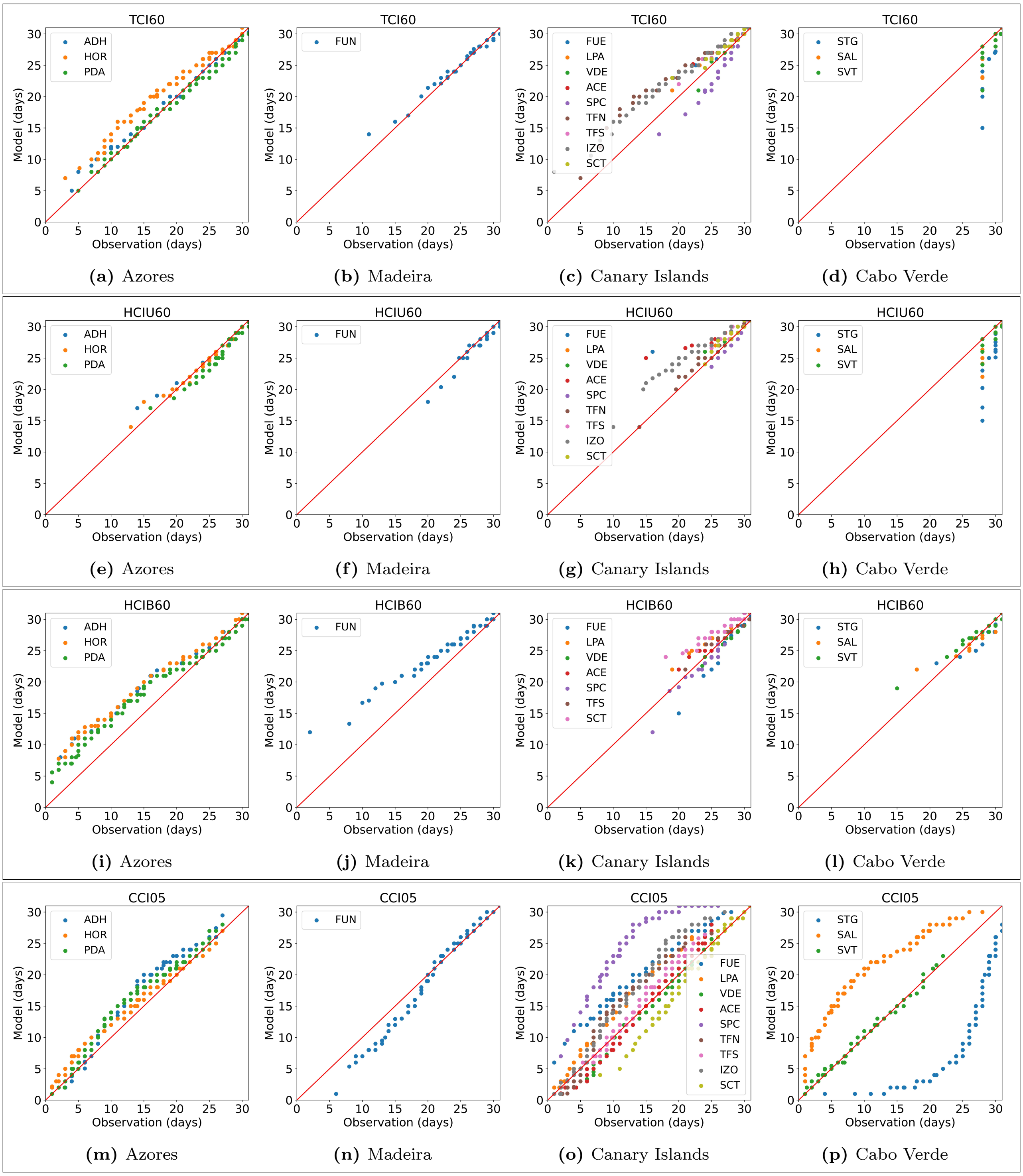

Figure S3. Validation of simulated indices (Q-Q plots). Q-Q plots compare the distributions of the **TCI60**, **HCIU60**, **HCIB60**, and **CCI05** sub-indices (number of days per month with index ≥ 80) from model simulations against observed data, using data from ground-based meteorological stations across the Macaronesian archipelagos (the Azores, Madeira, the Canary Islands, and Cabo Verde) during the 1990–2019 period. Note that non-coastal and elevated stations (IZO and TFN) in the Canary Islands have been excluded for sub-index HCIB60. The X- and Y-axes represent observed and simulated values (days), respectively, with the red line indicating ideal 1:1 agreement.

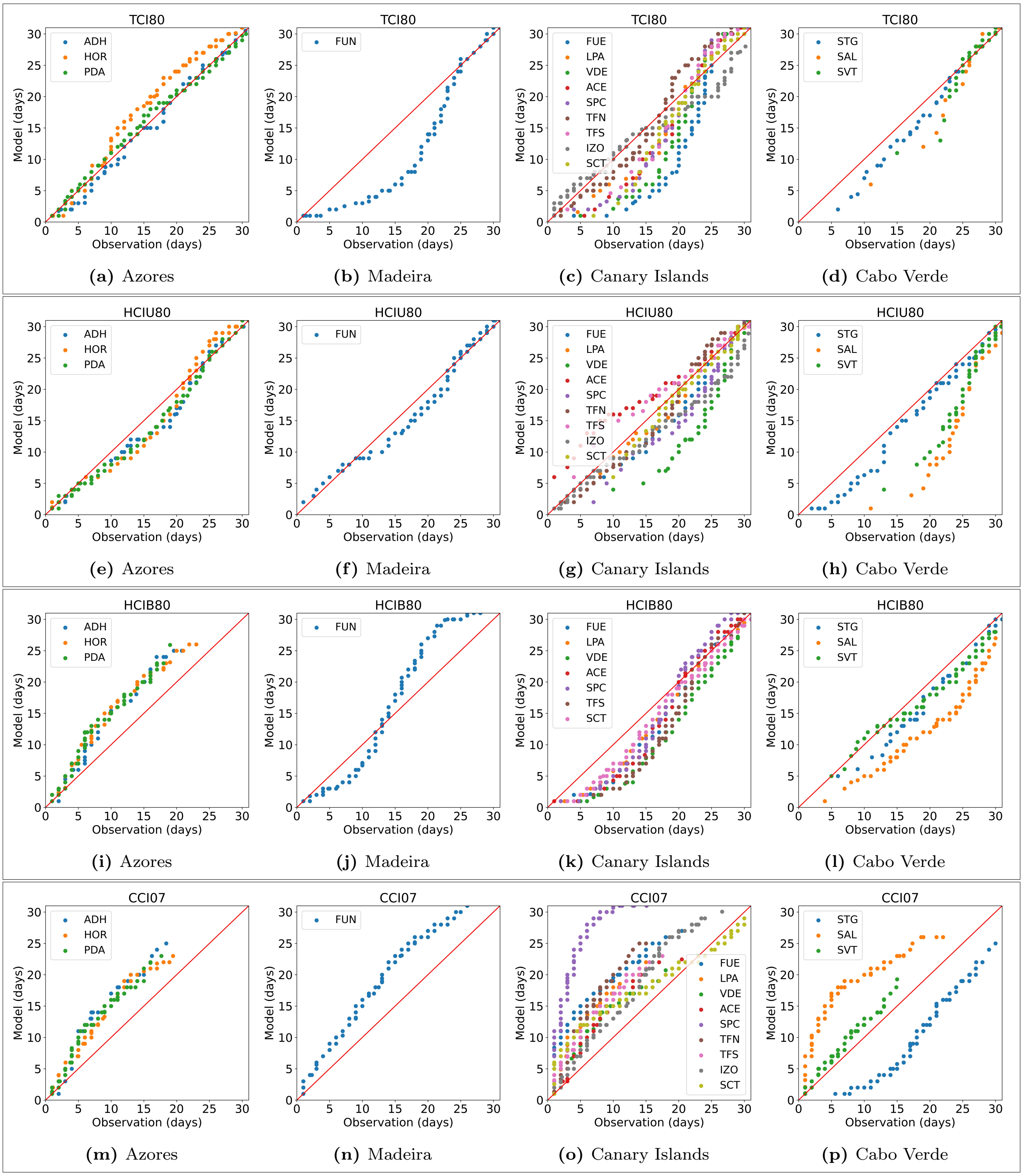

(a) Azores **(b)** Madeira **(c)** Canary Islands **(d)** Cabo Verde

(e) Azores **(f)** Madeira **(g)** Canary Islands **(h)** Cabo Verde

(i) Azores **(j)** Madeira **(k)** Canary Islands **(l)** Cabo Verde

(m) Azores **(n)** Madeira **(o)** Canary Islands **(p)** Cabo Verde

Figure S4. Validation of simulated indices (Q-Q plots). Q-Q plots compare the distributions of the **TCI80**, **HCIU80**, **HCIB80**, and **CCI07** sub-indices (number of days per month with index ≥ 80) from model simulations against observed data, using data from ground-based meteorological stations across the Macaronesian archipelagos (the Azores, Madeira, the Canary Islands, and Cabo Verde) during the 1990–2019 period. Note that non-coastal and elevated stations (IZO and TFN) in the Canary Islands have been excluded for sub-index HCIB80. The X- and Y-axes represent observed and simulated values (days), respectively, with the red line indicating ideal 1:1 agreement.

3 Projections of tourism climate indices

3.1 The Azores

In the recent past, the Azores had a short summer season for excellent tourism conditions. Projected changes indicate a varied outlook depending on the emissions scenario. The 2030–2059 SSP1-2.6 scenario shows a widespread decrease in most indices, particularly in summer. In contrast, the SSP5-8.5 scenario suggests a more favorable trend, particularly in 2070–2099, which could extend the tourism season into the off-season of spring and autumn. Table S2 summarizes these past conditions and future changes.

Table S2. Tourism climate indices for the Azores during the recent past baseline (1990–2019) and projected changes for the near- (2030–2059) and long-term future (2070–2099) under SSP1-2.6 and SSP5-8.5 scenarios.

Sub-index	Baseline conditions (1990–2019)	Changes (2030–2059 and 2070–2099)
TCI60 (Figure S5)	Good tourism conditions range from 0 to 15 days in winter, increasing to between 10 to 25 days in spring and autumn, and reaching a maximum of 20 to 31 days in summer.	2030–2059 SSP1-2.6 projects a decrease of 1 to 6 days, particularly in summer, while SSP5-8.5 projects a small increase of 1 to 2 days in the off-season. For 2070–2099, SSP1-2.6 projects an increase of 0 to 3 days, while SSP5-8.5 projects a widespread increase of up to 6 days.
TCI80 (Figure S6)	Excellent tourism conditions range from 0 to 5 days in winter, increasing to between 5 to 15 days in spring and autumn, and reaching a maximum of 15 to 25 days in summer.	2030–2059 SSP1-2.6 projects a decrease of 1 to 6 days, while SSP5-8.5 projects an increase of 1 to 3 days. This trend is more pronounced in 2070–2099, with SSP1-2.6 projecting increases of 1 to 4 days and SSP5-8.5 indicating increases of up to 9 days in spring and autumn.
HCIU60 (Figure S7)	Good urban tourism conditions range from 10 to 20 days in winter, increasing to between 15 to 25 days in spring and autumn, and reaching a maximum of 25 to 31 days in summer.	2030–2059 SSP1-2.6 projects decreases of up to 3 days. SSP5-8.5 projects a small decrease of up to 1 day. In 2070–2099, both SSP1-2.6 and SSP5-8.5 project increases of up to 2 days across all seasons.
HCIU80 (Figure S8)	Excellent urban tourism conditions range from 0 to 5 days in winter and spring, increasing to between 5 to 15 days in autumn, and reaching a maximum of 10 to 20 days in summer.	2030–2059 SSP1-2.6 projects a decrease of 1 to 3 days. SSP5-8.5 projects an increase of 1 to 4 days. In 2070–2099, SSP1-2.6 projects increases of 2 to 3 days, with SSP5-8.5 indicating broader increases of 2 to 6 days.
HCIB60 (Figure S9)	Good beach tourism conditions range from 0 to 15 days in winter and spring, increasing to between 10 to 20 days in autumn, and reaching a maximum of 20 to 25 days in summer.	2030–2059 SSP1-2.6 projects a decrease of 1 to 3 days, while SSP5-8.5 shows an increase of 1 to 2 days. In 2070–2099, SSP1-2.6 projects a general increase of 1 to 4 days, with SSP5-8.5 revealing increases of up to 5 days across all seasons.
HCIB80 (Figure S10)	Excellent beach tourism conditions range from 0 to 5 days in winter and spring, increasing to between 5 to 10 days in autumn, and reaching a maximum of 10 to 15 days in summer.	2030–2059 SSP1-2.6 projects a small widespread decrease of up to 1 day across all seasons, while SSP5-8.5 projects a general increase of up to 3 days in summer and autumn. In 2070–2099, SSP1-2.6 projects a general slight increase of 1 to 2 days, with SSP5-8.5 revealing increases of up to 7 days in summer and autumn.
CCI05 (Figure S11)	Good nature-based tourism conditions range from 0 to 10 days in winter and spring, increasing to between 5 to 15 days in autumn, and reaching a maximum of 15 to 20 days in summer.	2030–2059 SSP1-2.6 projects decreases of up to 5 days in summer, while SSP5-8.5 shows small widespread increases of up to 2 days. For 2070–2099, a similar general increase of up to 3 days is projected under both SSP scenarios, with a broader increase in spring of up to 5 days in the SSP5-8.5 scenario.
CCI07 (Figure S12)	Optimal nature-based tourism conditions range from 0 to 5 days in winter, increasing up to 10 days in spring and autumn, and reaching a maximum of 10 to 20 days in summer.	2030–2059 projects decreases in both SSP scenarios, with SSP1-2.6 indicating up to 4 days in summer. 2070–2099 shows a general increase of 1 to 3 days in spring under both SSP scenarios.

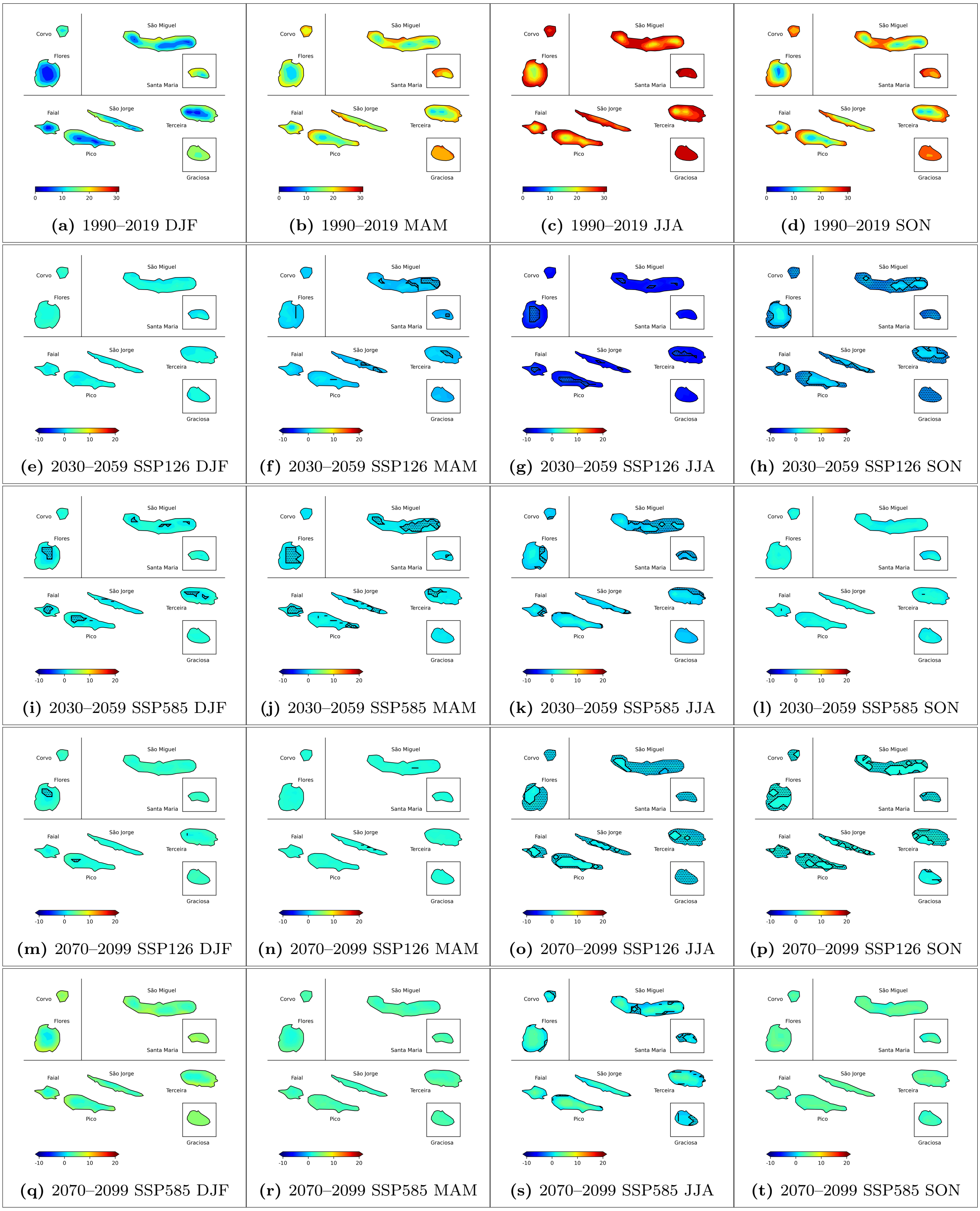

Figure S5. Simulated observations and projected changes in the **TCI60** sub-index for the **The Azores**. The TCI60 metric represents the number of good days for general-purpose tourism. The top row shows simulated observed values from 1990–2019 for all four seasons (DJF, MAM, JJA, and SON). The subsequent rows indicate projected changes (average seasonal differences in the monthly number of good days) for 2030–2059 and 2070–2099 under both the SSP1-2.6 and SSP5-8.5 scenarios, respectively, all relative to the 1990–2019 baseline. Areas with black dots indicate statistically non-significant changes, while non-hatched areas indicate statistically significant changes. These non-significant results reflect variability in the ensemble response rather than the absence of change.

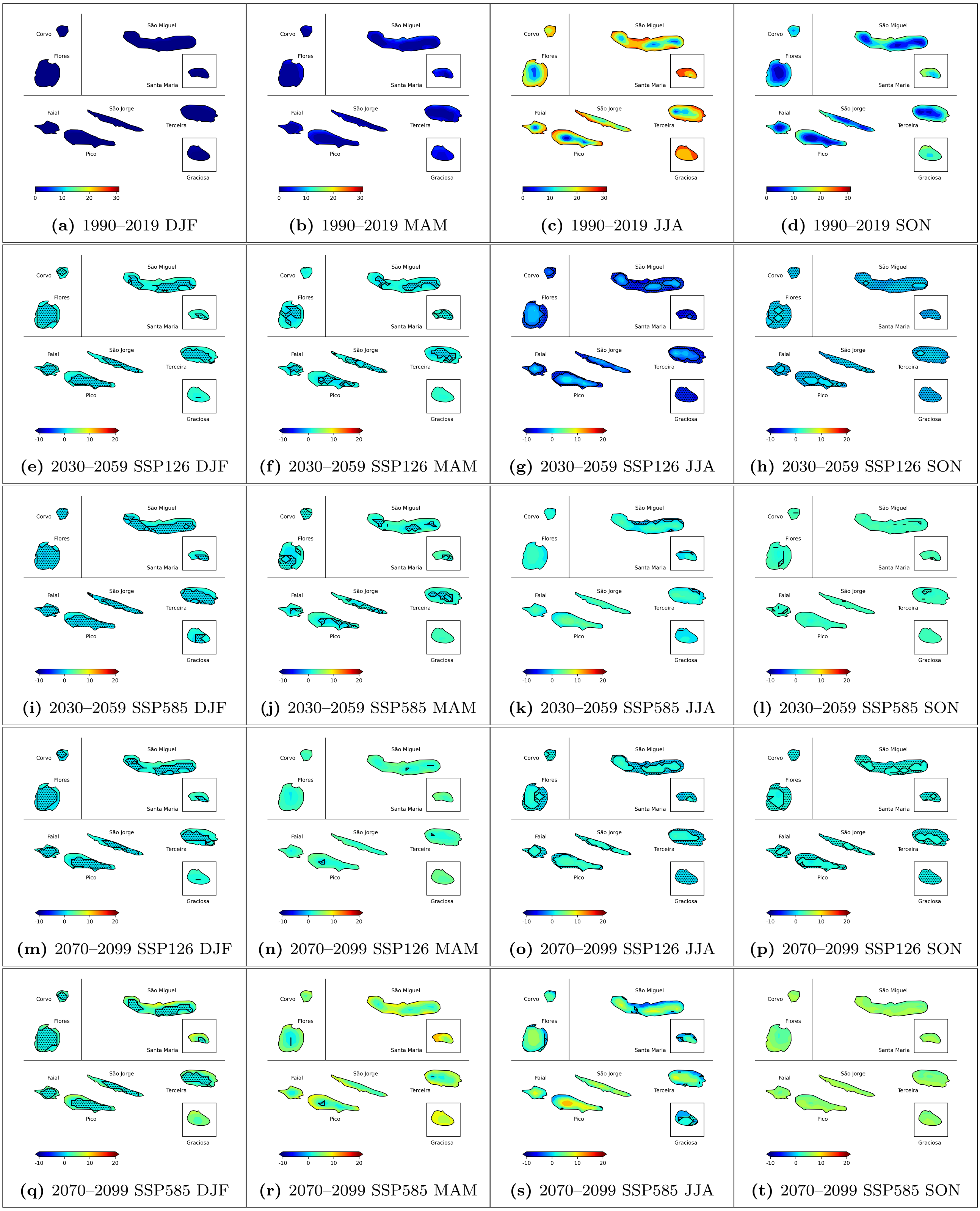

(a) 1990–2019 DJF (b) 1990–2019 MAM (c) 1990–2019 JJA (d) 1990–2019 SON

(e) 2030–2059 SSP126 DJF (f) 2030–2059 SSP126 MAM (g) 2030–2059 SSP126 JJA (h) 2030–2059 SSP126 SON

(i) 2030–2059 SSP585 DJF (j) 2030–2059 SSP585 MAM (k) 2030–2059 SSP585 JJA (l) 2030–2059 SSP585 SON

(m) 2070–2099 SSP126 DJF (n) 2070–2099 SSP126 MAM (o) 2070–2099 SSP126 JJA (p) 2070–2099 SSP126 SON

(q) 2070–2099 SSP585 DJF (r) 2070–2099 SSP585 MAM (s) 2070–2099 SSP585 JJA (t) 2070–2099 SSP585 SON

Figure S6. Simulated observations and projected changes in the **TCI80** sub-index for the **The Azores**. The TCI80 metric represents the number of excellent days for general-purpose tourism. The top row shows simulated observed values from 1990–2019 for all four seasons (DJF, MAM, JJA, and SON). The subsequent rows indicate projected changes (average seasonal differences in the monthly number of excellent days) for 2030–2059 and 2070–2099 under both the SSP1-2.6 and SSP5-8.5 scenarios, respectively, all relative to the 1990–2019 baseline. Areas with black dots indicate statistically non-significant changes, while non-hatched areas indicate statistically significant changes. These non-significant results reflect variability in the ensemble response rather than the absence of change.

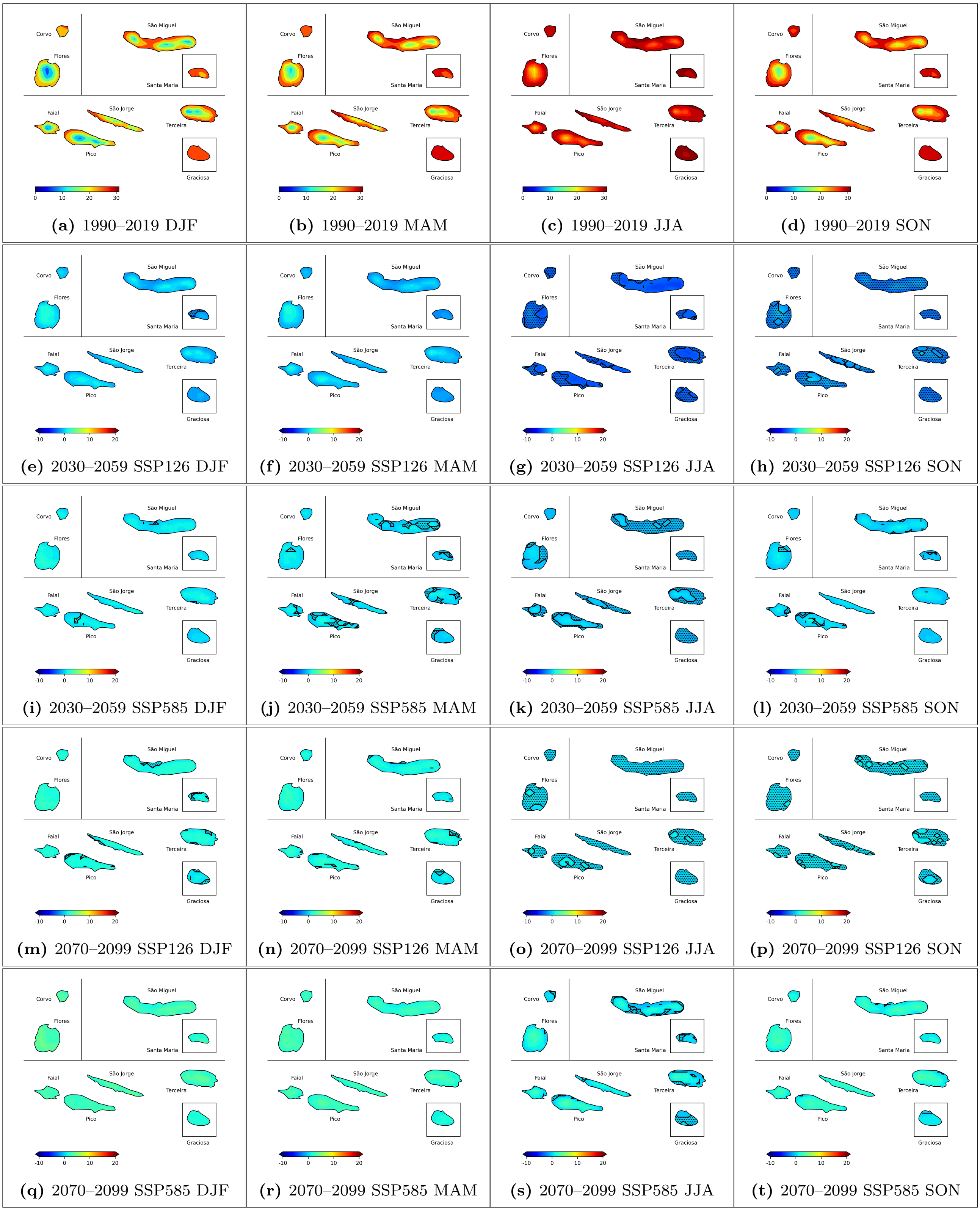

(a) 1990–2019 DJF **(b)** 1990–2019 MAM **(c)** 1990–2019 JJA **(d)** 1990–2019 SON

(e) 2030–2059 SSP126 DJF **(f)** 2030–2059 SSP126 MAM **(g)** 2030–2059 SSP126 JJA **(h)** 2030–2059 SSP126 SON

(i) 2030–2059 SSP585 DJF **(j)** 2030–2059 SSP585 MAM **(k)** 2030–2059 SSP585 JJA **(l)** 2030–2059 SSP585 SON

(m) 2070–2099 SSP126 DJF **(n)** 2070–2099 SSP126 MAM **(o)** 2070–2099 SSP126 JJA **(p)** 2070–2099 SSP126 SON

(q) 2070–2099 SSP585 DJF **(r)** 2070–2099 SSP585 MAM **(s)** 2070–2099 SSP585 JJA **(t)** 2070–2099 SSP585 SON

Figure S7. Simulated observations and projected changes in the **HCIU60** sub-index for the **The Azores**. The HCIU60 metric represents the number of good days for urban tourism. The top row shows simulated observed values from 1990–2019 for all four seasons (DJF, MAM, JJA, and SON). The subsequent rows indicate projected changes (average seasonal differences in the monthly number of good days) for 2030–2059 and 2070–2099 under both the SSP1-2.6 and SSP5-8.5 scenarios, respectively, all relative to the 1990–2019 baseline. Areas with black dots indicate statistically non-significant changes, while non-hatched areas indicate statistically significant changes. These non-significant results reflect variability in the ensemble response rather than the absence of change.

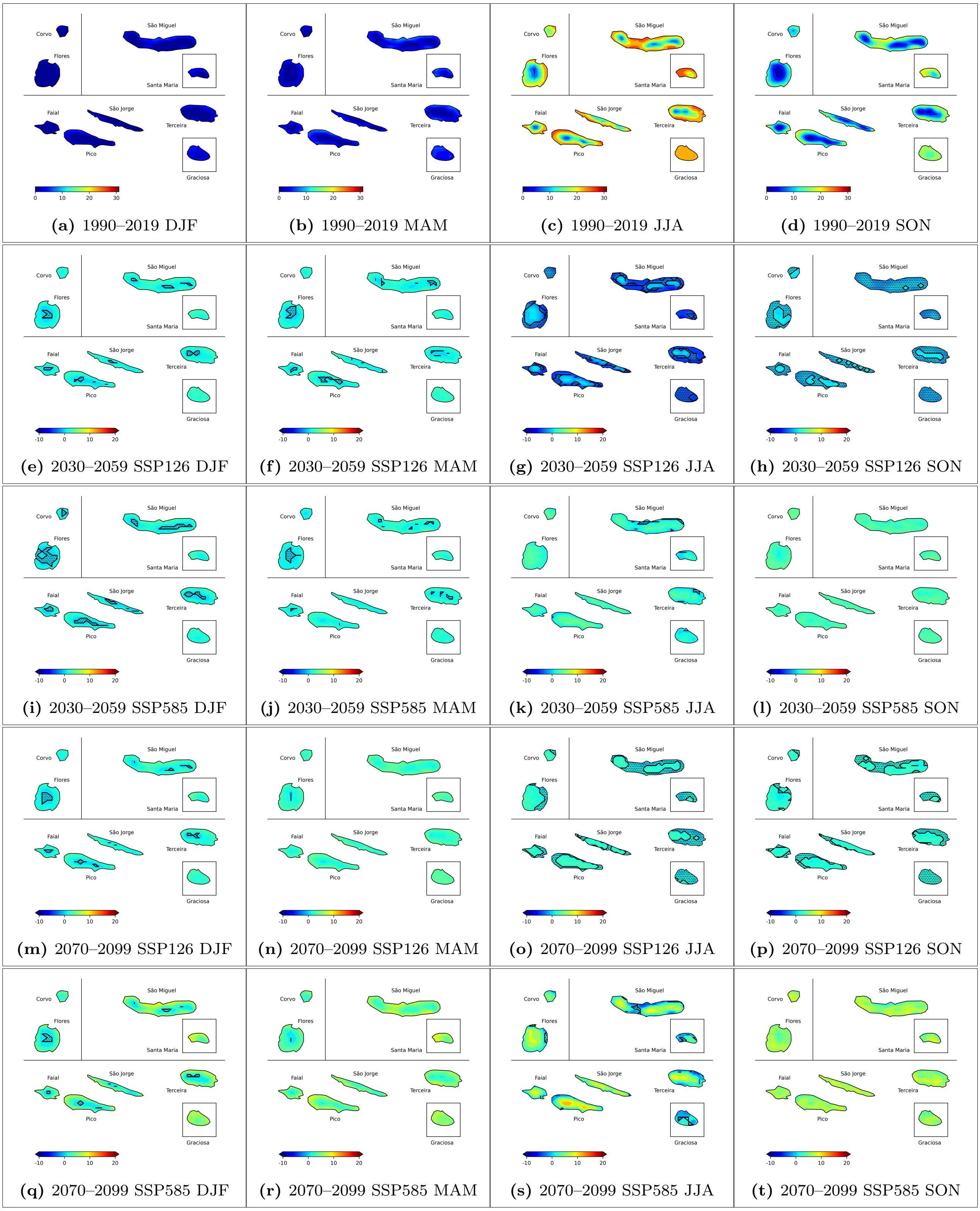

(a) 1990–2019 DJF (b) 1990–2019 MAM (c) 1990–2019 JJA (d) 1990–2019 SON

(e) 2030–2059 SSP126 DJF (f) 2030–2059 SSP126 MAM (g) 2030–2059 SSP126 JJA (h) 2030–2059 SSP126 SON

(i) 2030–2059 SSP585 DJF (j) 2030–2059 SSP585 MAM (k) 2030–2059 SSP585 JJA (l) 2030–2059 SSP585 SON

(m) 2070–2099 SSP126 DJF (n) 2070–2099 SSP126 MAM (o) 2070–2099 SSP126 JJA (p) 2070–2099 SSP126 SON

(q) 2070–2099 SSP585 DJF (r) 2070–2099 SSP585 MAM (s) 2070–2099 SSP585 JJA (t) 2070–2099 SSP585 SON

Figure S8. Simulated observations and projected changes in the **HCIU80** sub-index for the **The Azores**. The HCIU80 metric represents the number of excellent days for urban tourism. The top row shows simulated observed values from 1990–2019 for all four seasons (DJF, MAM, JJA, and SON). The subsequent rows indicate projected changes (average seasonal differences in the monthly number of excellent days) for 2030–2059 and 2070–2099 under both the SSP1-2.6 and SSP5-8.5 scenarios, respectively, all relative to the 1990–2019 baseline. Areas with black dots indicate statistically non-significant changes, while non-hatched areas indicate statistically significant changes. These non-significant results reflect variability in the ensemble response rather than the absence of change.

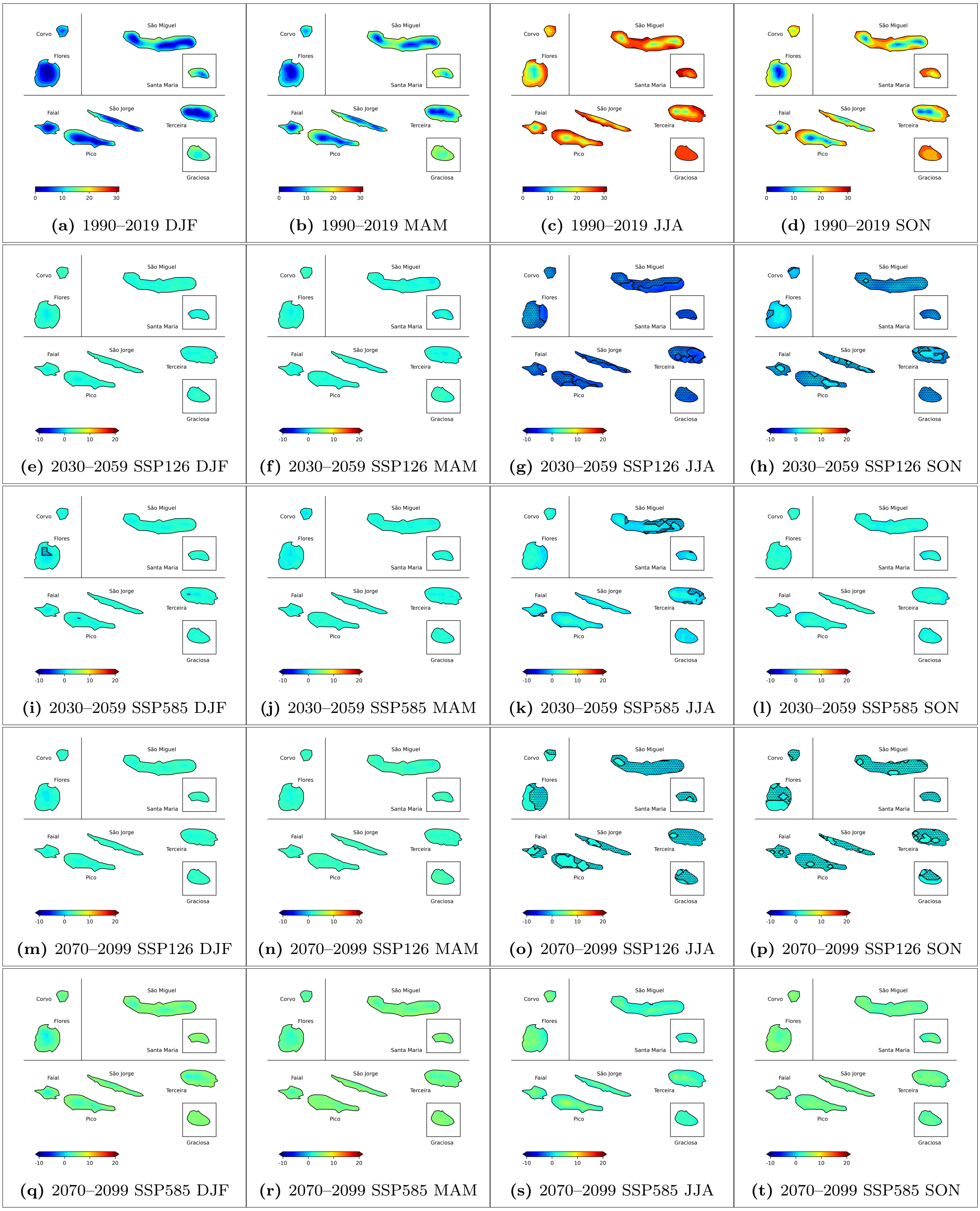

Figure S9. Simulated observations and projected changes in the **HCIB60** sub-index for the **The Azores**. The HCIB60 metric represents the number of good days for beach tourism. The top row shows simulated observed values from 1990–2019 for all four seasons (DJF, MAM, JJA, and SON). The subsequent rows indicate projected changes (average seasonal differences in the monthly number of good days) for 2030–2059 and 2070–2099 under both the SSP1-2.6 and SSP5-8.5 scenarios, respectively, all relative to the 1990–2019 baseline. Areas with black dots indicate statistically non-significant changes, while non-hatched areas indicate statistically significant changes. These non-significant results reflect variability in the ensemble response rather than the absence of change.

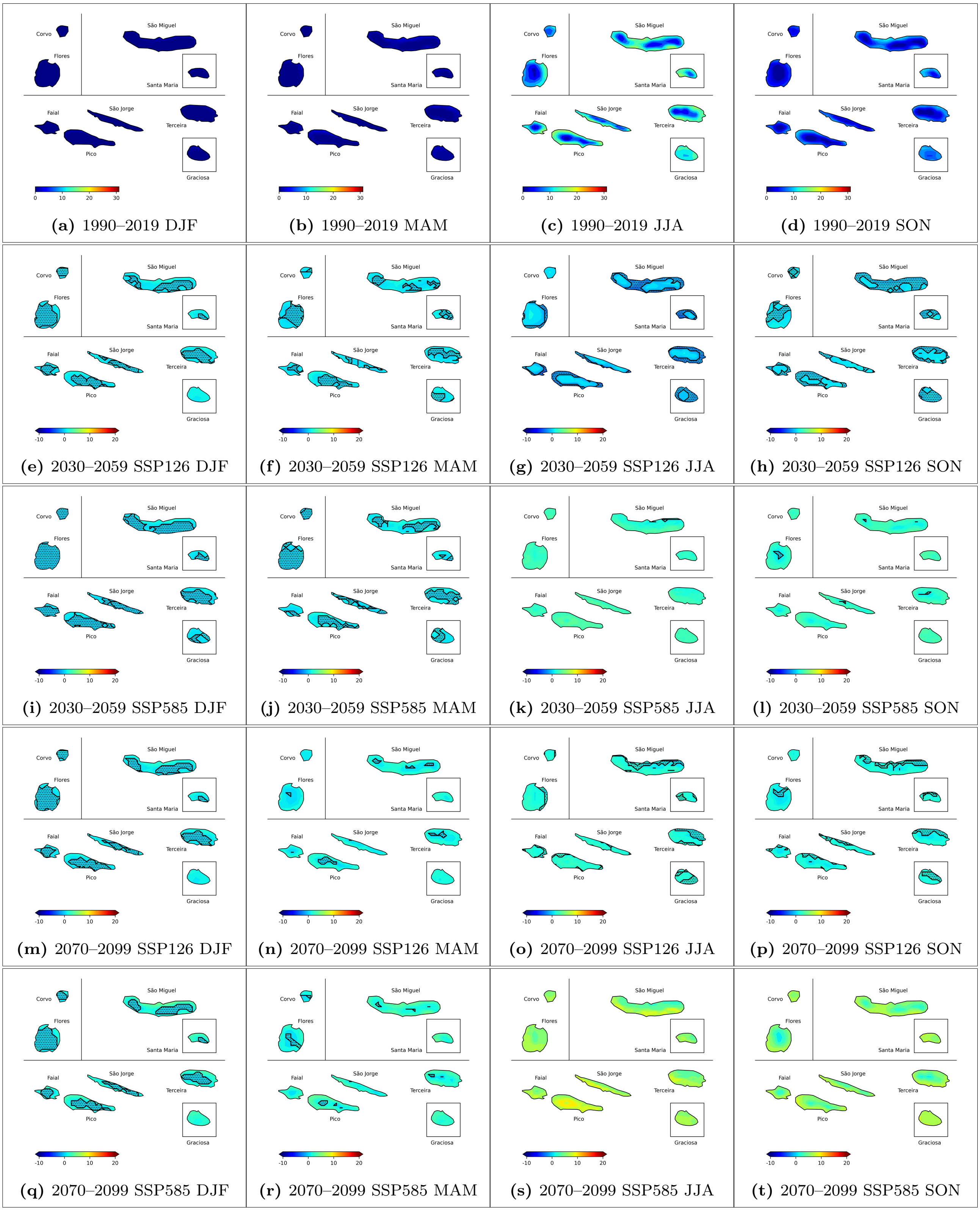

(a) 1990–2019 DJF
(b) 1990–2019 MAM
(c) 1990–2019 JJA
(d) 1990–2019 SON
(e) 2030–2059 SSP126 DJF
(f) 2030–2059 SSP126 MAM
(g) 2030–2059 SSP126 JJA
(h) 2030–2059 SSP126 SON
(i) 2030–2059 SSP585 DJF
(j) 2030–2059 SSP585 MAM
(k) 2030–2059 SSP585 JJA
(l) 2030–2059 SSP585 SON
(m) 2070–2099 SSP126 DJF
(n) 2070–2099 SSP126 MAM
(o) 2070–2099 SSP126 JJA
(p) 2070–2099 SSP126 SON
(q) 2070–2099 SSP585 DJF
(r) 2070–2099 SSP585 MAM
(s) 2070–2099 SSP585 JJA
(t) 2070–2099 SSP585 SON

Figure S10. Simulated observations and projected changes in the **HCIB80** sub-index for the **The Azores**. The HCIB80 metric represents the number of excellent days for beach tourism. The top row shows simulated observed values from 1990–2019 for all four seasons (DJF, MAM, JJA, and SON). The subsequent rows indicate projected changes (average seasonal differences in the monthly number of excellent days) for 2030–2059 and 2070–2099 under both the SSP1-2.6 and SSP5-8.5 scenarios, respectively, all relative to the 1990–2019 baseline. Areas with black dots indicate statistically non-significant changes, while non-hatched areas indicate statistically significant changes. These non-significant results reflect variability in the ensemble response rather than the absence of change.

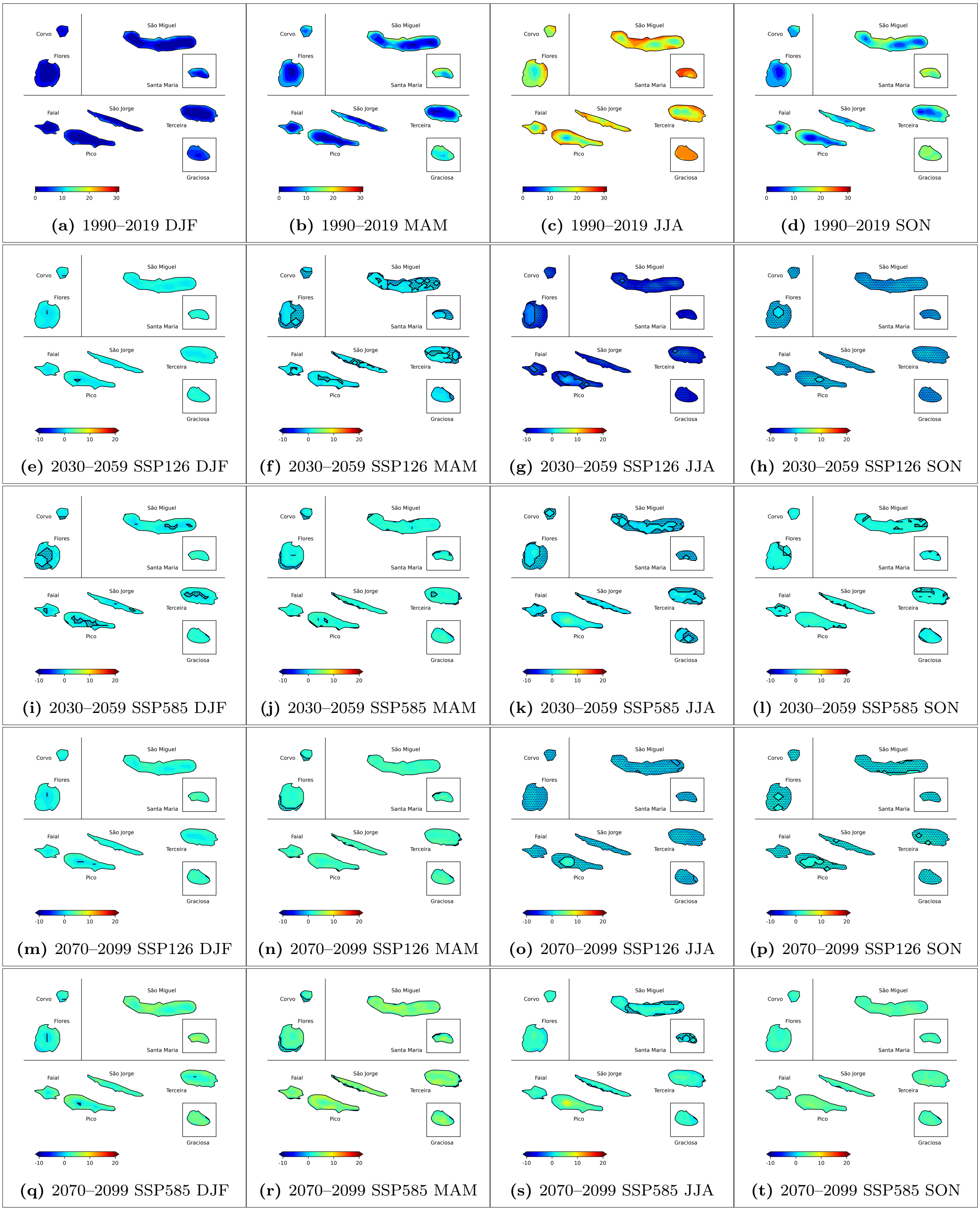

Figure S11. Simulated observations and projected changes in the **CCI05** sub-index for the **The Azores**. The CCI05 metric represents the number of good days for nature-based tourism. The top row shows simulated observed values from 1990–2019 for all four seasons (DJF, MAM, JJA, and SON). The subsequent rows indicate projected changes (average seasonal differences in the monthly number of good days) for 2030–2059 and 2070–2099 under both the SSP1-2.6 and SSP5-8.5 scenarios, respectively, all relative to the 1990–2019 baseline. Areas with black dots indicate statistically non-significant changes, while non-hatched areas indicate statistically significant changes. These non-significant results reflect variability in the ensemble response rather than the absence of change.

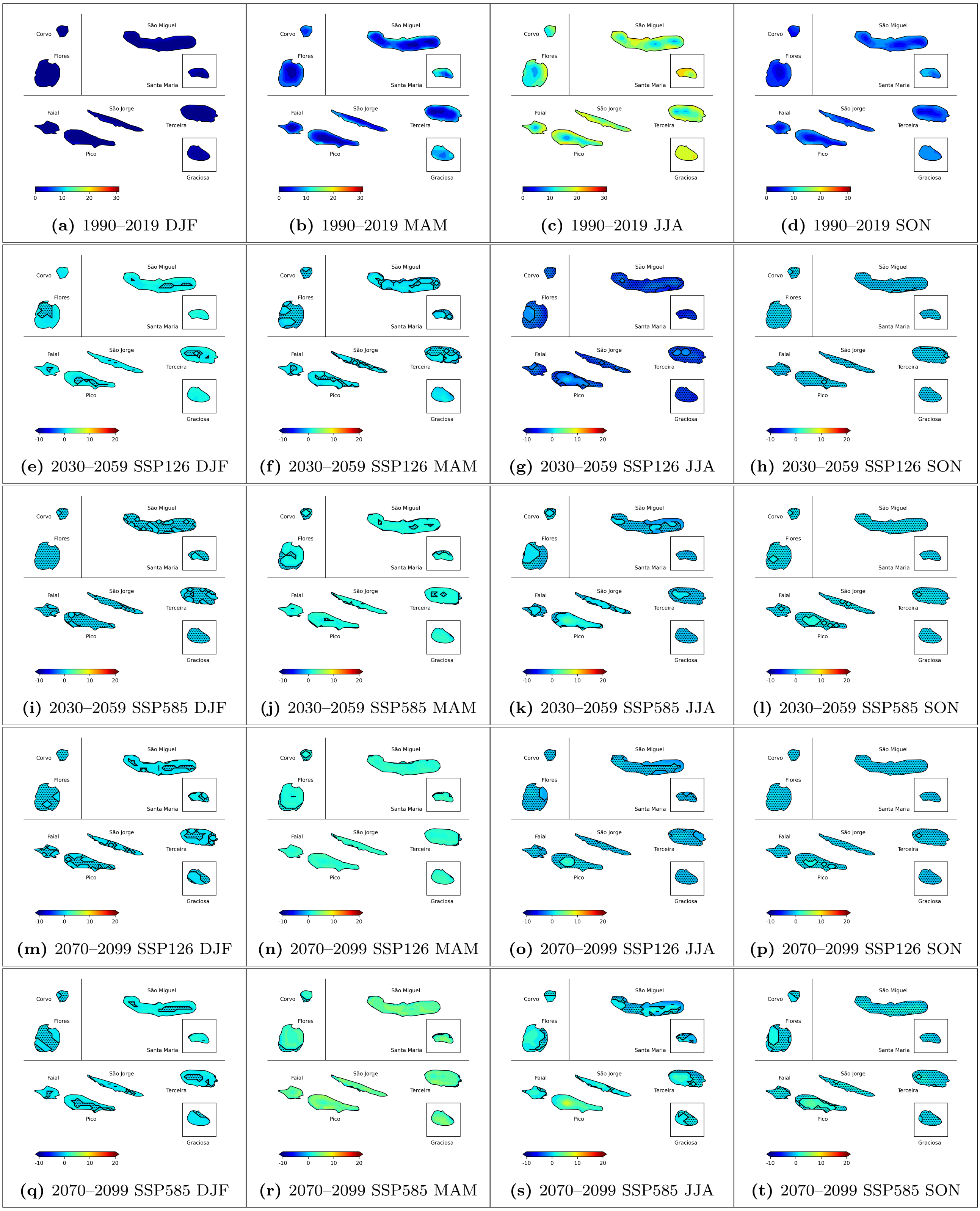

(a) 1990–2019 DJF **(b)** 1990–2019 MAM **(c)** 1990–2019 JJA **(d)** 1990–2019 SON

(e) 2030–2059 SSP126 DJF **(f)** 2030–2059 SSP126 MAM **(g)** 2030–2059 SSP126 JJA **(h)** 2030–2059 SSP126 SON

(i) 2030–2059 SSP585 DJF **(j)** 2030–2059 SSP585 MAM **(k)** 2030–2059 SSP585 JJA **(l)** 2030–2059 SSP585 SON

(m) 2070–2099 SSP126 DJF **(n)** 2070–2099 SSP126 MAM **(o)** 2070–2099 SSP126 JJA **(p)** 2070–2099 SSP126 SON

(q) 2070–2099 SSP585 DJF **(r)** 2070–2099 SSP585 MAM **(s)** 2070–2099 SSP585 JJA **(t)** 2070–2099 SSP585 SON

Figure S12. Simulated observations and projected changes in the **CCI07** sub-index for the **The Azores**. The CCI07 metric represents the number of optimal days for nature-based tourism. The top row shows simulated observed values from 1990–2019 for all four seasons (DJF, MAM, JJA, and SON). The subsequent rows indicate projected changes (differences in the average number of optimal days per month) for 2030–2059 and 2070–2099 under both the SSP1-2.6 and SSP5-8.5 scenarios, respectively, all relative to the 1990–2019 baseline. Areas with black dots indicate statistically non-significant changes, while non-hatched areas indicate statistically significant changes. These non-significant results reflect variability in the ensemble response rather than the absence of change.

3.2 Madeira

Madeira's climate projections show a generally favorable outlook for tourism. The high-emissions SSP5-8.5 scenario consistently projects a widespread increase in good and excellent tourism days, particularly in the shoulder seasons of winter and spring. In contrast, the low-emissions SSP1-2.6 scenario in 2030–2059 shows a decrease in good conditions before improving in 2070–2099. While summer remains highly favorable, the warming climate is expected to extend the tourism year by making other seasons more attractive for visitors. Table S3 summarizes these past conditions and future changes.

Table S3. Tourism climate indices for Madeira during the recent past baseline (1990–2019) and projected changes for the near- (2030–2059) and long-term future (2070–2099) under SSP1-2.6 and SSP5-8.5 scenarios.

**Sub-index**	**Baseline conditions (1990–2019)**	**Changes (2030–2059 and 2070–2099)**
TCI60 (Figure S13)	Good tourism conditions range from 15 to 20 days in winter, increasing to between 15 to 25 days in spring and autumn, and reaching a maximum of 25 to 31 days in summer.	Under SSP1-2.6, a decrease of 1 to 3 days is projected for 2030–2059, before increasing by 2 to 4 days in 2070–2099. In contrast, SSP5-8.5 projects a consistent increase, with a rise of 1 to 3 days in 2030–2059, and a widespread increase of 2 to 5 days by 2070–2099.
TCI80 (Figure S14)	Excellent tourism conditions range from 0 to 5 days in winter and spring, increasing to between 5 to 15 days in autumn, and reaching a maximum of 15 to 25 days in summer.	SSP1-2.6 in 2030–2059 projects an increase of up to 2 days, rising to 3 to 5 days in 2070–2099. SSP5-8.5 projects a much larger increase of 2 to 14 days in 2070–2099.
HCIU60 (Figure S15)	Good urban tourism conditions range from 20 to 25 days in winter, increasing to between 20 to 31 days in spring and autumn, and reaching a maximum of 25 to 31 days in summer.	SSP1-2.6 projects a 2030–2059 decrease of up to 3 days, which then reverses to an increase of 2 to 3 days in 2070–2099. In contrast, SSP5-8.5 consistently projects an increase of 1 to 3 days across both periods.
HCIU80 (Figure S16)	Excellent urban tourism conditions range from 0 to 10 days in winter and spring, increasing to between 5 to 15 days in autumn, and reaching a maximum of 15 to 31 days in summer.	2030–2059 SSP5-8.5 and 2070–2099 SSP1-2.6 projections reveal increases of 3 to 4 days, while SSP5-8.5 in 2070–2099 projects stronger increases of 5 to 9 days.
HCIB60 (Figure S17)	Good beach tourism conditions range from 5 to 15 days in winter, increasing to between 10 to 20 days in spring and autumn, and reaching a maximum of 25 to 31 days in summer.	SSP1-2.6 in 2030–2059 projects a decrease of up to 1 day, followed by an increase of 2 to 3 days in 2070–2099. SSP5-8.5 consistently projects an increase, rising by 2 days in 2030–2059 and up to 4 days in 2070–2099.
HCIB80 (Figure S18)	Excellent beach tourism conditions range from 0 to 5 days in winter and spring, increasing to between 5 to 10 days in autumn, and reaching a maximum of 10 to 10 days in summer.	2030–2059 SSP5-8.5 and 2070–2099 SSP1-2.6 projections indicate an increase of up to 3 days in summer and autumn, while 2070–2099 SSP5-8.5 projects a broader increase of up to 10 days.
CCI05 (Figure S19)	Good nature-based tourism conditions range from 0 to 10 days in winter and spring, increasing to between 10 to 15 days in autumn, and reaching a maximum of 15 to 25 days in summer.	2030–2059 projections project a decrease of up to 1 day under SSP1-2.6, and increases of up to 3 days for SSP5-8.5. In 2070–2099, both scenarios project increases of between 1 to 3 days for SSP1-2.6 and up to 10 days for SSP5-8.5.
CCI07 (Figure S20)	Optimal nature-based tourism conditions range from 0 to 10 days in winter and spring, increasing to between 5 to 10 days in autumn, and reaching a maximum of 15 to 25 days in summer.	2030–2059 projects a decrease of up to 1 day for SSP1-2.6, which then reverses to an increase of up to 3 days in SSP5-8.5. In 2070–2099, SSP1-2.6 projects similar increases of up to 3 days, while SSP5-8.5 extends to up to 5 days.

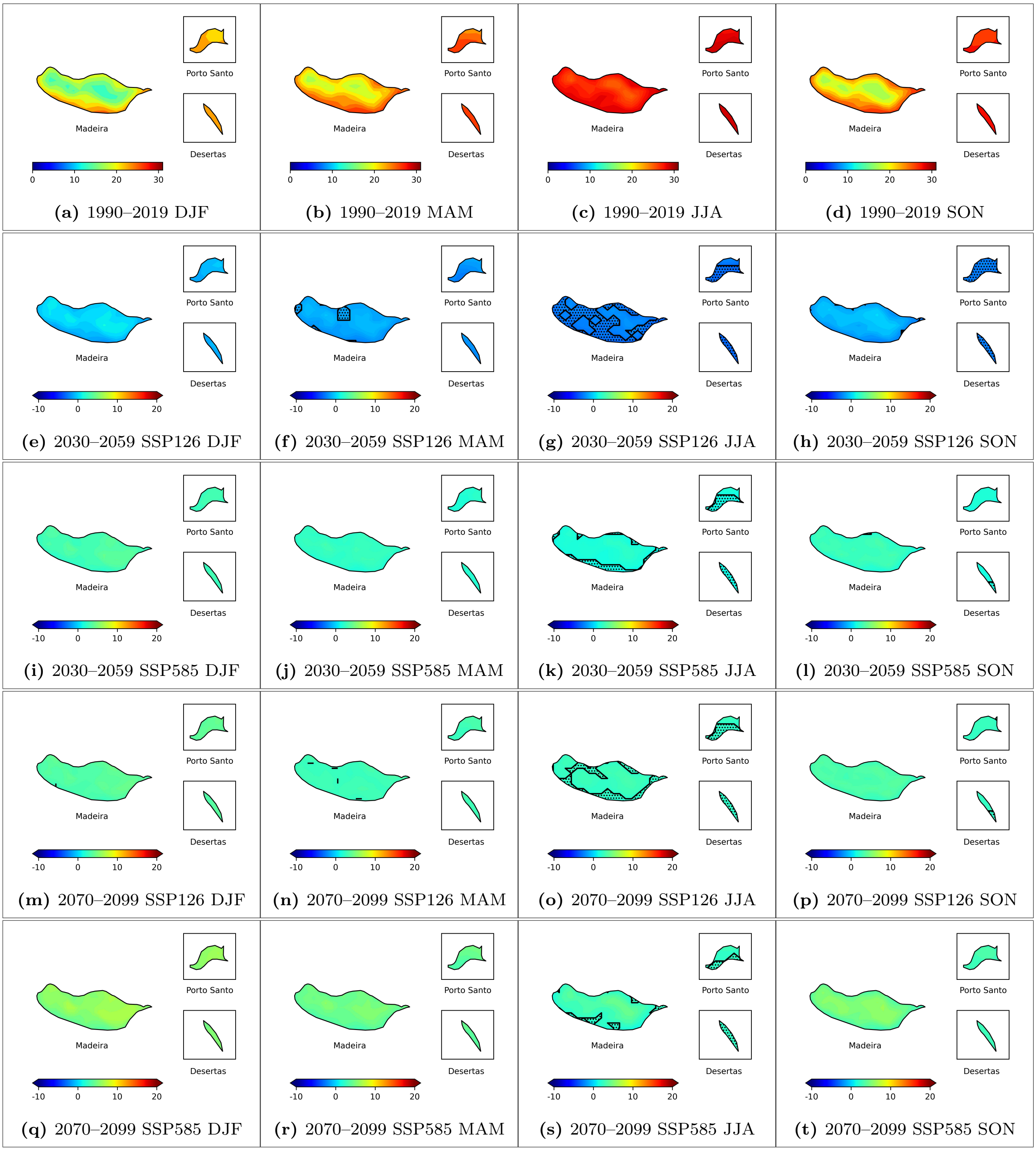

Figure S13. Simulated observations and projected changes in the **TCI60** sub-index for **Madeira**. The TCI60 metric represents the number of good days for general-purpose tourism. The top row indicates simulated observed values from 1990–2019 for all four seasons (DJF, MAM, JJA, and SON). The subsequent rows indicate projected changes (average seasonal differences in the monthly number of good days) for 2030–2059 and 2070–2099 under both the SSP1-2.6 and SSP5-8.5 scenarios, respectively, all relative to the 1990–2019 baseline. Areas with black dots indicate statistically non-significant changes, while non-hatched areas indicate statistically significant changes. These non-significant results reflect variability in the ensemble response rather than the absence of change.

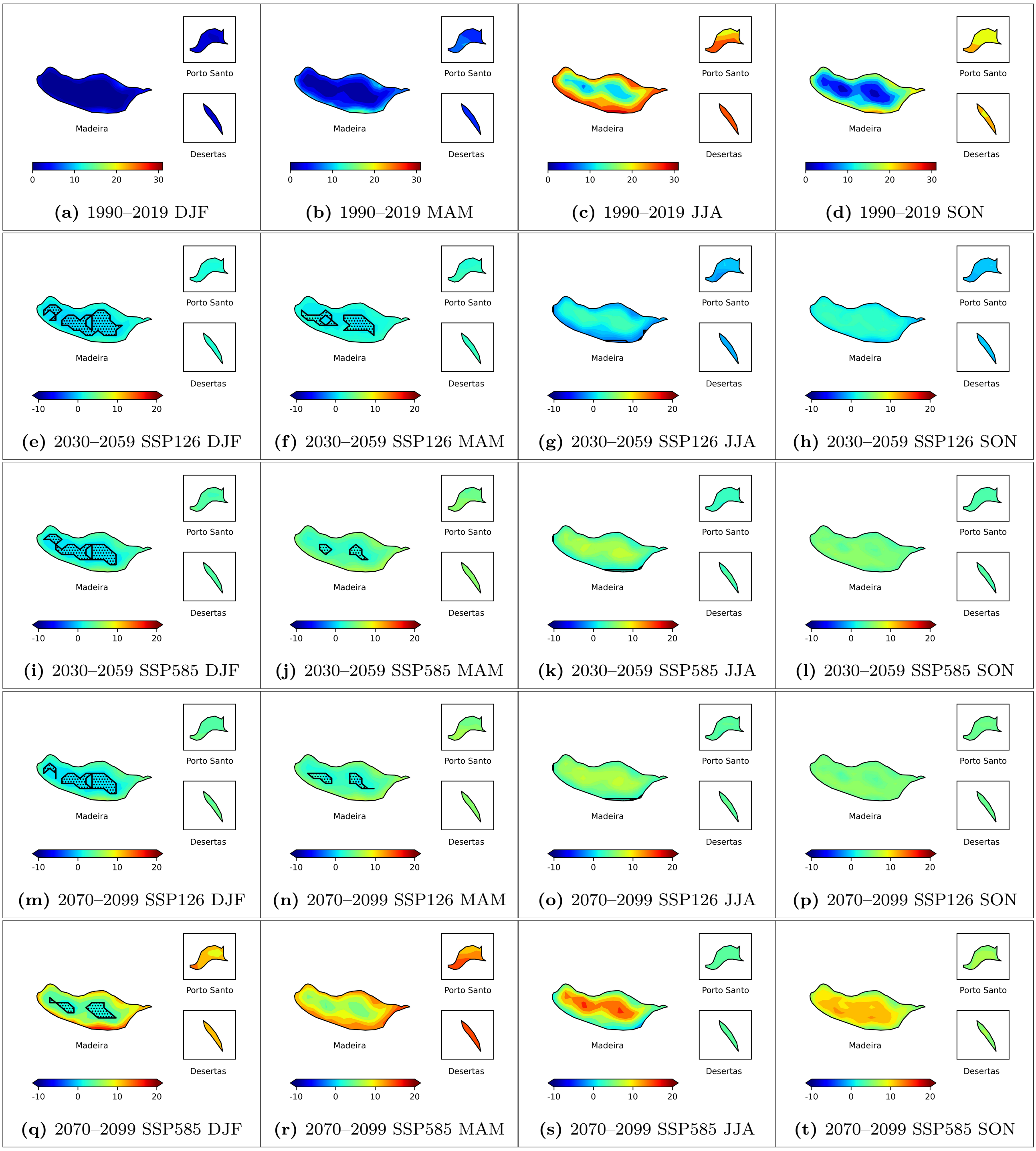

(a) 1990–2019 DJF **(b)** 1990–2019 MAM **(c)** 1990–2019 JJA **(d)** 1990–2019 SON

(e) 2030–2059 SSP126 DJF **(f)** 2030–2059 SSP126 MAM **(g)** 2030–2059 SSP126 JJA **(h)** 2030–2059 SSP126 SON

(i) 2030–2059 SSP585 DJF **(j)** 2030–2059 SSP585 MAM **(k)** 2030–2059 SSP585 JJA **(l)** 2030–2059 SSP585 SON

(m) 2070–2099 SSP126 DJF **(n)** 2070–2099 SSP126 MAM **(o)** 2070–2099 SSP126 JJA **(p)** 2070–2099 SSP126 SON

(q) 2070–2099 SSP585 DJF **(r)** 2070–2099 SSP585 MAM **(s)** 2070–2099 SSP585 JJA **(t)** 2070–2099 SSP585 SON

Figure S14. Simulated observations and projected changes in the **TCI80** sub-index for **Madeira**. The TCI80 metric represents the number of excellent days for general-purpose tourism. The top row shows simulated observed values from 1990–2019 for all four seasons (DJF, MAM, JJA, and SON). The subsequent rows indicate projected changes (average seasonal differences in the monthly number of excellent days) for 2030–2059 and 2070–2099 under both the SSP1-2.6 and SSP5-8.5 scenarios, respectively, all relative to the 1990–2019 baseline. Areas with black dots indicate statistically non-significant changes, while non-hatched areas indicate statistically significant changes. These non-significant results reflect variability in the ensemble response rather than the absence of change.

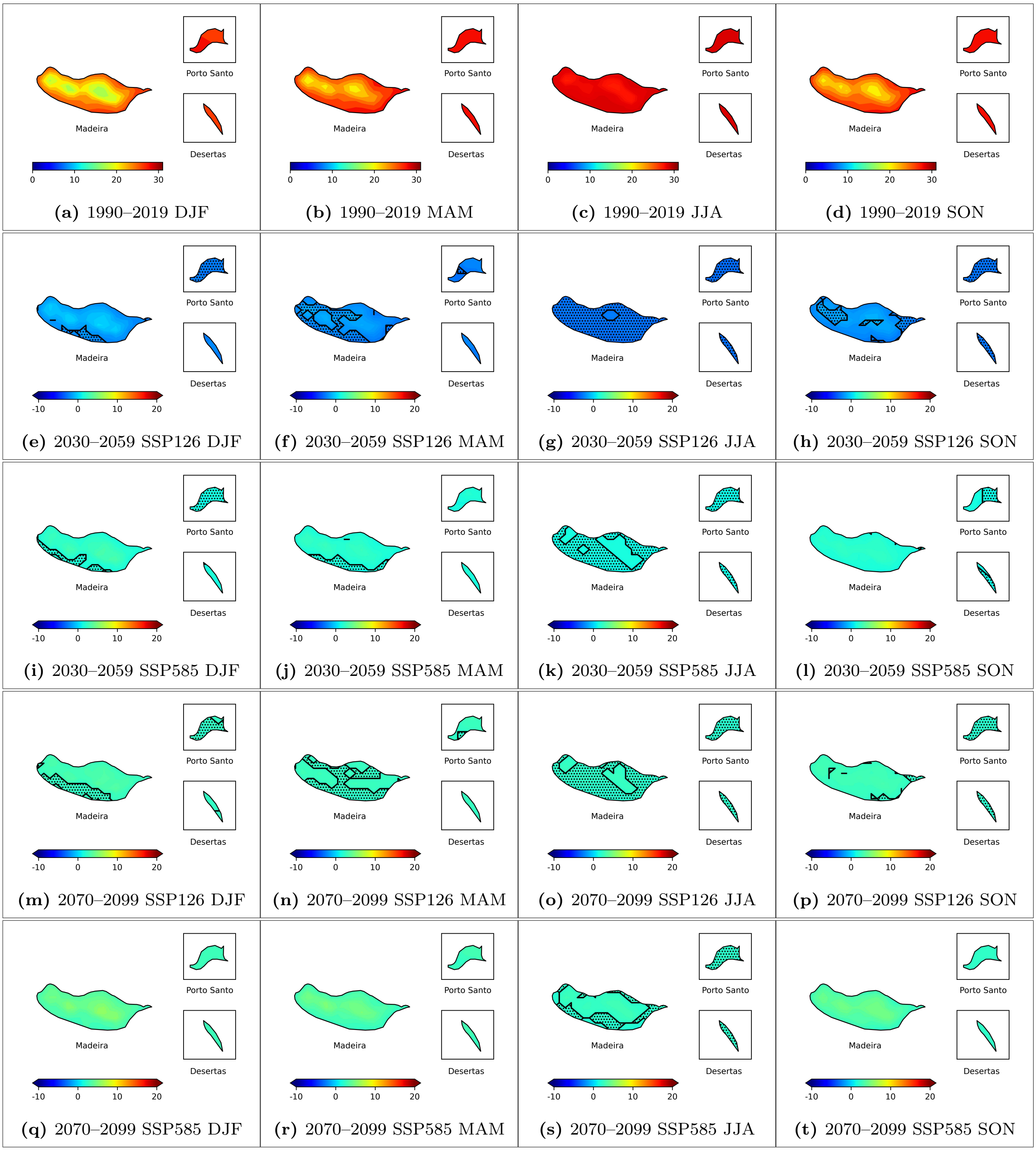

Figure S15. Simulated observations and projected changes in the **HCIU60** sub-index for **Madeira**. The HCIU60 metric represents the number of good days for urban tourism. The top row shows simulated observed values from 1990–2019 for all four seasons (DJF, MAM, JJA, and SON). The subsequent rows indicate projected changes (average seasonal differences in the monthly number of good days) for 2030–2059 and 2070–2099 under both the SSP1-2.6 and SSP5-8.5 scenarios, respectively, all relative to the 1990–2019 baseline. Areas with black dots indicate statistically non-significant changes, while non-hatched areas indicate statistically significant changes. These non-significant results reflect variability in the ensemble response rather than the absence of change.

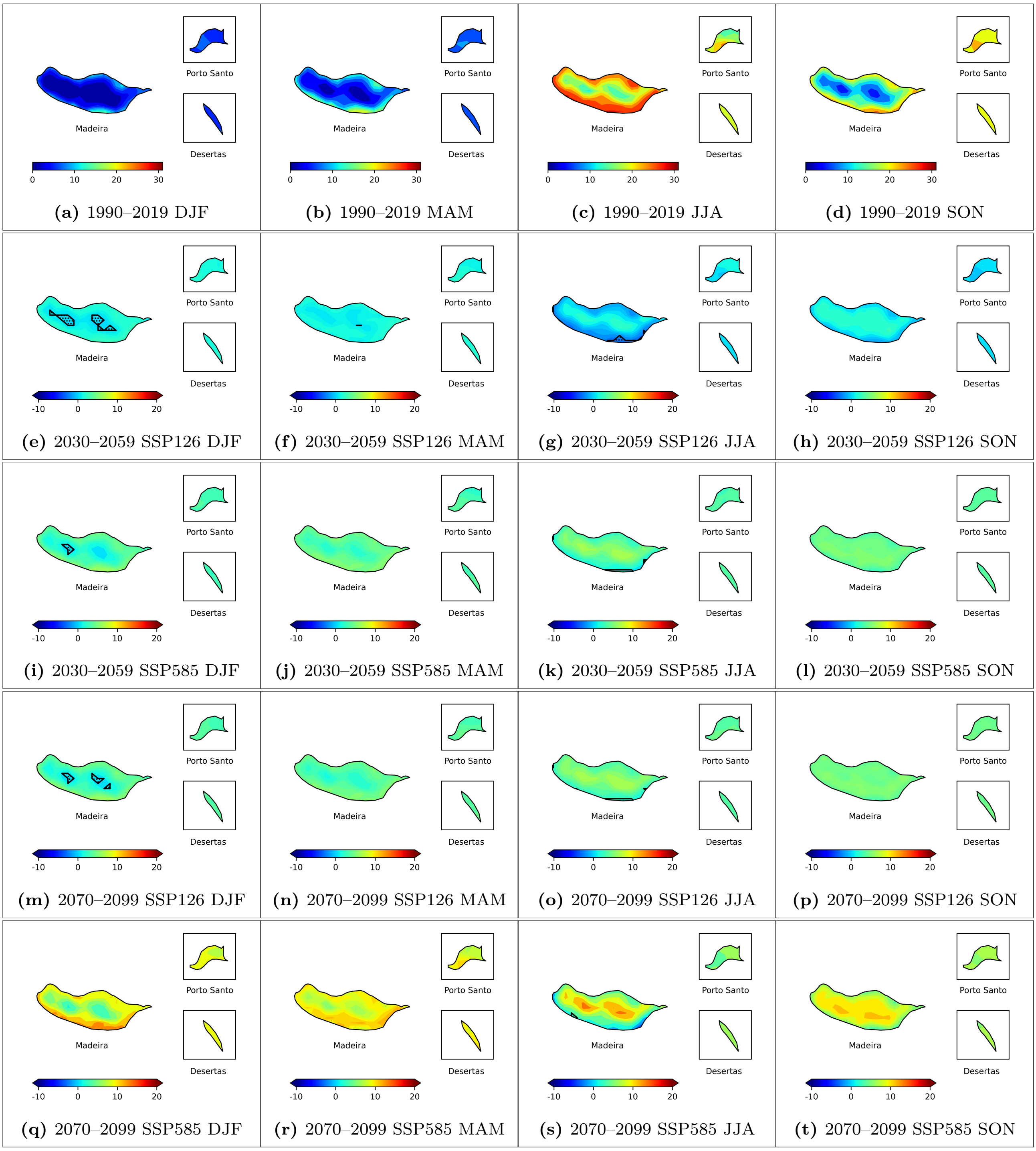

(a) 1990–2019 DJF (b) 1990–2019 MAM (c) 1990–2019 JJA (d) 1990–2019 SON
(e) 2030–2059 SSP126 DJF (f) 2030–2059 SSP126 MAM (g) 2030–2059 SSP126 JJA (h) 2030–2059 SSP126 SON
(i) 2030–2059 SSP585 DJF (j) 2030–2059 SSP585 MAM (k) 2030–2059 SSP585 JJA (l) 2030–2059 SSP585 SON
(m) 2070–2099 SSP126 DJF (n) 2070–2099 SSP126 MAM (o) 2070–2099 SSP126 JJA (p) 2070–2099 SSP126 SON
(q) 2070–2099 SSP585 DJF (r) 2070–2099 SSP585 MAM (s) 2070–2099 SSP585 JJA (t) 2070–2099 SSP585 SON

Figure S16. Simulated observations and projected changes in the **HCIU80** sub-index for **Madeira**. The HCIU80 metric represents the number of excellent days for urban tourism. The top row shows simulated observed values from 1990–2019 for all four seasons (DJF, MAM, JJA, and SON). The subsequent rows indicate projected changes (average seasonal differences in the monthly number of excellent days) for 2030–2059 and 2070–2099 under both the SSP1-2.6 and SSP5-8.5 scenarios, respectively, all relative to the 1990–2019 baseline. Areas with black dots indicate statistically non-significant changes, while non-hatched areas indicate statistically significant changes. These non-significant results reflect variability in the ensemble response rather than the absence of change.

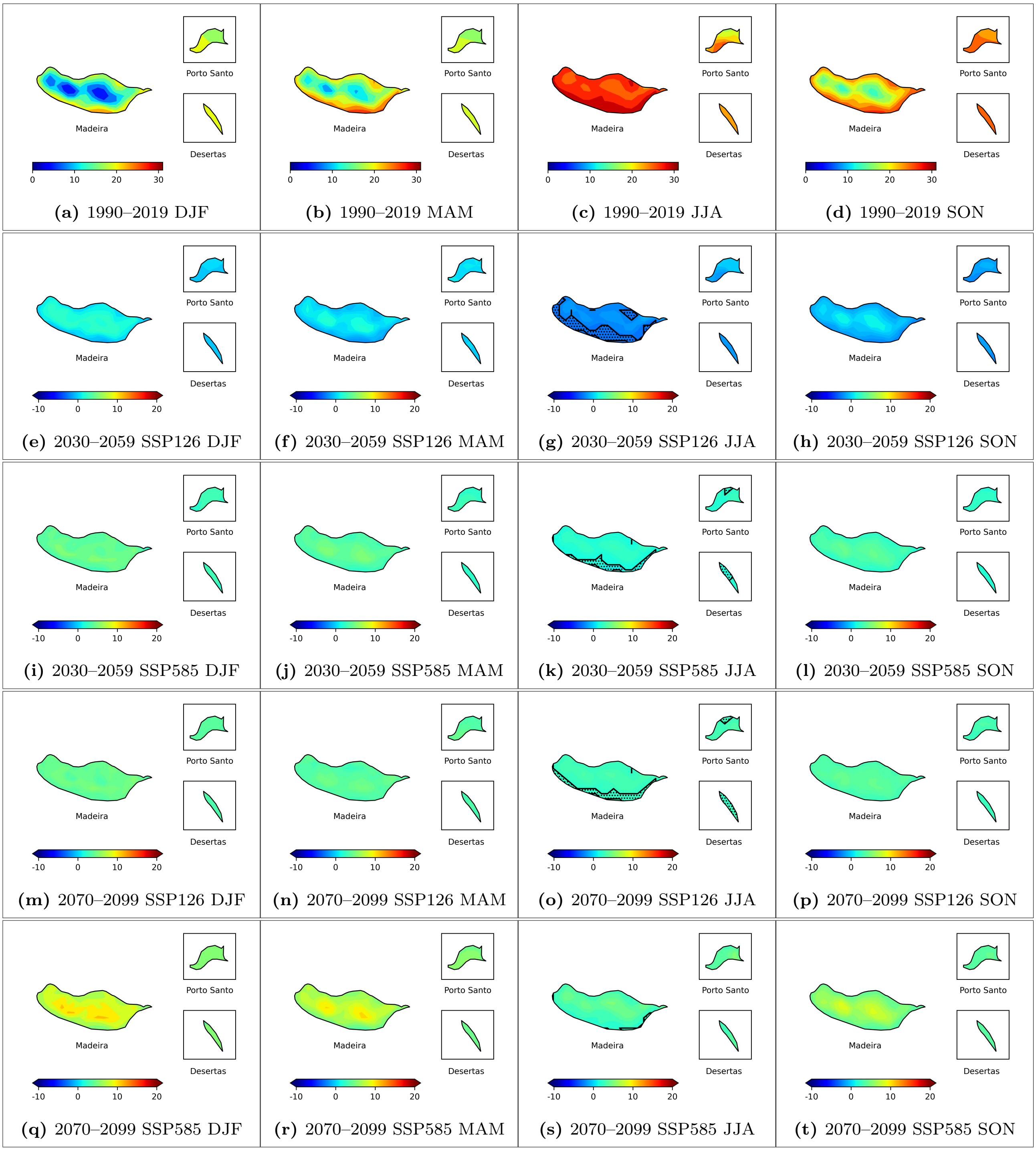

Figure S17. Simulated observations and projected changes in the **HCIB60** sub-index for **Madeira**. The HCIB60 metric represents the number of good days for beach tourism. The top row shows simulated observed values from 1990–2019 for all four seasons (DJF, MAM, JJA, and SON). The subsequent rows indicate projected changes (average seasonal differences in the monthly number of good days) for 2030–2059 and 2070–2099 under both the SSP1-2.6 and SSP5-8.5 scenarios, respectively, all relative to the 1990–2019 baseline. Areas with black dots indicate statistically non-significant changes, while non-hatched areas indicate statistically significant changes. These non-significant results reflect variability in the ensemble response rather than the absence of change.

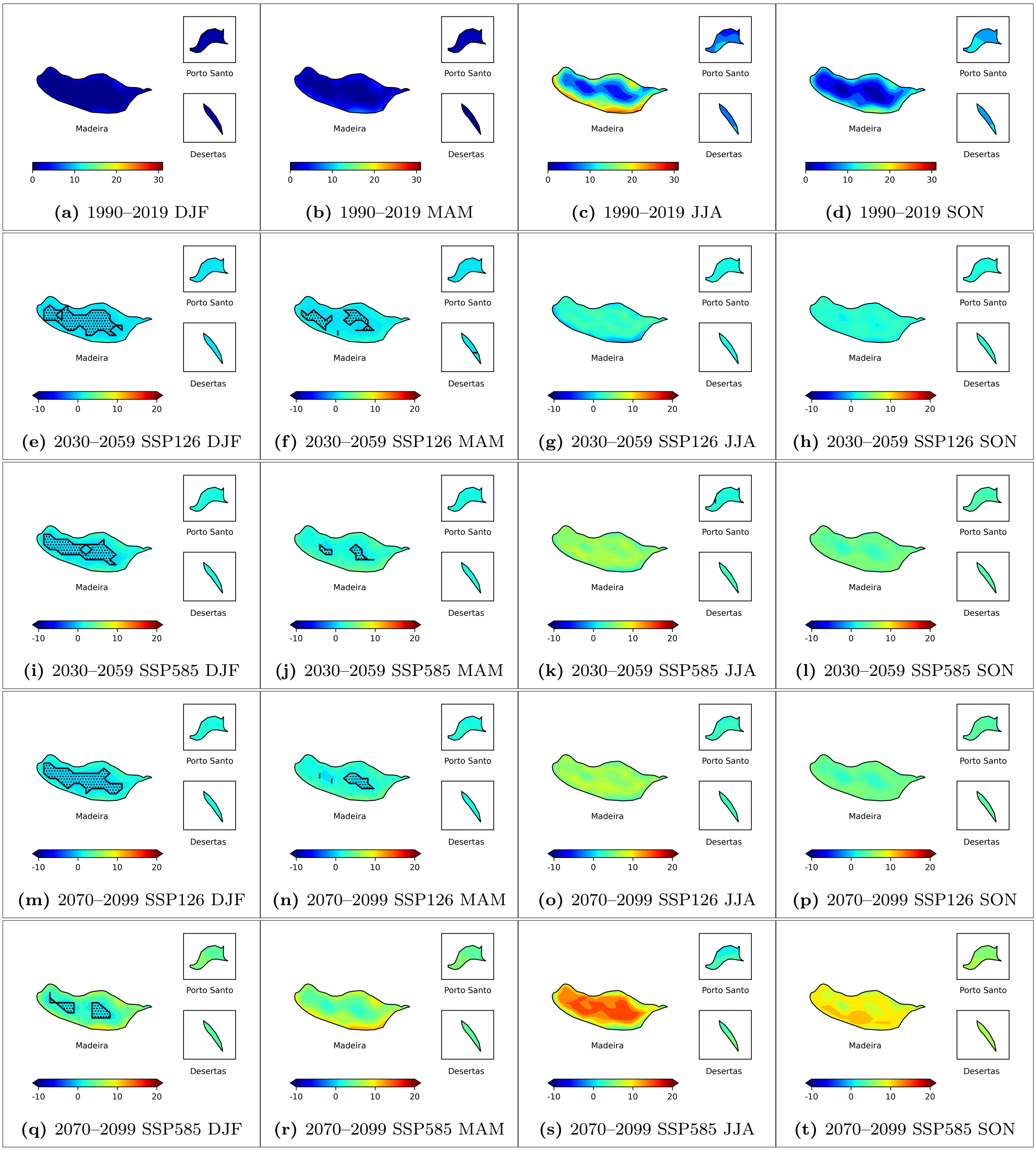

Figure S18. Simulated observations and projected changes in the **HCIB80** sub-index for **Madeira**. The HCIB80 metric represents the number of excellent days for beach tourism. The top row shows simulated observed values from 1990–2019 for all four seasons (DJF, MAM, JJA, and SON). The subsequent rows indicate projected changes (average seasonal differences in the monthly number of excellent days) for 2030–2059 and 2070–2099 under both the SSP1-2.6 and SSP5-8.5 scenarios, respectively, all relative to the 1990–2019 baseline. Areas with black dots indicate statistically non-significant changes, while non-hatched areas indicate statistically significant changes. These non-significant results reflect variability in the ensemble response rather than the absence of change.

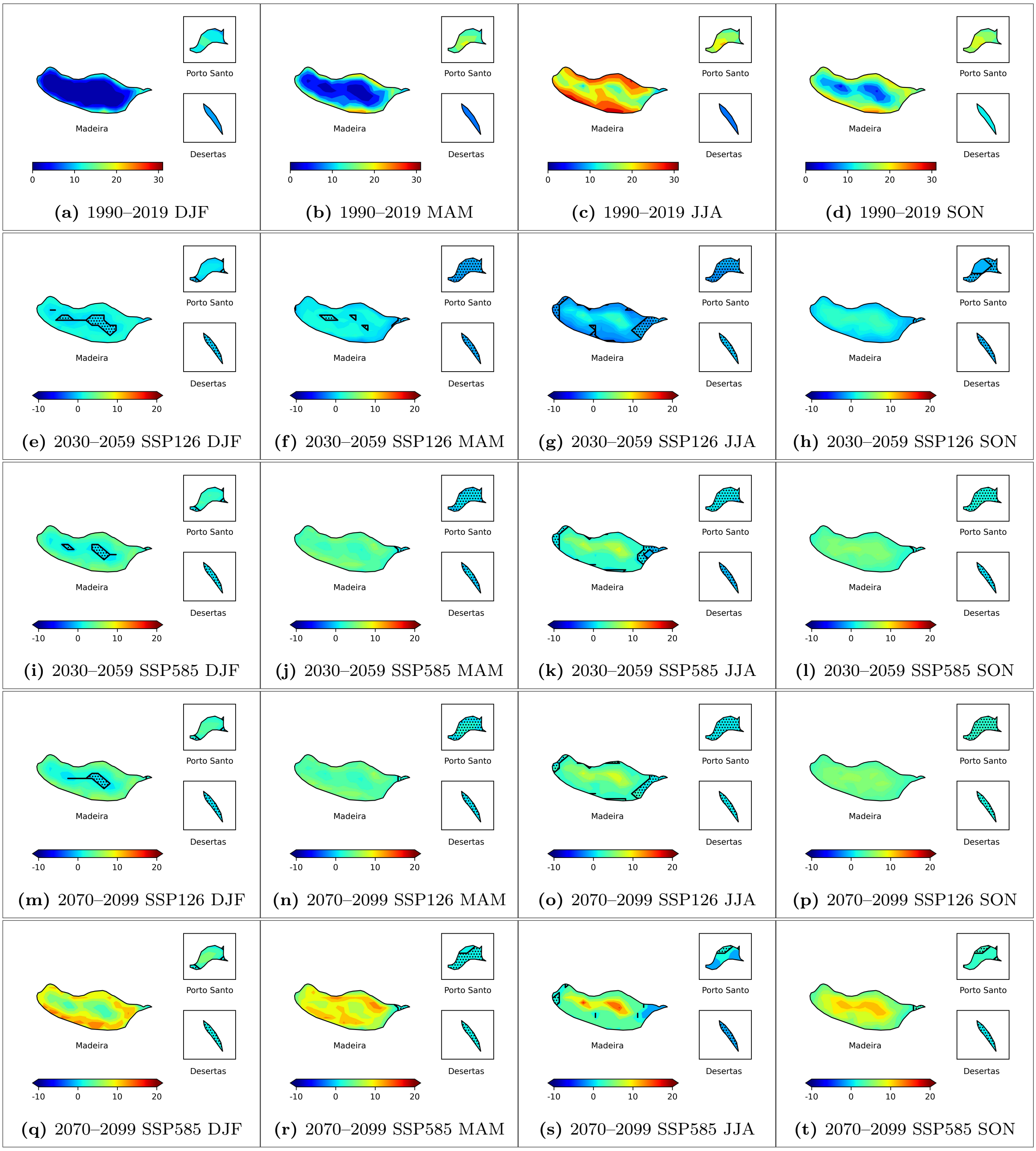

Figure S19. Simulated observations and projected changes in the **CCI05** sub-index for **Madeira**. The CCI05 metric represents the number of good days for nature-based tourism. The top row shows simulated observed values from 1990–2019 for all four seasons (DJF, MAM, JJA, and SON). The subsequent rows indicate projected changes (average seasonal differences in the monthly number of good days) for 2030–2059 and 2070–2099 under both the SSP1-2.6 and SSP5-8.5 scenarios, respectively, all relative to the 1990–2019 baseline. Areas with black dots indicate statistically non-significant changes, while non-hatched areas indicate statistically significant changes. These non-significant results reflect variability in the ensemble response rather than the absence of change.

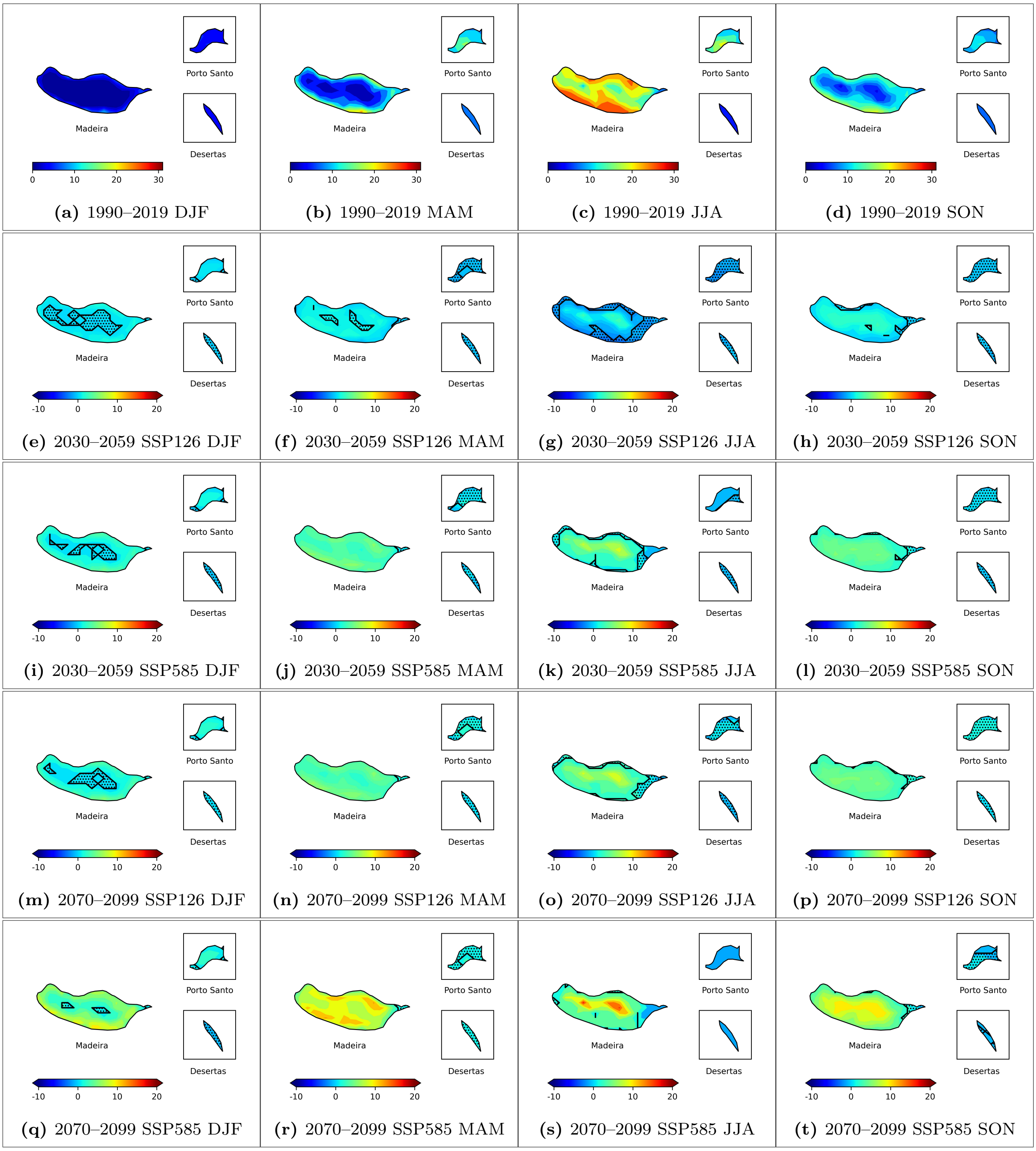

(a) 1990–2019 DJF
(b) 1990–2019 MAM
(c) 1990–2019 JJA
(d) 1990–2019 SON
(e) 2030–2059 SSP126 DJF
(f) 2030–2059 SSP126 MAM
(g) 2030–2059 SSP126 JJA
(h) 2030–2059 SSP126 SON
(i) 2030–2059 SSP585 DJF
(j) 2030–2059 SSP585 MAM
(k) 2030–2059 SSP585 JJA
(l) 2030–2059 SSP585 SON
(m) 2070–2099 SSP126 DJF
(n) 2070–2099 SSP126 MAM
(o) 2070–2099 SSP126 JJA
(p) 2070–2099 SSP126 SON
(q) 2070–2099 SSP585 DJF
(r) 2070–2099 SSP585 MAM
(s) 2070–2099 SSP585 JJA
(t) 2070–2099 SSP585 SON

Figure S20. Simulated observations and projected changes in the **CCI07** sub-index for **Madeira**. The CCI07 metric represents the number of optimal days for nature-based tourism. The top row shows simulated observed values from 1990–2019 for all four seasons (DJF, MAM, JJA, and SON). The subsequent rows indicate projected changes (differences in the average number of optimal days per month) for 2030–2059 and 2070–2099 under both the SSP1-2.6 and SSP5-8.5 scenarios, respectively, all relative to the 1990–2019 baseline. Areas with black dots indicate statistically non-significant changes, while non-hatched areas indicate statistically significant changes. These non-significant results reflect variability in the ensemble response rather than the absence of change.

3.3 The Canary Islands

The Canary Islands, known for their year-round appeal, are projected to see a further increase in tourism-suitable days. The changes are most pronounced under the SSP5-8.5 scenario, which indicates a widespread and statistically significant increase across most tourism indices in both 2030–2059 and 2070–2099 futures. The number of good and excellent urban and beach tourism days is expected to grow. The projections indicate a particularly strong increase in the off-season, with autumn and winter becoming even more favorable for tourism. This could reinforce the islands' status as a popular winter sun destination. Table S4 summarizes these past conditions and future changes.

Table S4. Tourism climate indices for the Canary Islands during the recent past baseline (1990–2019) and projected changes for the near- (2030–2059) and long-term future (2070–2099) under SSP1-2.6 and SSP5-8.5 scenarios.

**Sub-index**	**Baseline conditions (1990–2019)**	**Changes (2030–2059 and 2070–2099)**
TCI60 (Figure S21)	Good tourism conditions range from 20 to 31 days in winter, spring, and autumn, and reach a maximum of 25 to 31 days in summer.	A decrease of 1 to 2 days is projected in 2030–2059 under both scenarios. By 2070–2099, a broader increase of up to 3 days is projected for winter and spring, with little change in summer and autumn.
TCI80 (Figure S22)	Excellent tourism conditions range from 0 to 10 days in winter, increasing to between 5 to 20 days in spring and 15 to 25 days in autumn, and reaching a maximum of 25 to 31 days in summer.	In 2030–2059, both SSP scenarios project an increase of up to 3 days in winter and spring, with small changes in summer and autumn, which is similar to the projected changes for 2070–2099 under the SSP1-2.6 scenario. In contrast, winter and spring of 2070–2099 SSP5-8.5 project a much stronger increase of up to 11 days.
HCIU60 (Figure S23)	Good urban tourism conditions range from 25 to 31 days in all seasons.	There is a widespread decrease of up to 1 day in 2030–2059 for both scenarios. By 2070–2099, conditions return to baseline with little changes across all seasons.
HCIU80 (Figure S24)	Excellent urban tourism conditions range from 0 to 15 days in winter, increasing to between 5 to 20 days in spring, and reaching a maximum of 15 to 25 days in summer and autumn.	There is an increase of 2 to 3 days in winter and spring conditions for both scenarios in 2030–2059 and in 2070–2099 SSP1-2.6, with a smaller increase of up to 1 day in summer and autumn. By 2070–2099, there is a stronger increase in winter and spring of up to 8 days projected for the SSP5-8.5 scenario.
HCIB60 (Figure S25)	Good beach tourism conditions range from 10 to 25 days in winter, increasing to between 20 to 25 days in spring, and reaching a maximum of 25 to 31 days in summer.	There is a widespread small change of up to 1 day projected for 2030–2059 and 2070–2099 under both scenarios, with a slightly broader increase of up to 3 days projected for winter and spring of 2070–2099 SSP5-8.5.
HCIB80 (Figure S26)	Excellent beach tourism conditions range from 0 to 10 days in winter, increasing to between 0 to 15 days in spring and 10 to 20 days in autumn, and reaching a maximum of 10 to 25 days in summer.	For 2030–2059 and 2070–2099 SSP1-2.6, there is an increase of up to 3 days across all seasons. By 2070–2099, the increases become stronger, with SSP5-8.5 projecting a rise of up to 8 days in winter and up to 6 days in spring.
CCI05 (Figure S27)	Good nature-based tourism conditions range from 0 to 15 days in winter and spring, reaching a maximum of 15 to 25 days in summer and autumn.	Both scenarios project a decrease of up to 1 day in 2030–2059, which is similar to the decrease projected for 2070–2099 under the SSP1-2.6 scenario. By 2070–2099 SSP5-8.5, there is a mix of small increases of up to 5 days in the western islands and decreases of up to 2 days in the eastern islands.
CCI07 (Figure S28)	Optimal nature-based tourism conditions range from 0 to 15 days in winter and spring, increasing to between 10 to 20 days in autumn, and reaching a maximum of 15 to 25 days in summer.	Both scenarios project a decrease of up to 1 day in 2030–2059, which is similar to the decrease projected for 2070–2099 under the SSP1-2.6 scenario. By 2070–2099 SSP5-8.5, there is a mix of small increases of up to 5 days in the western islands and decreases of up to 2 days in the eastern islands.

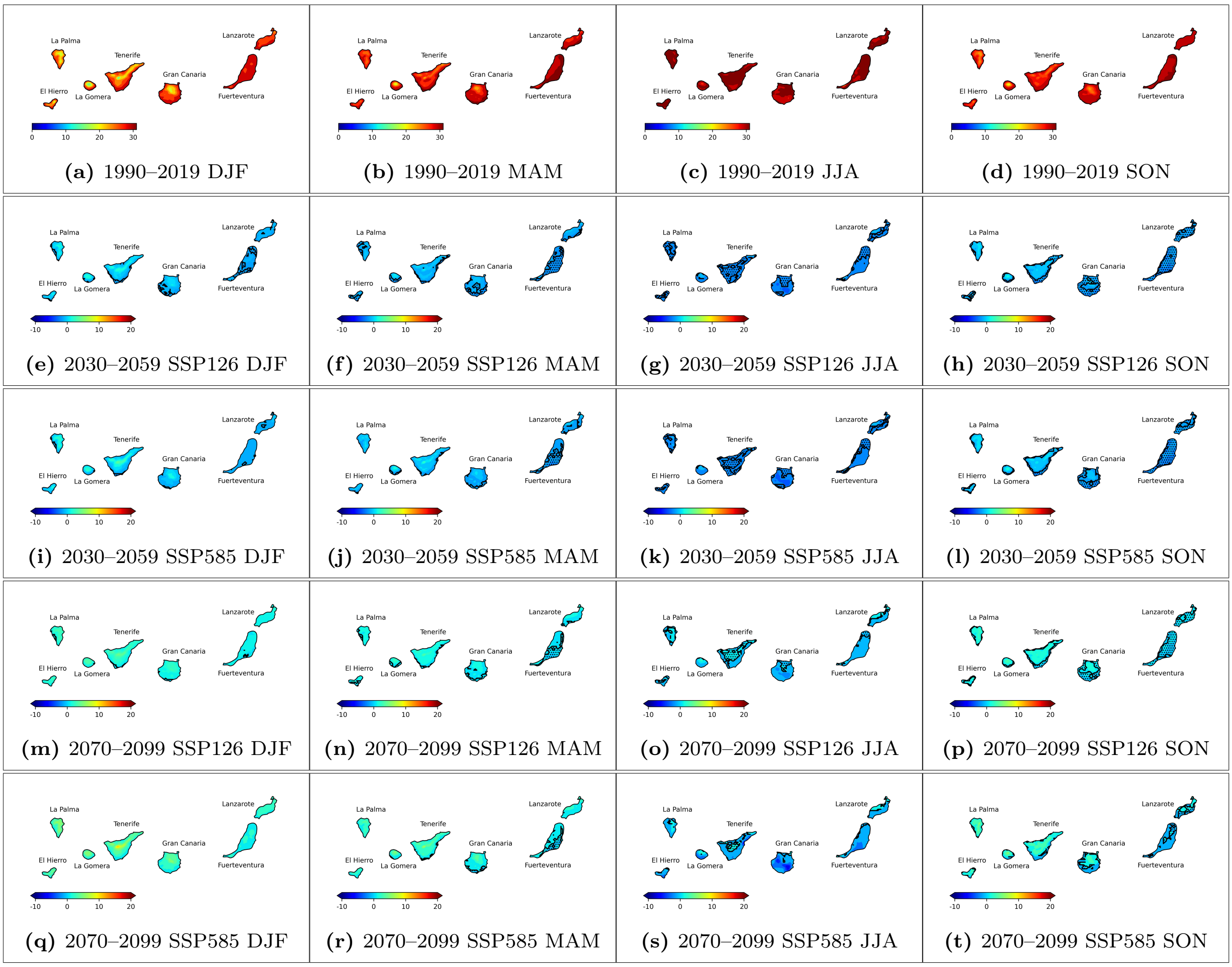

(a) 1990–2019 DJF (b) 1990–2019 MAM (c) 1990–2019 JJA (d) 1990–2019 SON
(e) 2030–2059 SSP126 DJF (f) 2030–2059 SSP126 MAM (g) 2030–2059 SSP126 JJA (h) 2030–2059 SSP126 SON
(i) 2030–2059 SSP585 DJF (j) 2030–2059 SSP585 MAM (k) 2030–2059 SSP585 JJA (l) 2030–2059 SSP585 SON
(m) 2070–2099 SSP126 DJF (n) 2070–2099 SSP126 MAM (o) 2070–2099 SSP126 JJA (p) 2070–2099 SSP126 SON
(q) 2070–2099 SSP585 DJF (r) 2070–2099 SSP585 MAM (s) 2070–2099 SSP585 JJA (t) 2070–2099 SSP585 SON

Figure S21. Simulated observations and projected changes in the **TCI60** sub-index for the **The Canary Islands**. The TCI60 metric represents the number of good days for general-purpose tourism. The top row shows simulated observed values from 1990–2019 for all four seasons (DJF, MAM, JJA, and SON). The subsequent rows indicate projected changes (average seasonal differences in the monthly number of good days) for 2030–2059 and 2070–2099 under both the SSP1-2.6 and SSP5-8.5 scenarios, respectively, all relative to the 1990–2019 baseline. Areas with black dots indicate statistically non-significant changes, while non-hatched areas indicate statistically significant changes. These non-significant results reflect variability in the ensemble response rather than absence of change.

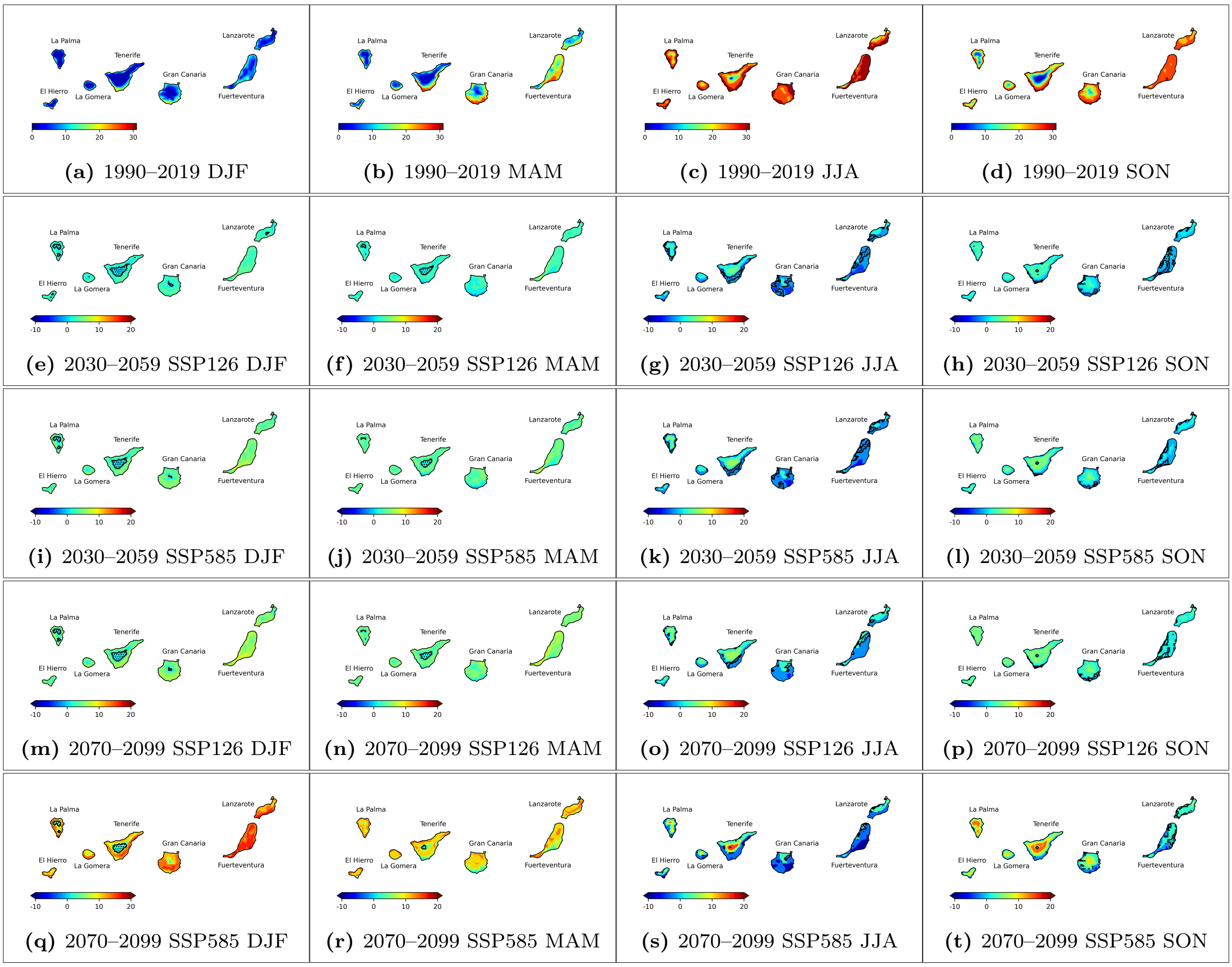

(a) 1990–2019 DJF; (b) 1990–2019 MAM; (c) 1990–2019 JJA; (d) 1990–2019 SON

(e) 2030–2059 SSP126 DJF; (f) 2030–2059 SSP126 MAM; (g) 2030–2059 SSP126 JJA; (h) 2030–2059 SSP126 SON

(i) 2030–2059 SSP585 DJF; (j) 2030–2059 SSP585 MAM; (k) 2030–2059 SSP585 JJA; (l) 2030–2059 SSP585 SON

(m) 2070–2099 SSP126 DJF; (n) 2070–2099 SSP126 MAM; (o) 2070–2099 SSP126 JJA; (p) 2070–2099 SSP126 SON

(q) 2070–2099 SSP585 DJF; (r) 2070–2099 SSP585 MAM; (s) 2070–2099 SSP585 JJA; (t) 2070–2099 SSP585 SON

Figure S22. Simulated observations and projected changes in the **TCI80** sub-index for the **The Canary Islands**. The TCI80 metric represents the number of excellent days for general-purpose tourism. The top row shows simulated observed values from 1990–2019 for all four seasons (DJF, MAM, JJA, and SON). The subsequent rows indicate projected changes (average seasonal differences in the monthly number of excellent days) for 2030–2059 and 2070–2099 under both the SSP1-2.6 and SSP5-8.5 scenarios, respectively, all relative to the 1990–2019 baseline. Areas with black dots indicate statistically non-significant changes, while non-hatched areas indicate statistically significant changes. These non-significant results reflect variability in the ensemble response rather than the absence of change.

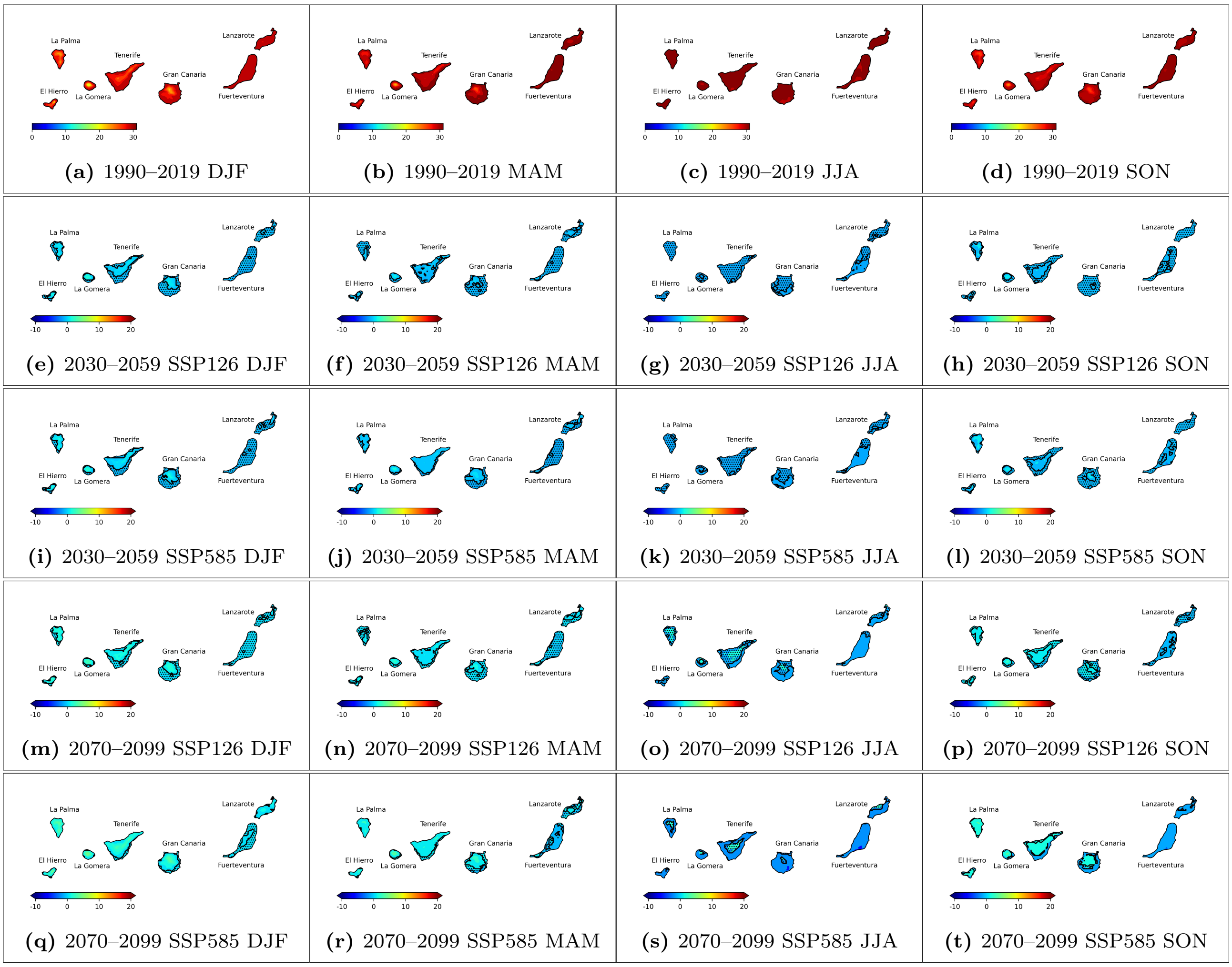

(a) 1990–2019 DJF
(b) 1990–2019 MAM
(c) 1990–2019 JJA
(d) 1990–2019 SON
(e) 2030–2059 SSP126 DJF
(f) 2030–2059 SSP126 MAM
(g) 2030–2059 SSP126 JJA
(h) 2030–2059 SSP126 SON
(i) 2030–2059 SSP585 DJF
(j) 2030–2059 SSP585 MAM
(k) 2030–2059 SSP585 JJA
(l) 2030–2059 SSP585 SON
(m) 2070–2099 SSP126 DJF
(n) 2070–2099 SSP126 MAM
(o) 2070–2099 SSP126 JJA
(p) 2070–2099 SSP126 SON
(q) 2070–2099 SSP585 DJF
(r) 2070–2099 SSP585 MAM
(s) 2070–2099 SSP585 JJA
(t) 2070–2099 SSP585 SON

Figure S23. Simulated observations and projected changes in the **HCIU60** sub-index for the **The Canary Islands**. The HCIU60 metric represents the number of good days for urban tourism. The top row shows simulated observed values from 1990–2019 for all four seasons (DJF, MAM, JJA, and SON). The subsequent rows indicate projected changes (average seasonal differences in the monthly number of good days) for 2030–2059 and 2070–2099 under both the SSP1-2.6 and SSP5-8.5 scenarios, respectively, all relative to the 1990–2019 baseline. Areas with black dots indicate statistically non-significant changes, while non-hatched areas indicate statistically significant changes. These non-significant results reflect variability in the ensemble response rather than the absence of change.

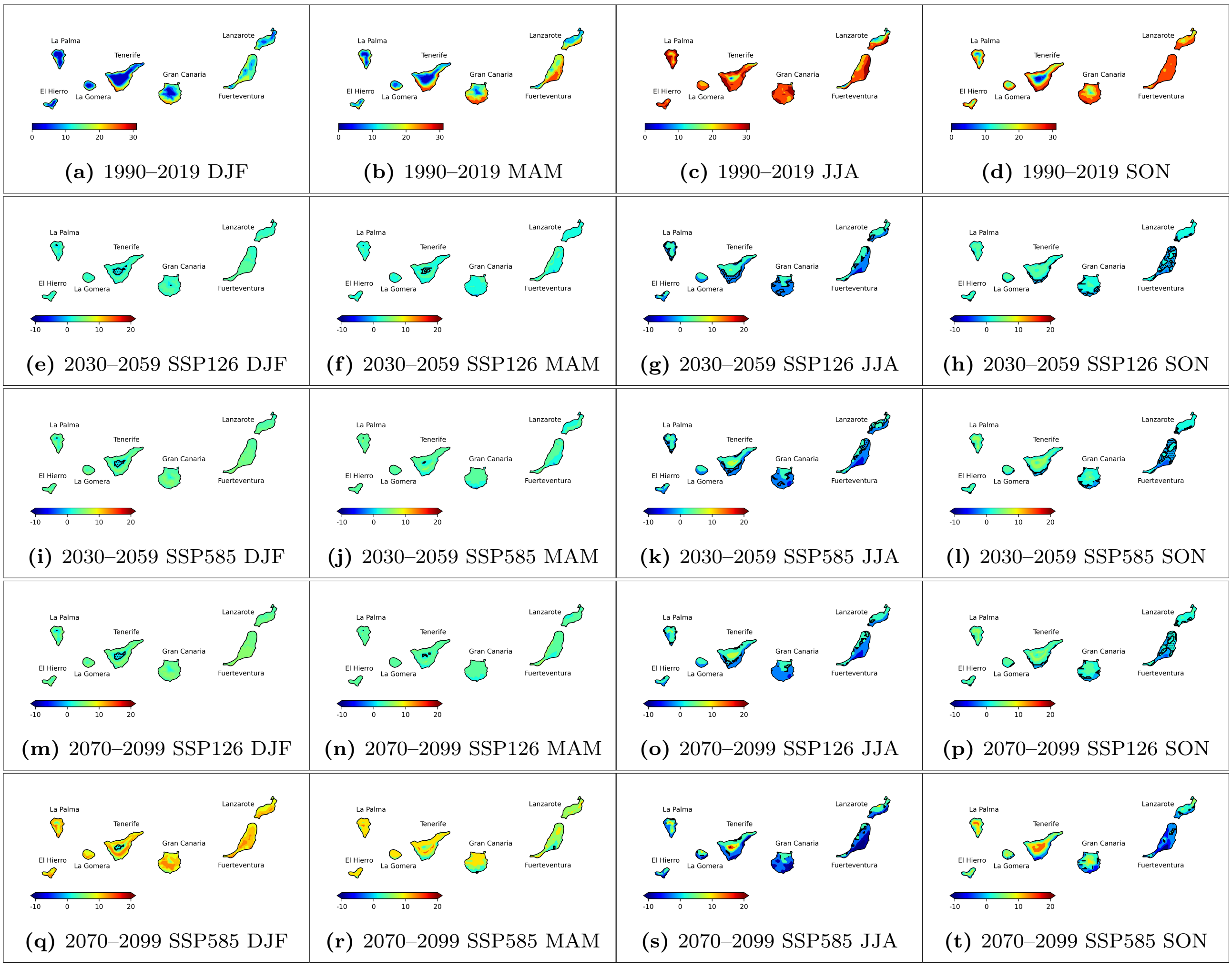

(a) 1990–2019 DJF
(b) 1990–2019 MAM
(c) 1990–2019 JJA
(d) 1990–2019 SON
(e) 2030–2059 SSP126 DJF
(f) 2030–2059 SSP126 MAM
(g) 2030–2059 SSP126 JJA
(h) 2030–2059 SSP126 SON
(i) 2030–2059 SSP585 DJF
(j) 2030–2059 SSP585 MAM
(k) 2030–2059 SSP585 JJA
(l) 2030–2059 SSP585 SON
(m) 2070–2099 SSP126 DJF
(n) 2070–2099 SSP126 MAM
(o) 2070–2099 SSP126 JJA
(p) 2070–2099 SSP126 SON
(q) 2070–2099 SSP585 DJF
(r) 2070–2099 SSP585 MAM
(s) 2070–2099 SSP585 JJA
(t) 2070–2099 SSP585 SON

Figure S24. Simulated observations and projected changes in the **HCIU80** sub-index for the **The Canary Islands**. The HCIU80 metric represents the number of excellent days for urban tourism. The top row shows simulated observed values from 1990–2019 for all four seasons (DJF, MAM, JJA, and SON). The subsequent rows indicate projected changes (average seasonal differences in the monthly number of excellent days) for 2030–2059 and 2070–2099 under both the SSP1-2.6 and SSP5-8.5 scenarios, respectively, all relative to the 1990–2019 baseline. Areas with black dots indicate statistically non-significant changes, while non-hatched areas indicate statistically significant changes. These non-significant results reflect variability in the ensemble response rather than the absence of change.

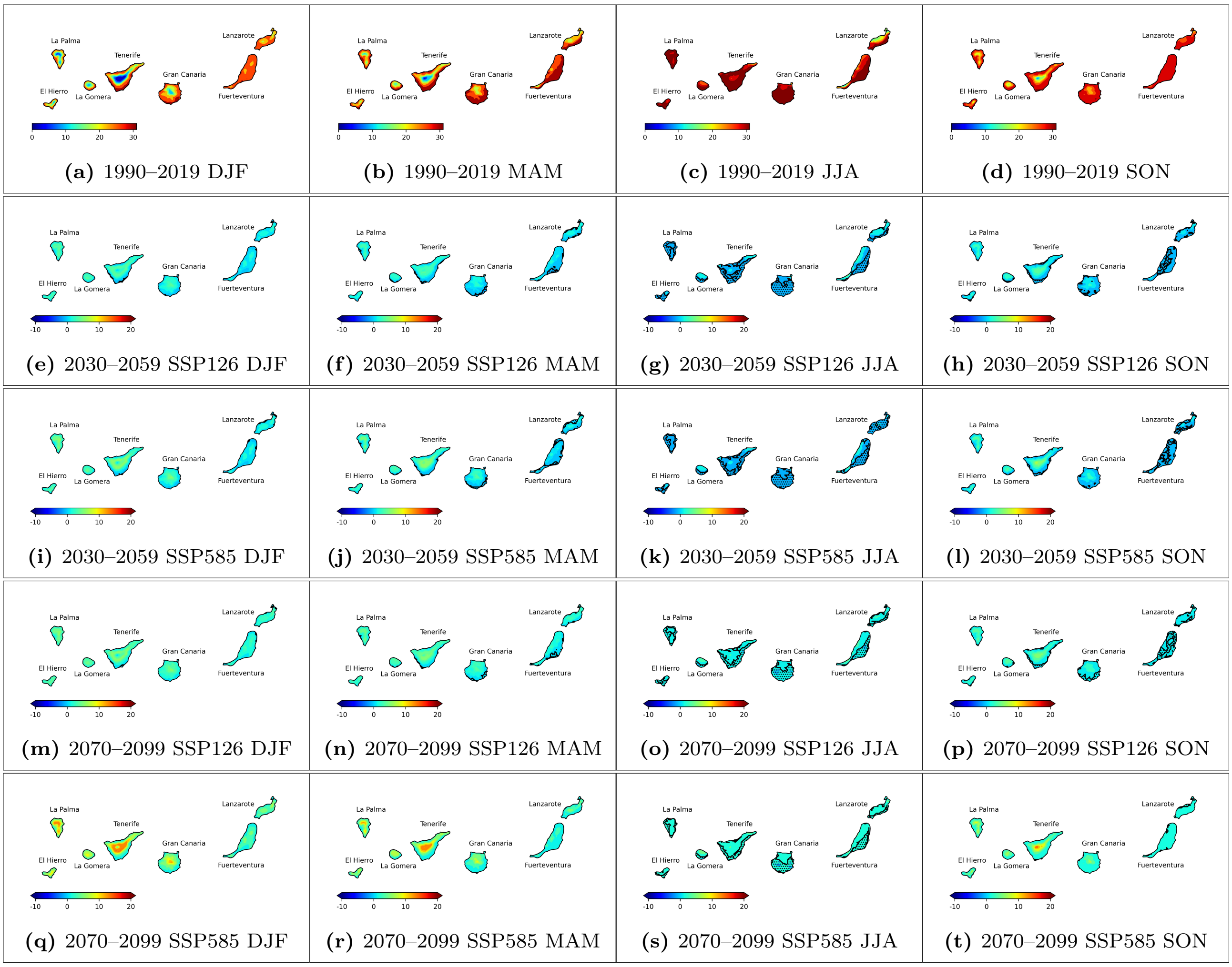

Figure S25. Simulated observations and projected changes in the **HCIB60** sub-index for the **The Canary Islands**. The HCIB60 metric represents the number of good days for beach tourism. The top row shows simulated observed values from 1990–2019 for all four seasons (DJF, MAM, JJA, and SON). The subsequent rows indicate projected changes (average seasonal differences in the monthly number of good days) for 2030–2059 and 2070–2099 under both the SSP1-2.6 and SSP5-8.5 scenarios, respectively, all relative to the 1990–2019 baseline. Areas with black dots indicate statistically non-significant changes, while non-hatched areas indicate statistically significant changes. These non-significant results reflect variability in the ensemble response rather than the absence of change.

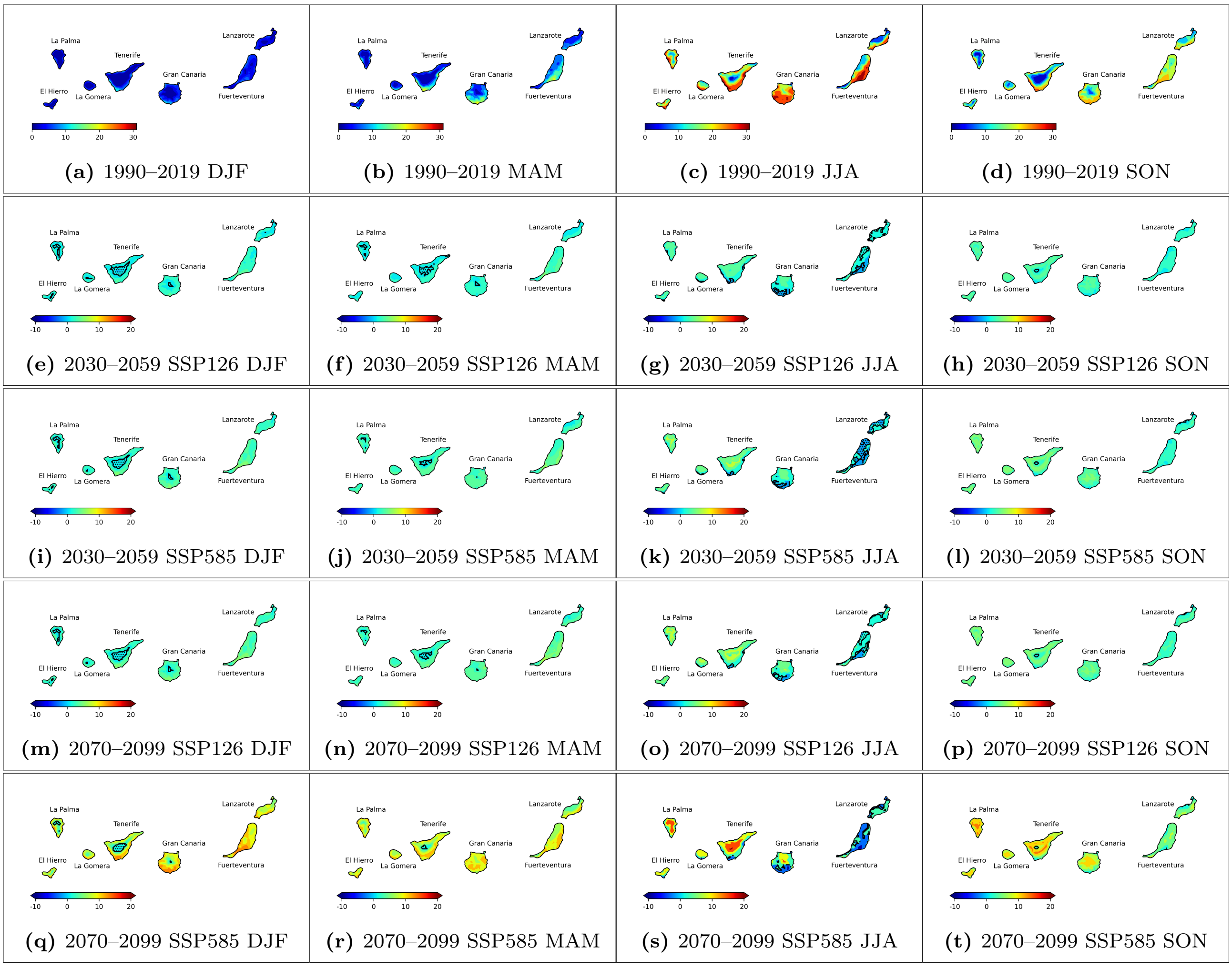

(a) 1990–2019 DJF | **(b)** 1990–2019 MAM | **(c)** 1990–2019 JJA | **(d)** 1990–2019 SON

(e) 2030–2059 SSP126 DJF | **(f)** 2030–2059 SSP126 MAM | **(g)** 2030–2059 SSP126 JJA | **(h)** 2030–2059 SSP126 SON

(i) 2030–2059 SSP585 DJF | **(j)** 2030–2059 SSP585 MAM | **(k)** 2030–2059 SSP585 JJA | **(l)** 2030–2059 SSP585 SON

(m) 2070–2099 SSP126 DJF | **(n)** 2070–2099 SSP126 MAM | **(o)** 2070–2099 SSP126 JJA | **(p)** 2070–2099 SSP126 SON

(q) 2070–2099 SSP585 DJF | **(r)** 2070–2099 SSP585 MAM | **(s)** 2070–2099 SSP585 JJA | **(t)** 2070–2099 SSP585 SON

Figure S26. Simulated observations and projected changes in the **HCIB80** sub-index for the **The Canary Islands**. The HCIB80 metric represents the number of excellent days for beach tourism. The top row shows simulated observed values from 1990–2019 for all four seasons (DJF, MAM, JJA, and SON). The subsequent rows indicate projected changes (average seasonal differences in the monthly number of excellent days) for 2030–2059 and 2070–2099 under both the SSP1-2.6 and SSP5-8.5 scenarios, respectively, all relative to the 1990–2019 baseline. Areas with black dots indicate statistically non-significant changes, while non-hatched areas indicate statistically significant changes. These non-significant results reflect variability in the ensemble response rather than the absence of change.

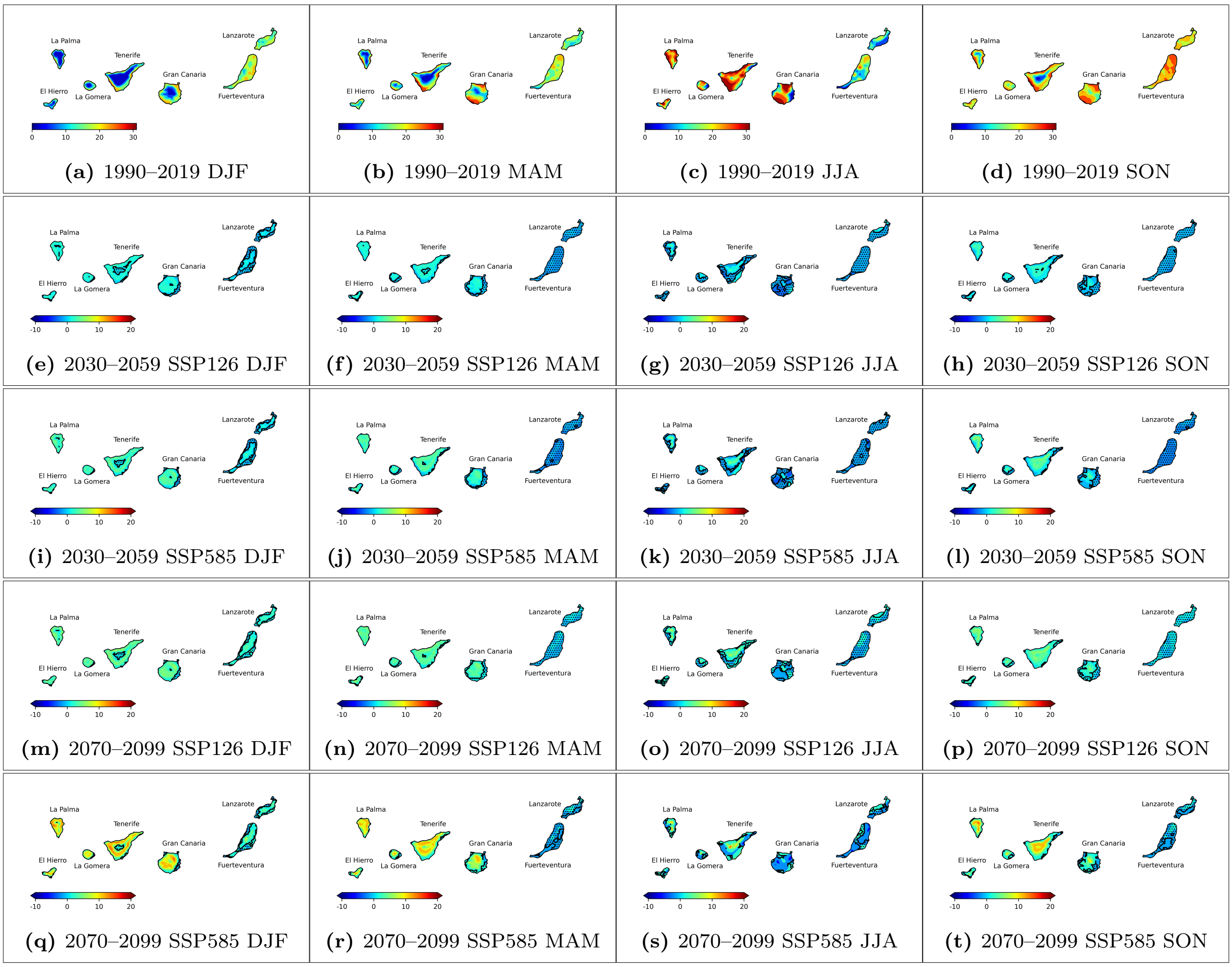

(a) 1990–2019 DJF **(b)** 1990–2019 MAM **(c)** 1990–2019 JJA **(d)** 1990–2019 SON

(e) 2030–2059 SSP126 DJF **(f)** 2030–2059 SSP126 MAM **(g)** 2030–2059 SSP126 JJA **(h)** 2030–2059 SSP126 SON

(i) 2030–2059 SSP585 DJF **(j)** 2030–2059 SSP585 MAM **(k)** 2030–2059 SSP585 JJA **(l)** 2030–2059 SSP585 SON

(m) 2070–2099 SSP126 DJF **(n)** 2070–2099 SSP126 MAM **(o)** 2070–2099 SSP126 JJA **(p)** 2070–2099 SSP126 SON

(q) 2070–2099 SSP585 DJF **(r)** 2070–2099 SSP585 MAM **(s)** 2070–2099 SSP585 JJA **(t)** 2070–2099 SSP585 SON

Figure S27. Simulated observations and projected changes in the **CCI05** sub-index for the **The Canary Islands**. The CCI05 metric represents the number of good days for nature-based tourism. The top row shows simulated observed values from 1990–2019 for all four seasons (DJF, MAM, JJA, and SON). The subsequent rows indicate projected changes (average seasonal differences in the monthly number of good days) for 2030–2059 and 2070–2099 under both the SSP1-2.6 and SSP5-8.5 scenarios, respectively, all relative to the 1990–2019 baseline. Areas with black dots indicate statistically non-significant changes, while non-hatched areas indicate statistically significant changes. These non-significant results reflect variability in the ensemble response rather than the absence of change.

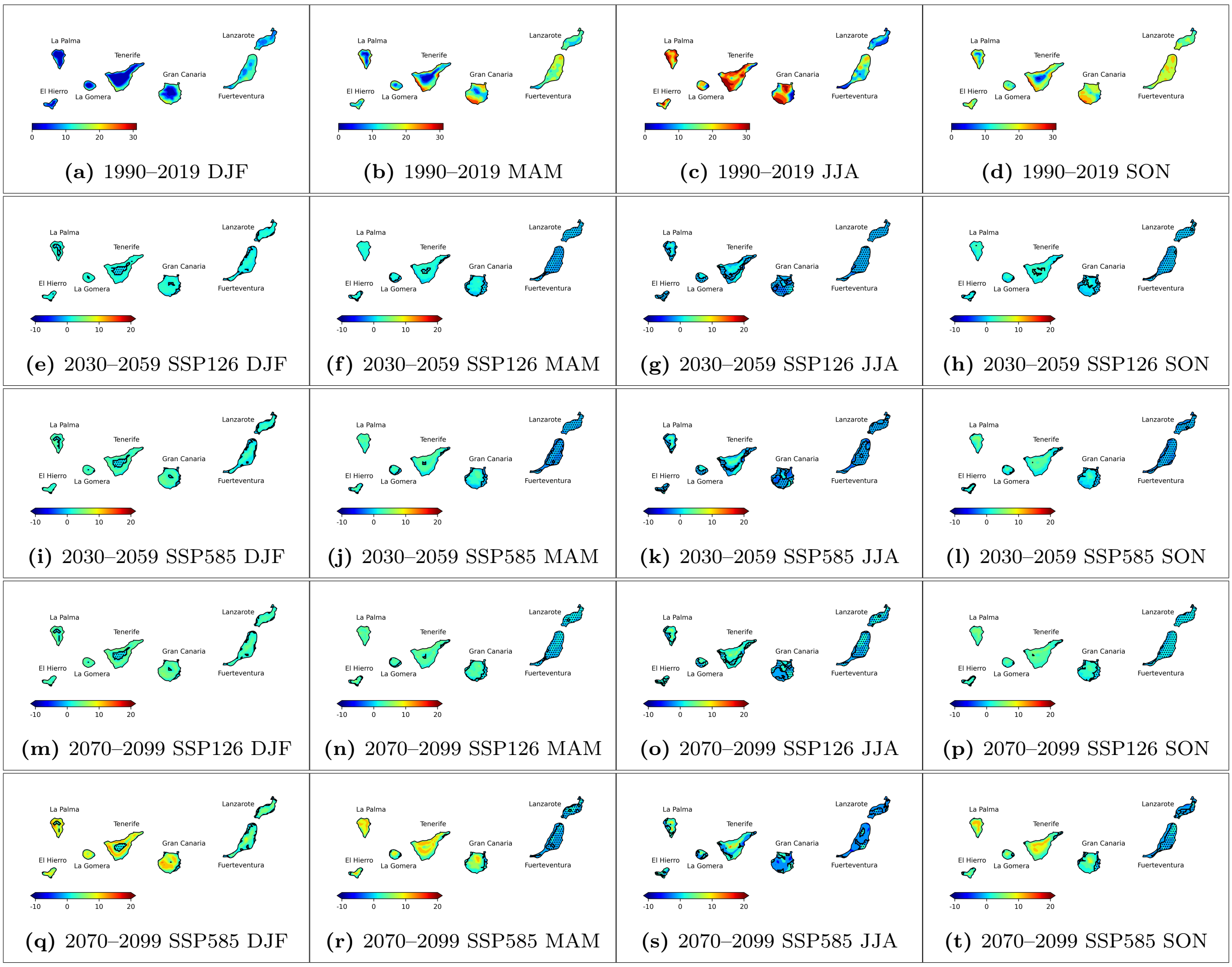

Figure S28. Simulated observations and projected changes in the **CCI07** sub-index for the **The Canary Islands**. The CCI07 metric represents the number of optimal days for nature-based tourism. The top row shows simulated observed values from 1990–2019 for all four seasons (DJF, MAM, JJA, and SON). The subsequent rows indicate projected changes (differences in the average number of optimal days per month) for 2030–2059 and 2070–2099 under both the SSP1-2.6 and SSP5-8.5 scenarios, respectively, all relative to the 1990–2019 baseline. Areas with black dots indicate statistically non-significant changes, while non-hatched areas indicate statistically significant changes. These non-significant results reflect variability in the ensemble response rather than the absence of change.

3.4 Cabo Verde

Cabo Verde currently has good tourism conditions across all seasons. However, the SSP5-8.5 scenario projects an increase in the number of suitable days across both 2030–2059 and 2070–2099 future periods. This is most notable in the historically less optimal seasons of autumn and winter, which are projected to see a large increase in the number of good and excellent tourism days. This trend applies to general, urban, beach, and nature-based tourism. For nature-based tourism, which currently has fewer good days in winter and spring, the SSP5-8.5 scenario projects a widespread and statistically significant increase of 0 to 10 days per month, with some areas seeing an increase of up to 20 days per month. Table S5 summarizes these past conditions and future changes.

Table S5. Tourism climate indices for Cabo Verde during the recent past baseline (1990–2019) and projected changes for the near- (2030–2059) and long-term future (2070–2099) under SSP1-2.6 and SSP5-8.5 scenarios.

**Sub-index**	**Baseline conditions (1990–2019)**	**Changes (2030–2059 and 2070–2099)**
TCI60 (Figure S29)	Good tourism conditions range from 20 to 31 days in all seasons.	The SSP5-8.5 scenario projects a widespread increase of up to 4 days in 2030–2059, especially in winter and spring, but a notable decrease in autumn of up to 11 days in 2070–2099. The SSP1-2.6 scenario projects a decrease of up to 3 days in 2030–2059 but a strong increase of up to 4 days across most seasons in 2070–2099.
TCI80 (Figure S30)	Excellent tourism conditions range from 15 to 25 days in summer and autumn, and up to 15 to 31 days in winter and spring.	Both scenarios project a decrease in autumn for both future periods. Winter and spring improve by up to 4 days under SSP5-8.5 in 2030–2059. In 2070–2099, SSP1-2.6 shows an increase of up to 4 days in winter and spring, whereas SSP5-8.5 projects a mixed trend with a relevant decrease of up to 16 days in autumn.
HCIU60 (Figure S31)	Good urban tourism conditions range from 20 to 31 days in all seasons.	Both scenarios project a widespread increase of up to 4 days in 2030–2059. In 2070–2099, the SSP1-2.6 scenario continues this increase, but the SSP5-8.5 scenario projects a strong decrease in autumn of up to 7 days.
HCIU80 (Figure S32)	Excellent urban tourism conditions range from 10 to 20 days in autumn, increasing to 15 to 25 days in winter, and up to 20 to 25 days in spring and summer.	Both scenarios project a general increase in winter and spring in 2030–2059 of up to 5 days. However, a relevant decrease is projected for autumn and summer, which is much more pronounced in 2070–2099, especially under SSP5-8.5 with a decrease of up to 12 days in autumn.
HCIB60 (Figure S33)	Good beach tourism conditions range from 20 to 25 days in winter and autumn, reaching a maximum of 25 to 31 days in spring and summer.	Both scenarios in 2030–2059 project a widespread increase of up to 5 days across all seasons. In 2070–2099, the SSP1-2.6 scenario is projected to continue this positive trend, while the SSP5-8.5 scenario projects a decrease of up to 2 day in autumn.
HCIB80 (Figure S34)	Excellent beach tourism conditions range from 5 to 15 days in winter, increasing to 10 to 20 days in autumn, and reaching a maximum of 15 to 25 days in spring and summer.	Both scenarios project an increase of up to 6 days in winter, spring, and summer. In 2070–2099, the SSP5-8.5 scenario shows a stronger increase, reaching up to 10 days in winter and spring, though autumn shows decreases of up to 5 days.
CCI05 (Figure S35)	Good nature-based tourism conditions range from 0 to 20 days in winter and spring, and reaching a maximum of 15 to 25 days in summer and autumn.	SSP1-2.6 shows an small increase of up to 1 day in 2030–2059, while SSP5-8.5 projects a widespread increase of up to 4 days across all seasons. In 2070–2099, both scenarios indicate similar widespread increases across all seasons.
CCI07 (Figure S36)	Optimal nature-based tourism conditions range from 0 to 20 days in winter and spring, and reaching a maximum of 15 to 25 days in summer and autumn.	The SSP5-8.5 scenario projects a widespread increase of up to 6 days for both periods. The SSP1-2.6 scenario shows a mixed trend in 2030–2059, but a strong increase of up to 4 days across all seasons in 2070–2099.

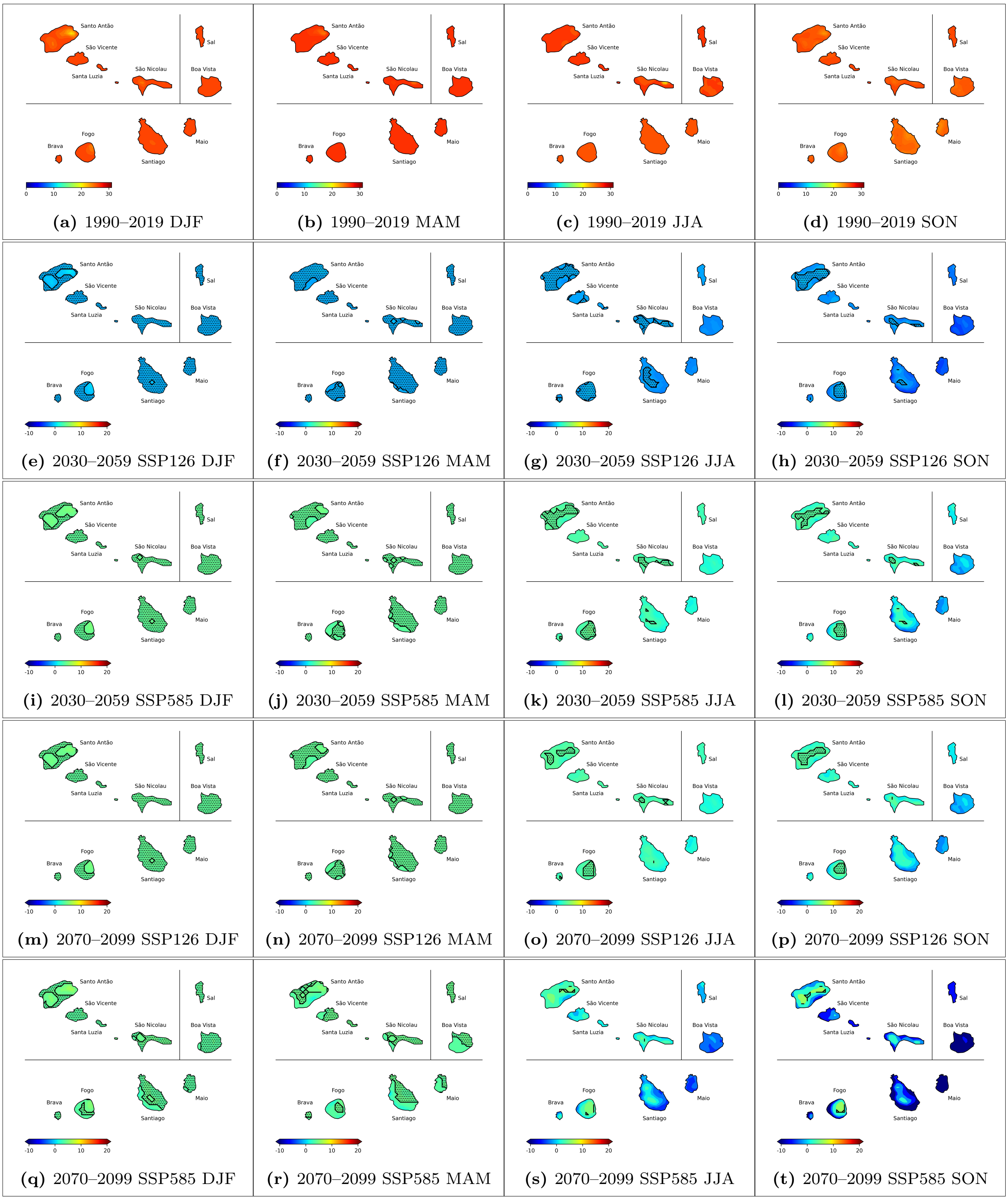

(a) 1990–2019 DJF (b) 1990–2019 MAM (c) 1990–2019 JJA (d) 1990–2019 SON
(e) 2030–2059 SSP126 DJF (f) 2030–2059 SSP126 MAM (g) 2030–2059 SSP126 JJA (h) 2030–2059 SSP126 SON
(i) 2030–2059 SSP585 DJF (j) 2030–2059 SSP585 MAM (k) 2030–2059 SSP585 JJA (l) 2030–2059 SSP585 SON
(m) 2070–2099 SSP126 DJF (n) 2070–2099 SSP126 MAM (o) 2070–2099 SSP126 JJA (p) 2070–2099 SSP126 SON
(q) 2070–2099 SSP585 DJF (r) 2070–2099 SSP585 MAM (s) 2070–2099 SSP585 JJA (t) 2070–2099 SSP585 SON

Figure S29. Simulated observations and projected changes in the **TCI60** sub-index for **Cabo Verde**. The TCI60 metric represents the number of good days for general-purpose tourism. The top row shows simulated observed values from 1990–2019 for all four seasons (DJF, MAM, JJA, and SON). The subsequent rows indicate projected changes (average seasonal differences in the monthly number of good days) for 2030–2059 and 2070–2099 under both the SSP1-2.6 and SSP5-8.5 scenarios, respectively, all relative to the 1990–2019 baseline. Areas with black dots indicate statistically non-significant changes, while non-hatched areas indicate statistically significant changes. These non-significant results reflect variability in the ensemble response rather than the absence of change.

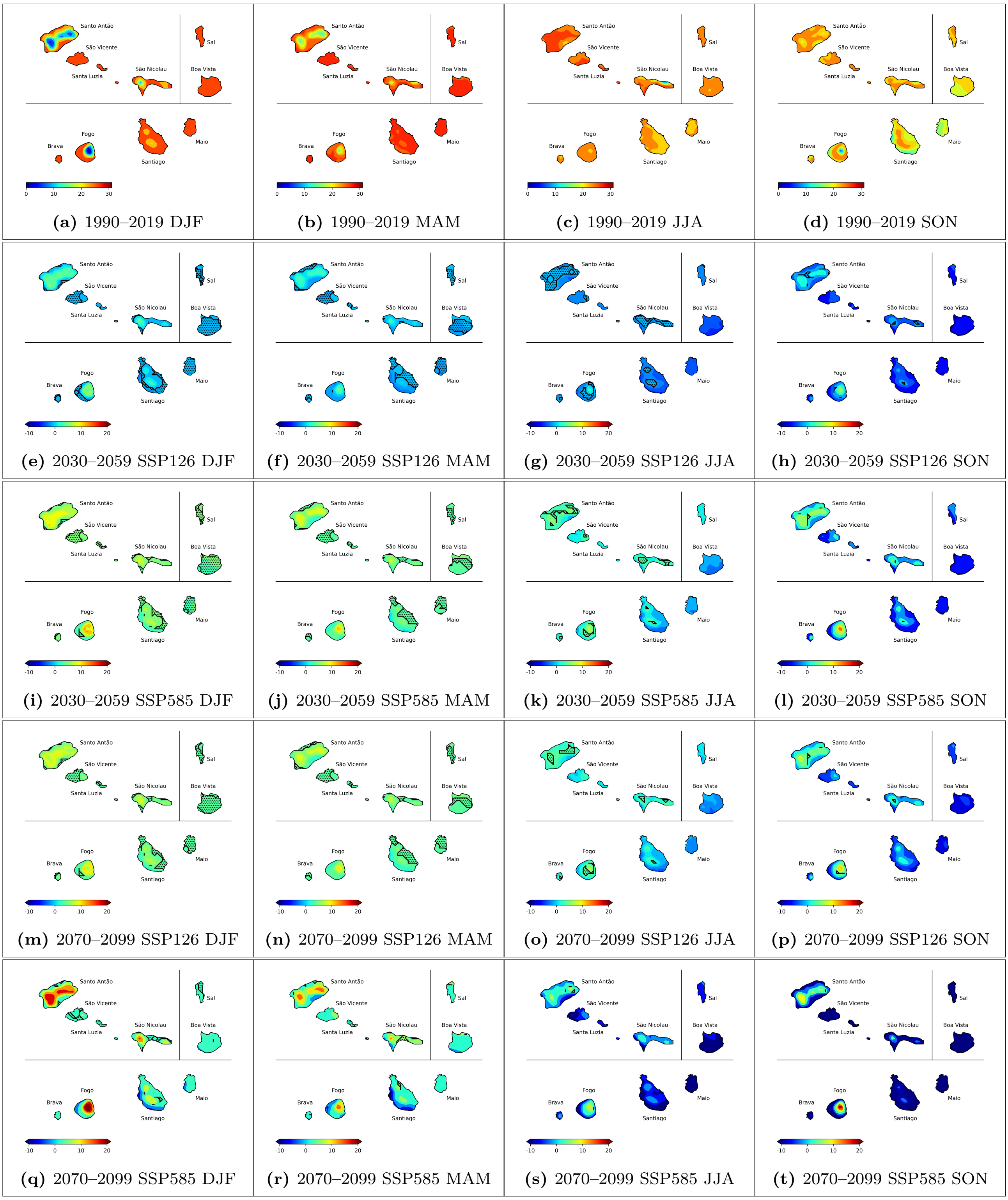

(a) 1990–2019 DJF
(b) 1990–2019 MAM
(c) 1990–2019 JJA
(d) 1990–2019 SON
(e) 2030–2059 SSP126 DJF
(f) 2030–2059 SSP126 MAM
(g) 2030–2059 SSP126 JJA
(h) 2030–2059 SSP126 SON
(i) 2030–2059 SSP585 DJF
(j) 2030–2059 SSP585 MAM
(k) 2030–2059 SSP585 JJA
(l) 2030–2059 SSP585 SON
(m) 2070–2099 SSP126 DJF
(n) 2070–2099 SSP126 MAM
(o) 2070–2099 SSP126 JJA
(p) 2070–2099 SSP126 SON
(q) 2070–2099 SSP585 DJF
(r) 2070–2099 SSP585 MAM
(s) 2070–2099 SSP585 JJA
(t) 2070–2099 SSP585 SON

Figure S30. Simulated observations and projected changes in the **TCI80** sub-index for **Cabo Verde**. The TCI80 metric represents the number of excellent days for general-purpose tourism. The top row shows simulated observed values from 1990–2019 for all four seasons (DJF, MAM, JJA, and SON). The subsequent rows indicate projected changes (average seasonal differences in the monthly number of excellent days) for 2030–2059 and 2070–2099 under both the SSP1-2.6 and SSP5-8.5 scenarios, respectively, all relative to the 1990–2019 baseline. Areas with black dots indicate statistically non-significant changes, while non-hatched areas indicate statistically significant changes. These non-significant results reflect variability in the ensemble response rather than the absence of change.

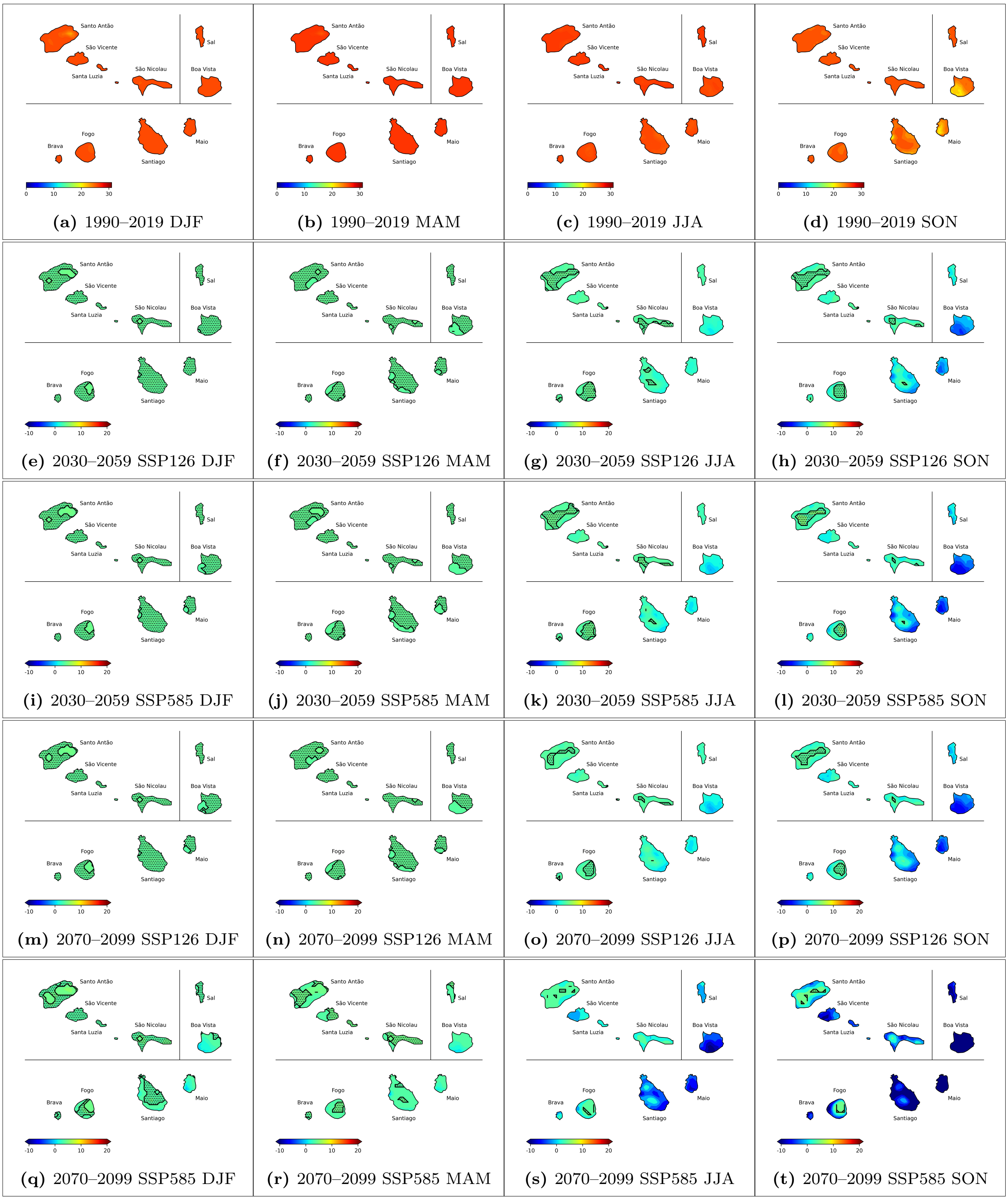

(a) 1990–2019 DJF; (b) 1990–2019 MAM; (c) 1990–2019 JJA; (d) 1990–2019 SON;
(e) 2030–2059 SSP126 DJF; (f) 2030–2059 SSP126 MAM; (g) 2030–2059 SSP126 JJA; (h) 2030–2059 SSP126 SON;
(i) 2030–2059 SSP585 DJF; (j) 2030–2059 SSP585 MAM; (k) 2030–2059 SSP585 JJA; (l) 2030–2059 SSP585 SON;
(m) 2070–2099 SSP126 DJF; (n) 2070–2099 SSP126 MAM; (o) 2070–2099 SSP126 JJA; (p) 2070–2099 SSP126 SON;
(q) 2070–2099 SSP585 DJF; (r) 2070–2099 SSP585 MAM; (s) 2070–2099 SSP585 JJA; (t) 2070–2099 SSP585 SON

Figure S31. Simulated observations and projected changes in the **HCIU60** sub-index for **Cabo Verde**. The HCIU60 metric represents the number of good days for urban tourism. The top row shows simulated observed values from 1990–2019 for all four seasons (DJF, MAM, JJA, and SON). The subsequent rows indicate projected changes (average seasonal differences in the monthly number of good days) for 2030–2059 and 2070–2099 under both the SSP1-2.6 and SSP5-8.5 scenarios, respectively, all relative to the 1990–2019 baseline. Areas with black dots indicate statistically non-significant changes, while non-hatched areas indicate statistically significant changes. These non-significant results reflect variability in the ensemble response rather than the absence of change.

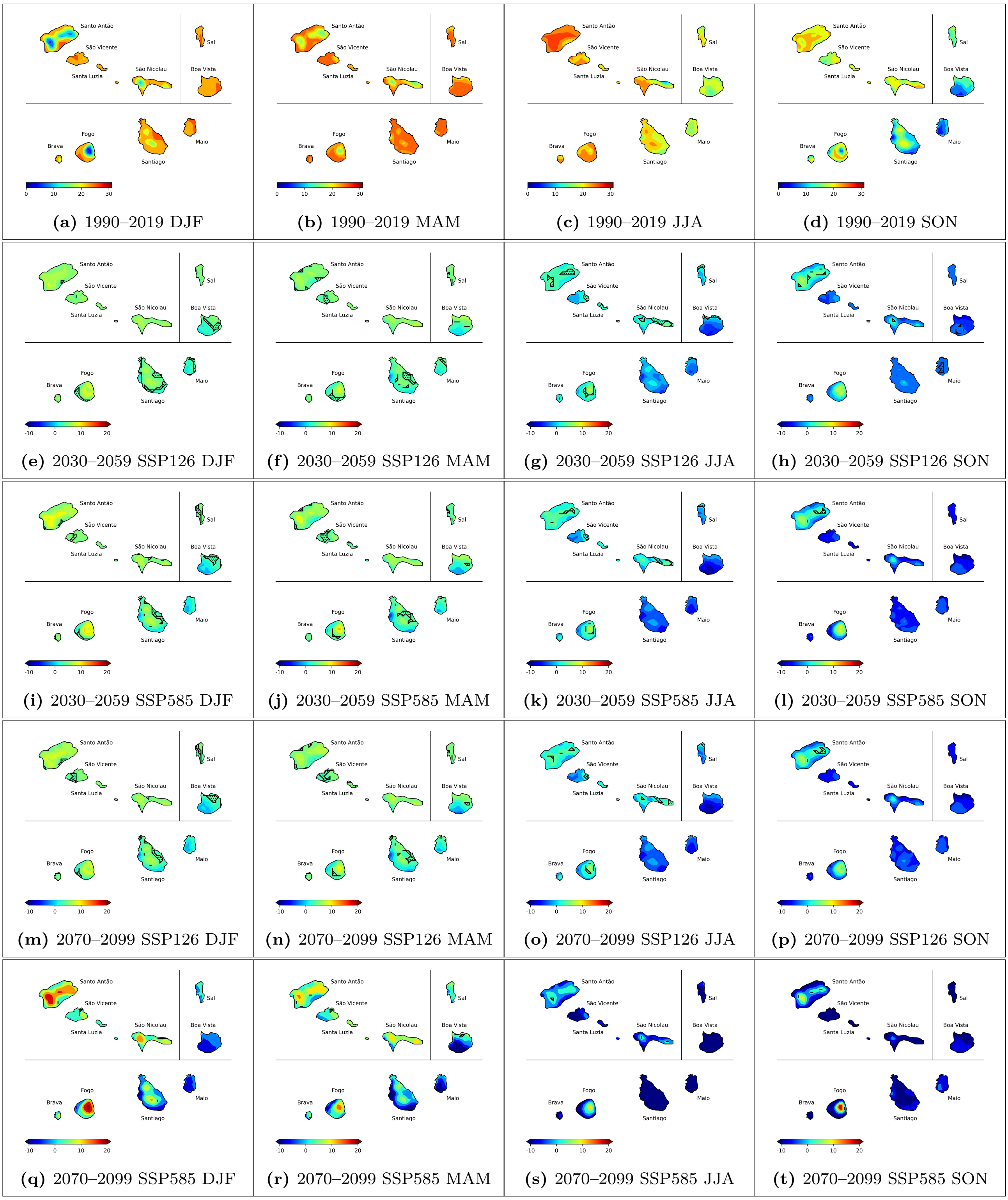

Figure S32. Simulated observations and projected changes in the **HCIU80** sub-index for **Cabo Verde**. The HCIU80 metric represents the number of excellent days for urban tourism. The top row shows simulated observed values from 1990–2019 for all four seasons (DJF, MAM, JJA, and SON). The subsequent rows indicate projected changes (average seasonal differences in the monthly number of excellent days) for 2030–2059 and 2070–2099 under both the SSP1-2.6 and SSP5-8.5 scenarios, respectively, all relative to the 1990–2019 baseline. Areas with black dots indicate statistically non-significant changes, while non-hatched areas indicate statistically significant changes. These non-significant results reflect variability in the ensemble response rather than the absence of change.

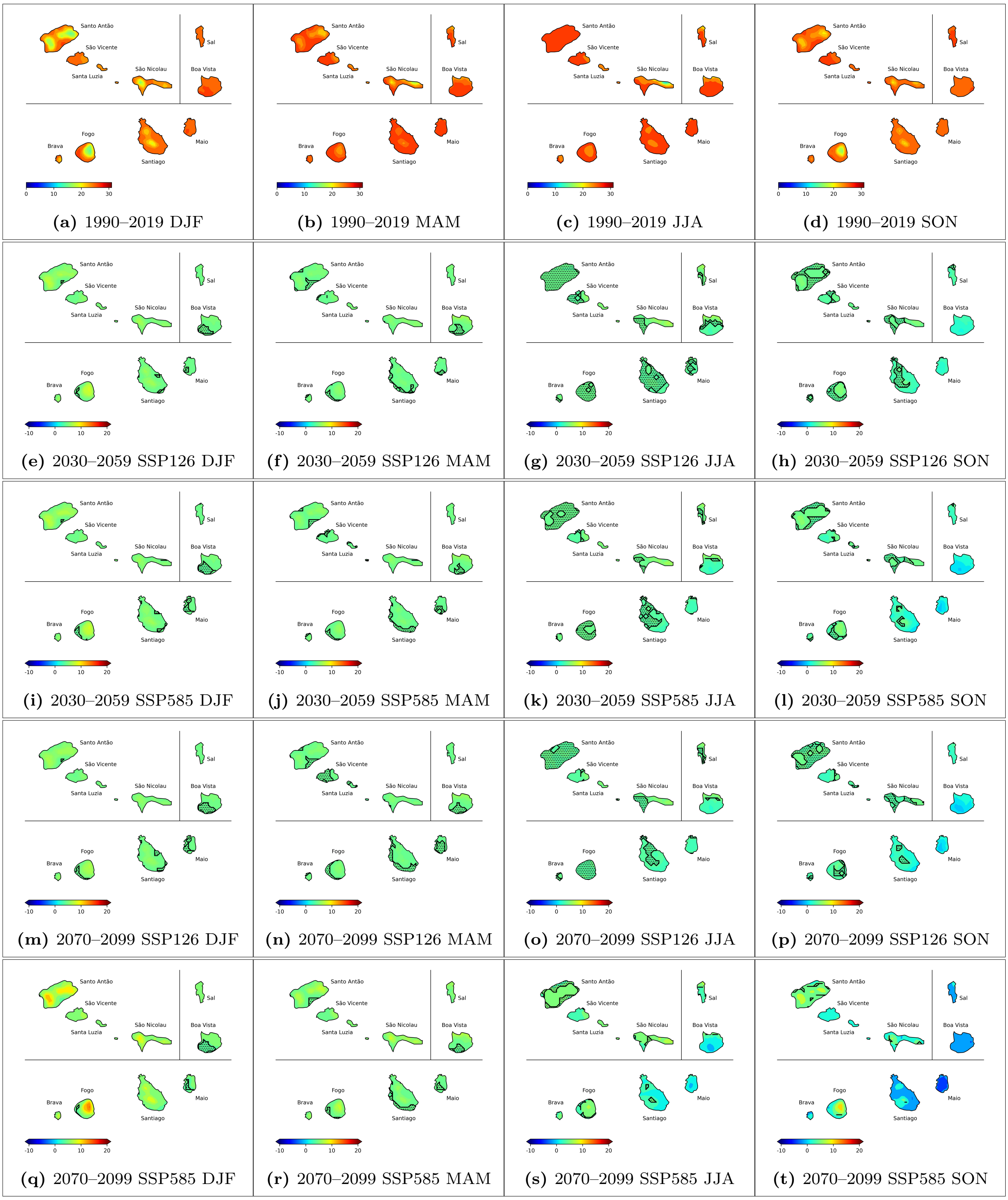

(a) 1990–2019 DJF
(b) 1990–2019 MAM
(c) 1990–2019 JJA
(d) 1990–2019 SON
(e) 2030–2059 SSP126 DJF
(f) 2030–2059 SSP126 MAM
(g) 2030–2059 SSP126 JJA
(h) 2030–2059 SSP126 SON
(i) 2030–2059 SSP585 DJF
(j) 2030–2059 SSP585 MAM
(k) 2030–2059 SSP585 JJA
(l) 2030–2059 SSP585 SON
(m) 2070–2099 SSP126 DJF
(n) 2070–2099 SSP126 MAM
(o) 2070–2099 SSP126 JJA
(p) 2070–2099 SSP126 SON
(q) 2070–2099 SSP585 DJF
(r) 2070–2099 SSP585 MAM
(s) 2070–2099 SSP585 JJA
(t) 2070–2099 SSP585 SON

Figure S33. Simulated observations and projected changes in the **HCIB60** sub-index for **Cabo Verde**. The HCIB60 metric represents the number of good days for beach tourism. The top row shows simulated observed values from 1990–2019 for all four seasons (DJF, MAM, JJA, and SON). The subsequent rows indicate projected changes (average seasonal differences in the monthly number of good days) for 2030–2059 and 2070–2099 under both the SSP1-2.6 and SSP5-8.5 scenarios, respectively, all relative to the 1990–2019 baseline. Areas with black dots indicate statistically non-significant changes, while non-hatched areas indicate statistically significant changes. These non-significant results reflect variability in the ensemble response rather than the absence of change.

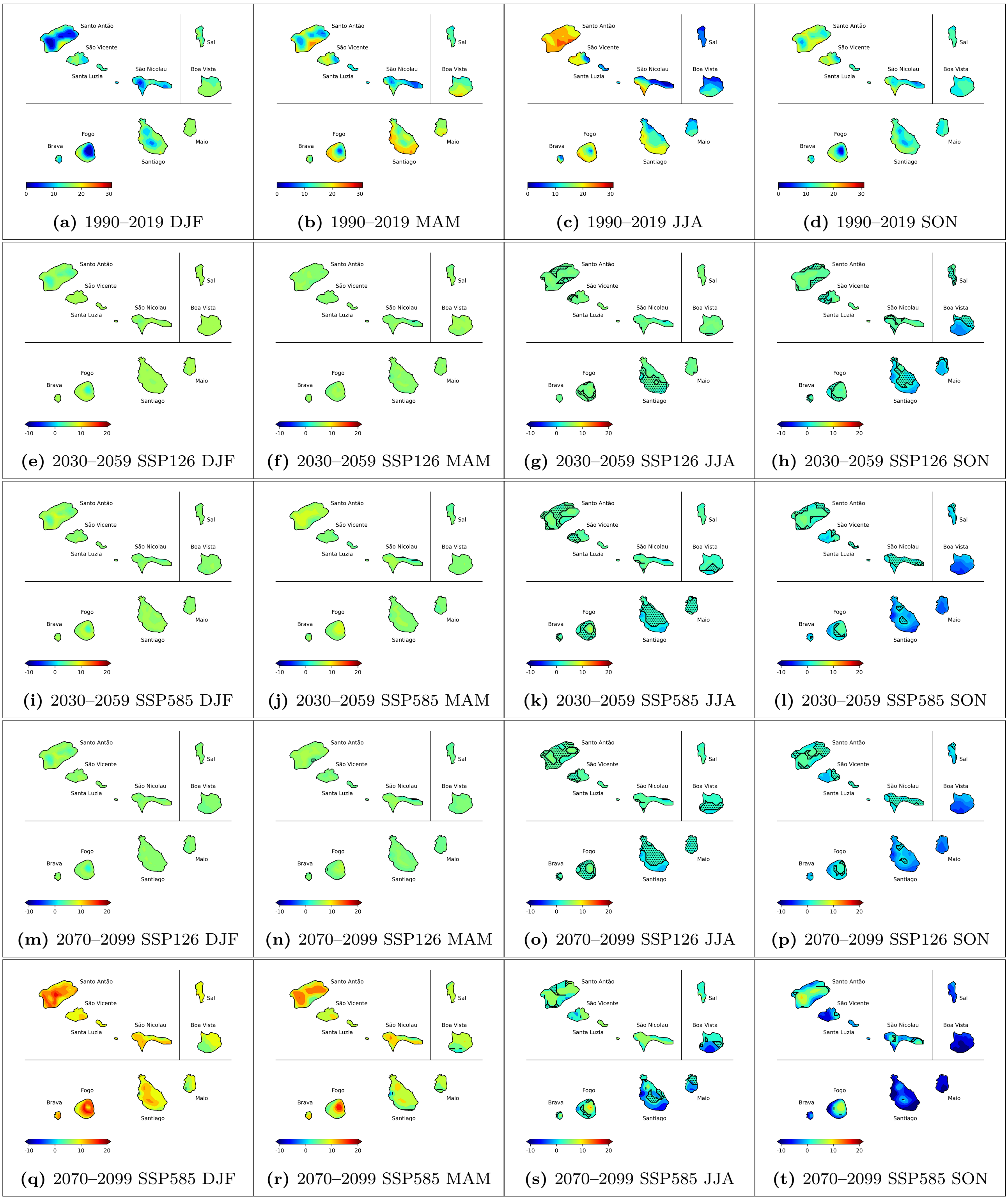

(a) 1990–2019 DJF **(b)** 1990–2019 MAM **(c)** 1990–2019 JJA **(d)** 1990–2019 SON

(e) 2030–2059 SSP126 DJF **(f)** 2030–2059 SSP126 MAM **(g)** 2030–2059 SSP126 JJA **(h)** 2030–2059 SSP126 SON

(i) 2030–2059 SSP585 DJF **(j)** 2030–2059 SSP585 MAM **(k)** 2030–2059 SSP585 JJA **(l)** 2030–2059 SSP585 SON

(m) 2070–2099 SSP126 DJF **(n)** 2070–2099 SSP126 MAM **(o)** 2070–2099 SSP126 JJA **(p)** 2070–2099 SSP126 SON

(q) 2070–2099 SSP585 DJF **(r)** 2070–2099 SSP585 MAM **(s)** 2070–2099 SSP585 JJA **(t)** 2070–2099 SSP585 SON

Figure S34. Simulated observations and projected changes in the **HCIB80** sub-index for **Cabo Verde**. The HCIB80 metric represents the number of excellent days for beach tourism. The top row shows simulated observed values from 1990–2019 for all four seasons (DJF, MAM, JJA, and SON). The subsequent rows indicate projected changes (average seasonal differences in the monthly number of excellent days) for 2030–2059 and 2070–2099 under both the SSP1-2.6 and SSP5-8.5 scenarios, respectively, all relative to the 1990–2019 baseline. Areas with black dots indicate statistically non-significant changes, while non-hatched areas indicate statistically significant changes. These non-significant results reflect variability in the ensemble response rather than the absence of change.

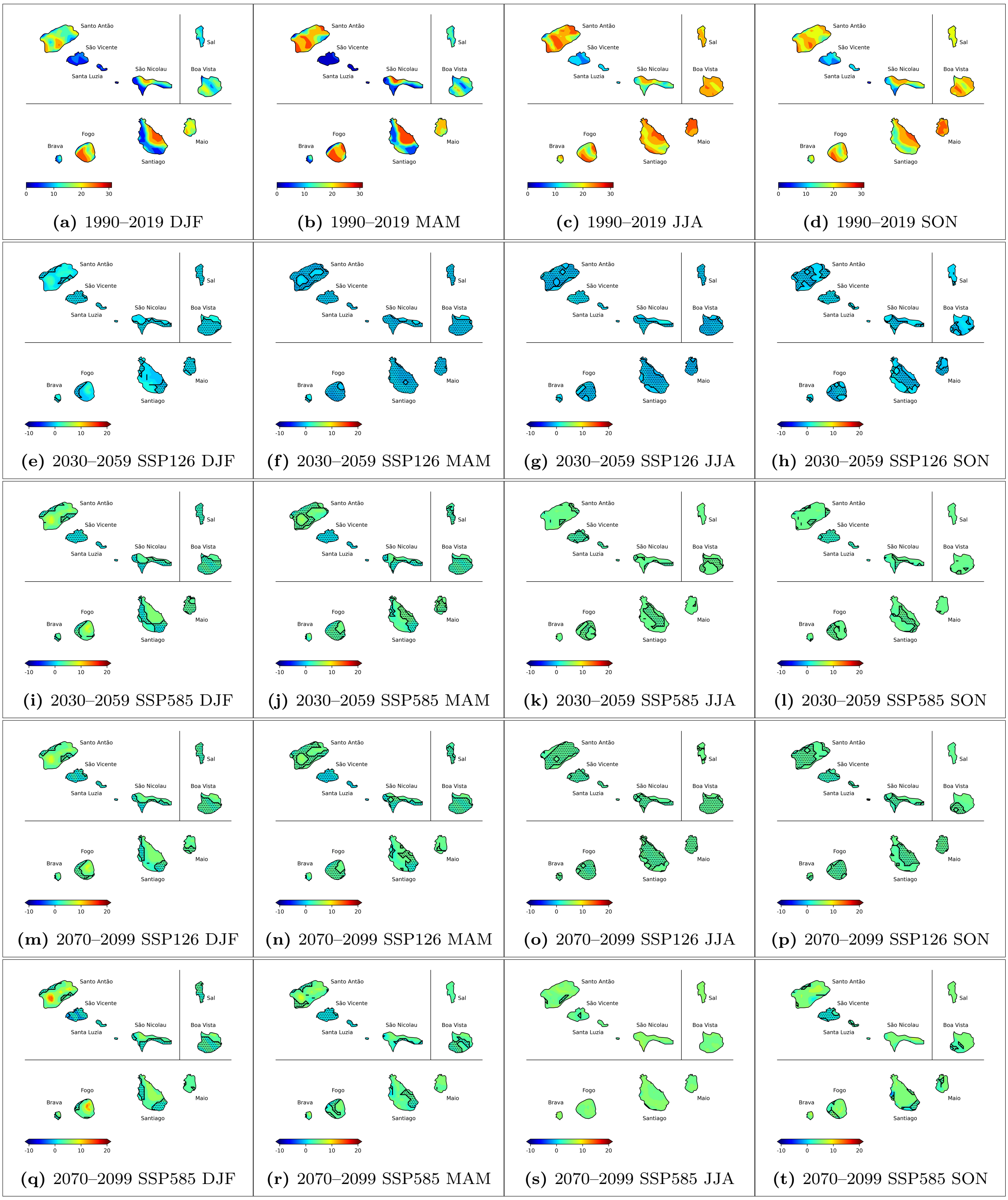

(a) 1990–2019 DJF
(b) 1990–2019 MAM
(c) 1990–2019 JJA
(d) 1990–2019 SON
(e) 2030–2059 SSP126 DJF
(f) 2030–2059 SSP126 MAM
(g) 2030–2059 SSP126 JJA
(h) 2030–2059 SSP126 SON
(i) 2030–2059 SSP585 DJF
(j) 2030–2059 SSP585 MAM
(k) 2030–2059 SSP585 JJA
(l) 2030–2059 SSP585 SON
(m) 2070–2099 SSP126 DJF
(n) 2070–2099 SSP126 MAM
(o) 2070–2099 SSP126 JJA
(p) 2070–2099 SSP126 SON
(q) 2070–2099 SSP585 DJF
(r) 2070–2099 SSP585 MAM
(s) 2070–2099 SSP585 JJA
(t) 2070–2099 SSP585 SON

Figure S35. Simulated observations and projected changes in the **CCI05** sub-index for **Cabo Verde**. The CCI05 metric represents the number of good days for nature-based tourism. The top row shows simulated observed values from 1990–2019 for all four seasons (DJF, MAM, JJA, and SON). The subsequent rows indicate projected changes (average seasonal differences in the monthly number of good days) for 2030–2059 and 2070–2099 under both the SSP1-2.6 and SSP5-8.5 scenarios, respectively, all relative to the 1990–2019 baseline. Areas with black dots indicate statistically non-significant changes, while non-hatched areas indicate statistically significant changes. These non-significant results reflect variability in the ensemble response rather than the absence of change.

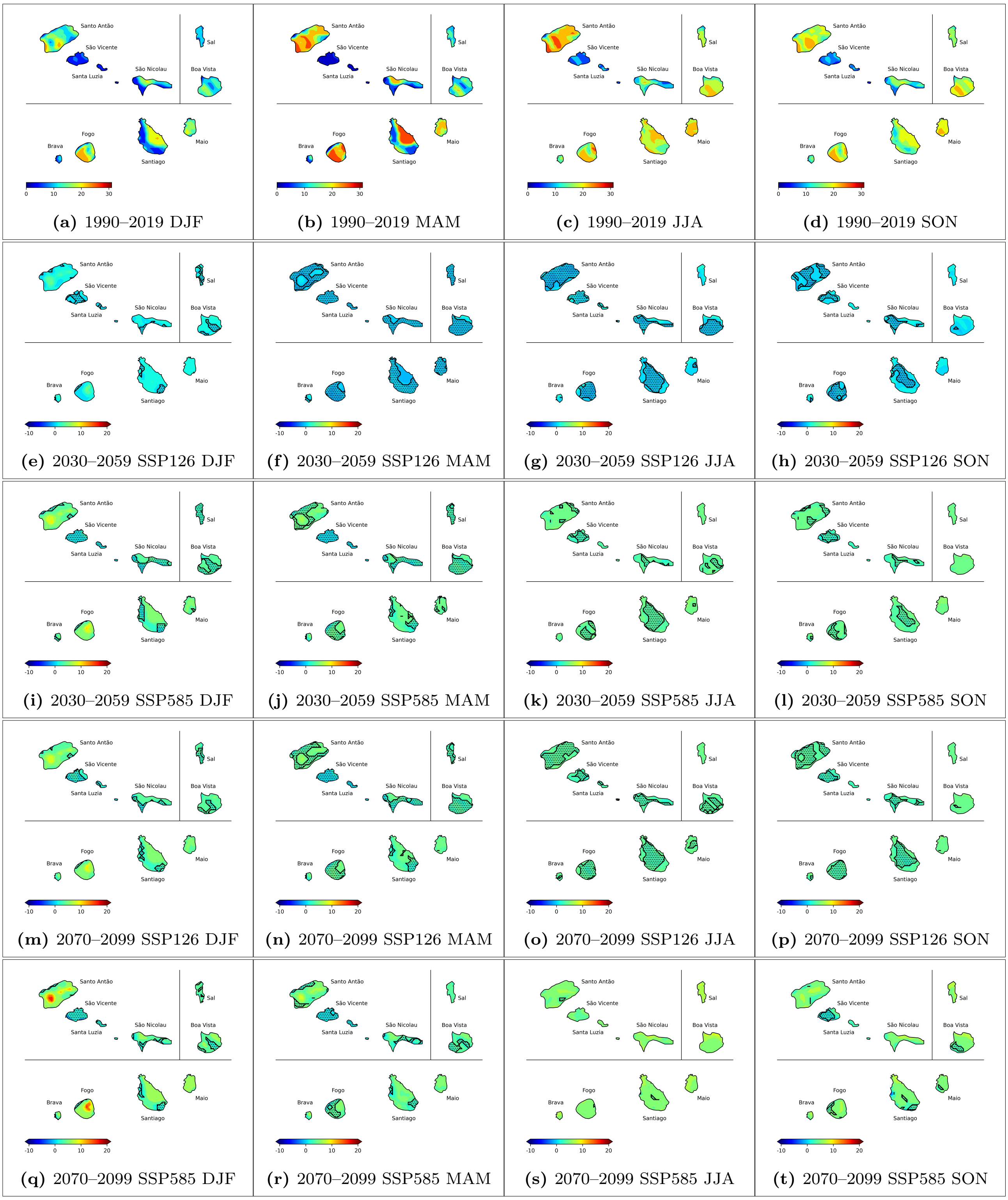

(a) 1990–2019 DJF **(b)** 1990–2019 MAM **(c)** 1990–2019 JJA **(d)** 1990–2019 SON

(e) 2030–2059 SSP126 DJF **(f)** 2030–2059 SSP126 MAM **(g)** 2030–2059 SSP126 JJA **(h)** 2030–2059 SSP126 SON

(i) 2030–2059 SSP585 DJF **(j)** 2030–2059 SSP585 MAM **(k)** 2030–2059 SSP585 JJA **(l)** 2030–2059 SSP585 SON

(m) 2070–2099 SSP126 DJF **(n)** 2070–2099 SSP126 MAM **(o)** 2070–2099 SSP126 JJA **(p)** 2070–2099 SSP126 SON

(q) 2070–2099 SSP585 DJF **(r)** 2070–2099 SSP585 MAM **(s)** 2070–2099 SSP585 JJA **(t)** 2070–2099 SSP585 SON

Figure S36. Simulated observations and projected changes in the **CCI07** sub-index for **Cabo Verde**. The CCI07 metric represents the number of optimal days for nature-based tourism. The top row shows simulated observed values from 1990–2019 for all four seasons (DJF, MAM, JJA, and SON). The subsequent rows indicate projected changes (differences in the average number of optimal days per month) for 2030–2059 and 2070–2099 under both the SSP1-2.6 and SSP5-8.5 scenarios, respectively, all relative to the 1990–2019 baseline. Areas with black dots indicate statistically non-significant changes, while non-hatched areas indicate statistically significant changes. These non-significant results reflect variability in the ensemble response rather than the absence of change.

4 Summary of climate projections for tourism in the Macaronesian archipelagos

The data consistently projects an overall increase in tourism suitability across all four archipelagos. The most relevant improvements are projected for the northern archipelagos (the Azores and Madeira) and for the off-season in the southern archipelagos (the Canary Islands and Cabo Verde), although there are important exceptions. These positive trends are particularly pronounced under the high-emissions SSP5-8.5 scenario.

For good general tourism conditions (TCI60), all four archipelagos already have a high number of favorable days. Projections indicate a widespread increase of up to 6 additional days per month for all islands, with some areas under the high-emissions SSP5-8.5 scenario revealing even greater increases of up to 6 days in winter and spring. When it comes to excellent tourism conditions (TCI80), the trends indicate a clear divergence. The northern archipelagos (the Azores and Madeira), which currently have a shorter season for excellent days, are projected to see a substantial increase in these conditions, particularly in spring and autumn. The southern archipelagos (the Canary Islands and Cabo Verde), already well-suited for tourism year-round, will see a continued intensification of these excellent conditions. The SSP5-8.5 scenario leads to a notable increase in the Canary Islands, with up to 11 days in winter and spring, but a notable decrease for Cabo Verde, with a projected loss of up to 16 days in autumn.

All four archipelagos currently experience a high number of good urban tourism days (HCIU60) in all seasons. Projected changes for the SSP5-8.5 scenario indicate a widespread increase of up to 4 days per month across the board, with some areas revealing even greater changes in summer and autumn. For excellent urban tourism conditions (HCIU80), the data shows an even greater projected improvement for some areas. While the northern archipelagos will see an increase of up to 9 days per month, the southern archipelagos are projected to see a mixed but relevant change. This is especially true for the Canary Islands and Cabo Verde under SSP5-8.5, which projects an increase of up to 8 days per month in winter for the Canary Islands, but a dramatic decrease for Cabo Verde, with a projected loss of up to 12 days in autumn.

There is a consistent trend of increasing good beach tourism conditions (HCIB60) for all four island groups. Both SSP scenarios project a widespread increase of up to 5 days per month. However, the high-emissions SSP5-8.5 scenario is projected to lead to even greater improvements, with some areas revealing an increase of more than 5 days in autumn and winter by the end of the century. Excellent beach tourism conditions (HCIB80) follow a similar pattern, with the SSP5-8.5 scenario projecting the most dramatic and widespread improvements across all archipelagos. The data for the northern archipelagos indicates a lengthening of the season for excellent beach days, while the Canary Islands are projected to see an intensification of these conditions, making winter and autumn more suitable for beach activities. In contrast, the Cabo Verde archipelago is projected to see a decrease in excellent beach tourism days in autumn.

For both good (CCI05) and optimal (CCI07) nature-based tourism, the trends are quite clear. The low-emissions SSP1-2.6 scenario projects mostly minor changes across all archipelagos, with some areas even experiencing small decreases, such as up to 5 days in summer for the Azores. In contrast, the high-emissions SSP5-8.5 scenario projects a widespread increase in suitable days. This is particularly notable in winter and autumn, where some areas are projected to see increases of up to 5 days per month. For the northern archipelagos (the Azores and Madeira), changes under SSP5-8.5 are generally small in the near-term, but for Cabo Verde, this scenario projects a widespread increase of up to 4 days for good conditions and up to 6 days for optimal conditions, especially in summer and autumn. The Canary Islands, however, project a more mixed trend with small decreases of up to 1 day in the near term for both good and optimal conditions.

References

[1] Jordi Rodriguez-Rull, Francisco Javier Expósito González, Juan P Diaz, Judit Carrillo, Juan Carlos Pérez Darias, and Diamantino Henriques. Tourism Index Data for Macaronesia, 2026. URL `https://data.mendeley.com/datasets/5xrst4z9gk`.

[2] J Carrillo, A González, J C Pérez, F J Expósito, and J P Díaz. Projected impacts of climate change on tourism in the Canary Islands. *Regional Environmental Change*, 22(2), 2022. doi: 10.1007/s10113-022-01880-9.

List of Figures

S1 Validation of TCI60, HCIU60, HCIB60, CCI05: monthly mean plots 3
S2 Validation of TCI80, HCIU80, HCIB80, CCI07: monthly mean plots 4
S3 Validation of TCI60, HCIU60, HCIB60, CCI05: Q-Q plots 5
S4 Validation of TCI80, HCIU80, HCIB80, CCI07: Q-Q plots 6
S5 Projected changes in TCI60 for the Azores 8
S6 Projected changes in TCI80 for the Azores 9

S7 Projected changes in HCIU60 for the Azores . . . 10
S8 Projected changes in HCIU80 for the Azores . . . 11
S9 Projected changes in HCIB60 for the Azores . . . 12
S10 Projected changes in HCIB80 for the Azores . . . 13
S11 Projected changes in CCI05 for the Azores . . . 14
S12 Projected changes in CCI07 for the Azores . . . 15
S13 Projected changes in TCI60 for Madeira . . . 17
S14 Projected changes in TCI80 for Madeira . . . 18
S15 Projected changes in HCIU60 for Madeira . . . 19
S16 Projected changes in HCIU80 for Madeira . . . 20
S17 Projected changes in HCIB60 for Madeira . . . 21
S18 Projected changes in HCIB80 for Madeira . . . 22
S19 Projected changes in CCI05 for Madeira . . . 23
S20 Projected changes in CCI07 for Madeira . . . 24
S21 Projected changes in TCI60 for the Canary Islands . . . 26
S22 Projected changes in TCI80 for the Canary Islands . . . 27
S23 Projected changes in HCIU60 for the Canary Islands . . . 28
S24 Projected changes in HCIU80 for the Canary Islands . . . 29
S25 Projected changes in HCIB60 for the Canary Islands . . . 30
S26 Projected changes in HCIB80 for the Canary Islands . . . 31
S27 Projected changes in CCI05 for the Canary Islands . . . 32
S28 Projected changes in CCI07 for the Canary Islands . . . 33
S29 Projected changes in TCI60 for Cabo Verde . . . 35
S30 Projected changes in TCI80 for Cabo Verde . . . 36
S31 Projected changes in HCIU60 for Cabo Verde . . . 37
S32 Projected changes in HCIU80 for Cabo Verde . . . 38
S33 Projected changes in HCIB60 for Cabo Verde . . . 39
S34 Projected changes in HCIB80 for Cabo Verde . . . 40
S35 Projected changes in CCI05 for Cabo Verde . . . 41
S36 Projected changes in CCI07 for Cabo Verde . . . 42

List of Tables

S1 Validation stations and model grid-points comparisons . . . 2
S2 Summary of projected changes for the Azores . . . 7
S3 Summary of projected changes for Madeira . . . 16
S4 Summary of projected changes for the Canary Islands . . . 25
S5 Summary of projected changes for Cabo Verde . . . 34
